# RUNX2 promotes osteoclast differentiation and bone resorption via the newly identified *LncRNA-ROD*: implications for alveolar remodeling

**DOI:** 10.1038/s41368-026-00466-z

**Published:** 2026-09-28

**Authors:** Jie Li, Yang Liu, Yanchun Qiao, Changqing Yan, Weiwei Zhao, Lingli Ji, Dandan Liu, Yuejiao Xin, Chenying Zhang, Xiaozhe Wang, Yixiang Wang, Shuguo Zheng

**Affiliations:** 1 Department of Preventive Dentistry, Peking University School and Hospital of Stomatology & National Center of Stomatology & National Clinical Research Center for Oral Diseases & National Engineering Research Center of Oral Biomaterials and Digital Medical Devices, Beijing, China; 2 Department of Stomatology, Aerospace Center Hospital, School of Aerospace Clinical Medicine, Peking University, Beijing, China; 3 Department of Periodontics, Tianjin Stomatological Hospital, School of Medicine, Nankai University, Tianjin, China; 4 Department of Pediatric Dentistry, Peking University School and Hospital of Stomatology & National Center of Stomatology & National Clinical Research Center for Oral Diseases & National Engineering Research Center of Oral Biomaterials and Digital Medical Devices, Beijing, China

**Keywords:** Mechanisms of disease, Oral diseases

## Abstract

Delayed tooth eruption is a hallmark of cleidocranial dysplasia (CCD) caused by RUNX2 haploinsufficiency, yet the underlying molecular mechanism remains undefined. Here, we show that RUNX2 regulates osteoclast-mediated alveolar bone remodeling, a process closely associated with tooth eruption. *Runx2*^+/−^ mice exhibit impaired osteoclast differentiation and reduced alveolar bone resorption, resulting in delayed mandibular molar eruption—a phenotype that recapitulates human CCD. Mechanistically, RUNX2 transcriptionally activates *LncRNA-ROD* (*Regulator of Osteoclast Differentiation*), which promotes osteoclastogenesis by sequestering QKI to stabilize *Csf1r* mRNA. *LncRNA-ROD*^−/−^ mice phenocopy the eruption defects and reduced alveolar bone resorption observed in *Runx2*^+/−^ mice. Critically, both *Runx2*^+/−^ and *LncRNA-ROD*^−/−^ mice show reduced osteoclast differentiation in alveolar bone, consistent with in vitro data from bone marrow-derived monocytes/macrophages demonstrating impaired osteoclast differentiation. Notably, rescue experiments in ovariectomy-induced osteoporosis models demonstrate that QKI knockdown restores osteoclast differentiation in *Runx2*^+/−^ and *LncRNA-ROD*^−/−^ mice, highlighting the critical role of the RUNX2/*LncRNA-ROD*/QKI axis in osteoclastogenesis. The axis supports alveolar bone remodeling associated with tooth eruption. These results provide mechanistic insights into cleidocranial dysplasia-related alveolar bone remodeling defects and delayed tooth eruption, and identify *LncRNA-ROD* as a potential therapeutic target for alveolar bone remodeling disorders. Furthermore, this axis is implicated in broader bone metabolic disorders, as demonstrated by its impact on osteoporosis progression in *Runx2*^+/−^ and *LncRNA-ROD*^−/−^ mice.

## Introduction

RUNX2 has long been recognized as an osteoblast-specific transcription factor essential for osteoblast differentiation.^1–3^ Mutations or haploinsufficiency of RUNX2 cause cleidocranial dysplasia (CCD), a skeletal disorder characterized by generalized skeletal abnormalities, delayed eruption of permanent teeth, and supernumerary teeth.^4–8^ Emerging evidence indicates that RUNX2 indirectly regulates osteoclast differentiation and bone resorption, though its direct molecular mechanisms remain elusive.^9–13^ Our previous work demonstrated that RUNX2 promotes osteoclast differentiation and bone resorption by modulating the AKT/NFATc1/CTSK signaling axis.^14^ However, the direct regulatory role of RUNX2 in osteoclast differentiation and bone resorption and its underlying molecular mechanisms remain poorly understood.

Clinical observations revealed that CCD patients exhibited increased bone resistance during surgical exposure of impacted teeth. Micro-CT analysis and TRAP staining further demonstrated that RUNX2 mutations impair osteoclast differentiation and bone resorption in alveolar bone.^14^ Notably, our preliminary studies showed impaired osteoclastogenesis in RUNX2 knockout RAW 264.7 cells, suggesting a potential direct regulatory function of RUNX2 in osteoclast differentiation and bone resorption. These findings establish CCD as a natural model of abnormal bone remodeling. This study therefore utilizes CCD as a model system to investigate the regulatory role of RUNX2 in osteoclast differentiation and bone resorption and elucidate its molecular mechanisms, which may provide novel therapeutic targets for treating tooth eruption disorders and other bone metabolic diseases associated with osteoclast dysfunction.

Beyond transcription factors, accumulating evidence highlights critical regulatory roles of long non-coding RNAs (lncRNAs) in osteoclast differentiation and bone resorption.^15,16^ For instance, lncRNA *MALAT1* inhibits osteoclast differentiation by binding to TEAD3 and preventing its interaction with the NFATc1 promoter, thereby suppressing downstream gene expression.^17^ Similarly, lncRNA *Nron* facilitates nuclear translocation of the NF-κB repressing factor, which inhibits NFATc1 transcription and ultimately suppressing osteoclastogenesis and alveolar bone resorption in periodontitis.^18^ Targeting specific lncRNAs has shown therapeutic potential in disease models with aberrant osteoclast activity. For example, lncRNA *FGF14-AS2* suppresses osteoclastogenesis and bone metastasis in breast cancer models by inhibiting eIF4E/eIF4G complex formation and eIF4E phosphorylation, thereby reducing RANKL transcriptione.^19^ Additionally, lncRNA *SOX2OT* functions as a competing endogenous RNA for *miR-194-5p*, relieving RAC1 repression and promoting osteolytic metastasis in non-small cell lung cancer.^20^ Collectively, these findings underscore the pivotal roles of lncRNAs in osteoclast differentiation and bone remodeling, suggesting their potential as therapeutic targets. However, the specific functions of lncRNAs in RUNX2-mediated regulation of osteoclastogenesis remain largely uncharacterized.

To elucidate the direct role of RUNX2 in osteoclast differentiation and bone resorption, and to explore lncRNA involvement in this regulatory pathway, we conducted comprehensive in vitro and in vivo experiments using animal models. Our findings demonstrate that RUNX2 directly promotes osteoclast differentiation and bone resorption, whereas RUNX2 haploinsufficiency suppresses osteoclast differentiation and bone resorption, thereby alleviating bone loss in ovariectomized mice. Importantly, we identified a novel bone-specific lncRNA, designated *LncRNA-ROD*, whose transcription is directly activated by RUNX2. Functional studies revealed that silencing *LncRNA-ROD* impairs osteoclast differentiation and bone resorption, indicating its essential role in these processes. Mechanistically, *LncRNA-ROD* interacts with the RNA-binding protein QKI to regulate *Csf1r* mRNA stability. These findings establish RUNX2 and *LncRNA-ROD* as promising therapeutic targets for osteoclast dysfunction-associated diseases, particularly highlighting their potential in treating tooth eruption disorders and systemic bone metabolic disorders including osteoporosis through precise modulation of osteoclastogenesis and bone resorption.

## Results

### RUNX2 deficiency causes delayed tooth eruption and impaired osteoclast differentiation

**Fig. 1:**
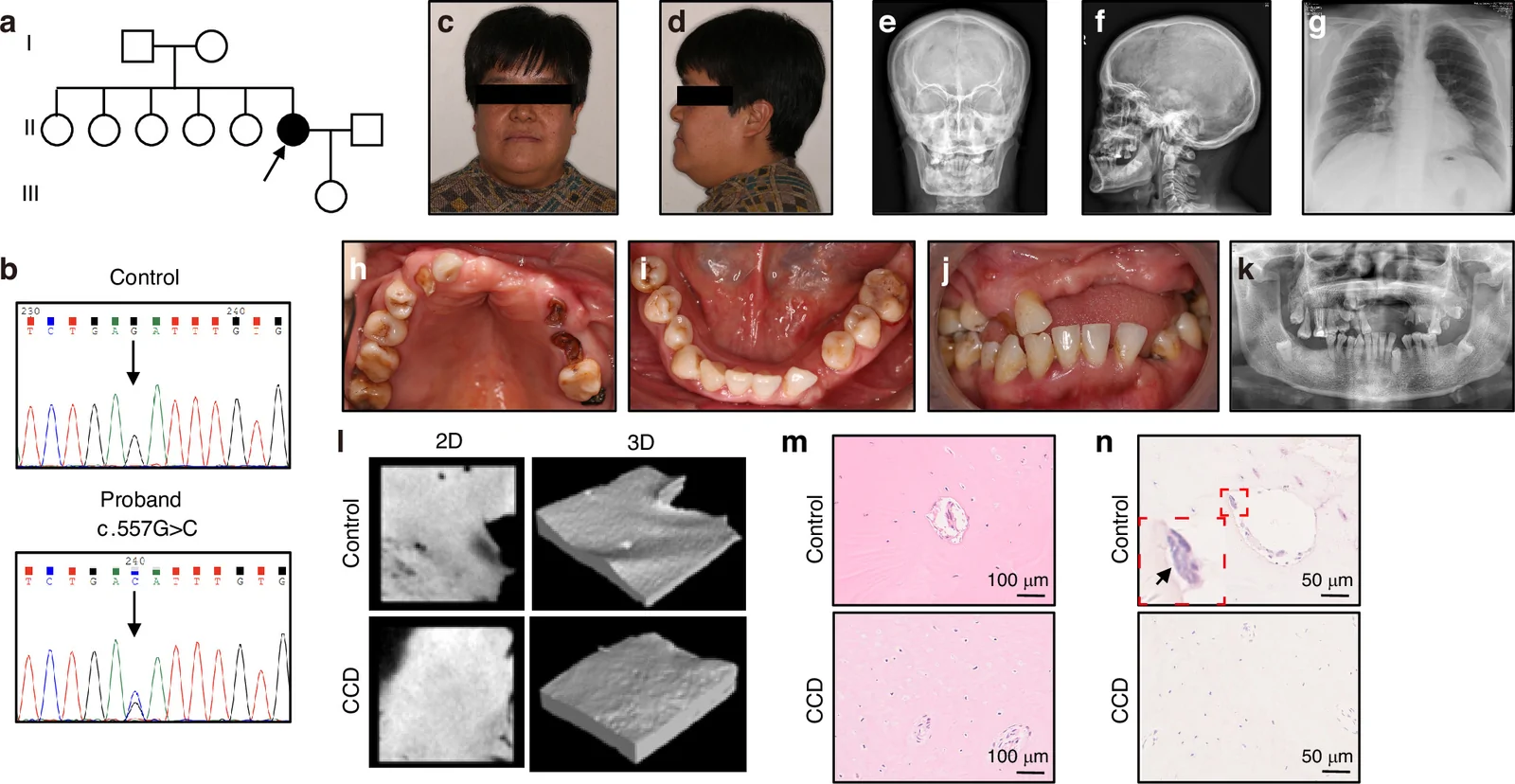
Clinical and genetic features of the CCD patient. **a** Family pedigree of the CCD proband (indicated by an arrow). **b** Sanger sequencing of the *RUNX2* gene in the control and the proband. Frontal view (**c**), lateral view (**d**), anteroposterior skull radiograph (**e**), lateral skull radiograph (**f**), chest X-ray (**g**), intraoral view (**h**–**j**), and panoramic radiograph (**k**) of the proband. **l** Representative images of micro-CT analysis of alveolar bone fragments from the CCD patient and healthy controls. **m** Representative HE staining images of the alveolar bone from the CCD patient and healthy controls. **n** Representative TRAP staining images showing TRAP-positive osteoclasts in the alveolar bone from the CCD patient and healthy controls

The proband, a 42-year-old woman from a nonconsanguineous Chinese family, exhibited a classic CCD phenotype, including delayed or non-closure of fontanelles, cranial suture widening, clavicular hypoplasia or complete absence, supernumerary teeth, retained deciduous teeth, and impacted or delayed eruption of permanent teeth. None of her relatives exhibited similar traits (Fig. 1a). Sanger sequencing identified a de novo missense mutation in exon 2 of the *RUNX2* gene (c.557 G > C, R186T) (Fig. 1b). Clinically, she presented with a broad forehead (Fig. 1c), midface hypoplasia (Fig. 1d), cranial suture widening (Fig. 1e, f), and hypoplastic clavicles (Fig. 1g). Intraoral examination revealed multiple missing permanent teeth and unerupted lower left and upper right canines, and two supernumerary teeth in the right maxillary premolar region (Fig. 1h–k). Surgical removal of the supernumerary teeth and associated cysts revealed dense alveolar bone surrounding the impacted teeth, which made extraction challenging. Micro-CT scans confirmed higher alveolar bone density in the CCD patient compared to age- and sex-matched controls (Fig. 1l and Table S1). Histological analysis of alveolar bone from the CCD patient via HE and TRAP staining revealed an absence of TRAP-positive osteoclasts (Fig. 1m, n), suggesting impaired osteoclast differentiation and bone resorption. These findings indicate that the *RUNX2* mutation disrupts osteoclast differentiation and bone resorption in the coronal alveolar bone of the tooth germ, potentially causing delayed tooth eruption in CCD patients.

**Fig. 2:**
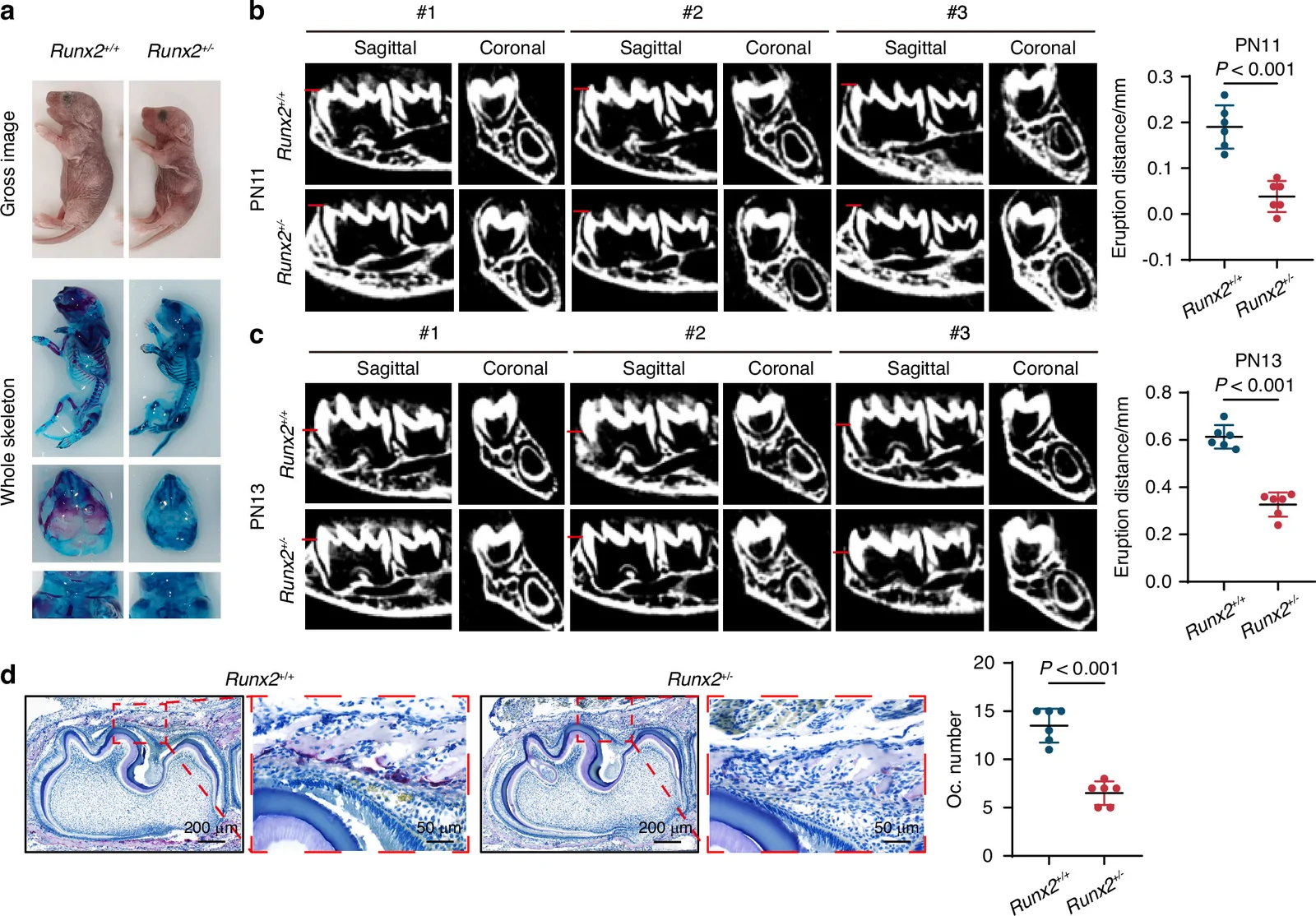
*Runx2*^+/-^ mice exhibit a delayed tooth eruption phenotype. **a** Alizarin red-alcian blue staining of skeletal frameworks in *Runx2*^+/−^ mice at PN0. **b**, **c** Representative sagittal and coronal views. Eruption levels were quantified in the sagittal plane by measuring the vertical distance from the highest cusp tip to the highest point of the alveolar crest on the mesial side of the mandibular first molars in *Runx2*^+/-^ mice at PN11 (*n* = 6) (**b**) and PN13 (*n* = 6) (**c**). Red lines indicate the highest point of the alveolar crest. The eruption level was measured as the vertical distance from this point to the highest cusp tip. **d** TRAP staining images and quantification of TRAP-positive osteoclasts in *Runx2*^+/−^ mice at PN5 (*n* = 6). Data are presented as mean ± SD

Delayed eruption of permanent teeth is a hallmark clinical feature of CCD and a major reason for clinical visits. To investigate the role of RUNX2 in osteoclast differentiation and bone resorption, we utilized mouse models. Given the early lethality of *Runx2*^−/−^ mice due to respiratory failure from ossification defects,^21^ we employed *Runx2*^+/−^ mice, generated via homologous recombination. *Runx2*^+/−^ mice, regardless of sex, exhibited CCD-like features, including hypoplastic clavicles and delayed fontanel closure (Fig. 2a). Since *Runx2*^*+/−*^ mice recapitulate the human CCD phenotype, we used this model to assess mandibular first molar eruption dynamics using micro-CT. Micro-CT analysis of mandibles from postnatal day 11 (PN11) and PN13 mice revealed a marked delay in molar eruption in *Runx2*^*+/−*^ mice compared with wild-type controls (Fig. 2b, c). TRAP staining of PN5 mandibles showed reduced numbers of TRAP-positive osteoclasts in the alveolar bone surrounding the molar crown in *Runx2*^*+/−*^ mice (Fig. 2d), indicating defective bone resorption.

To assess whether the eruption delay persisted as tooth development progressed, we next examined mandibular molars at PN21, when the first (M1) and second (M2) molars have typically achieved occlusion. At this stage, M1 and M2 in *Runx2*^+/−^ mice showed a small but statistically significant reduction in eruption height compared with wild-type mice; however, 3D reconstruction revealed that the eruption heights were largely comparable between the two genotypes (Fig. S1). Notably, the third (M3) molar had not yet achieved occlusion in either genotype, and the eruption height of M3 was markedly lower in *Runx2*^+/−^ mice than in wild-type controls (Fig. S1). These findings indicate that the eruption delay observed at PN11–13 partially resolves for M1 and M2 by 3 weeks of age, whereas M3 remains substantially affected, indicating a sustained impairment in alveolar bone remodeling.

**Fig. 3:**
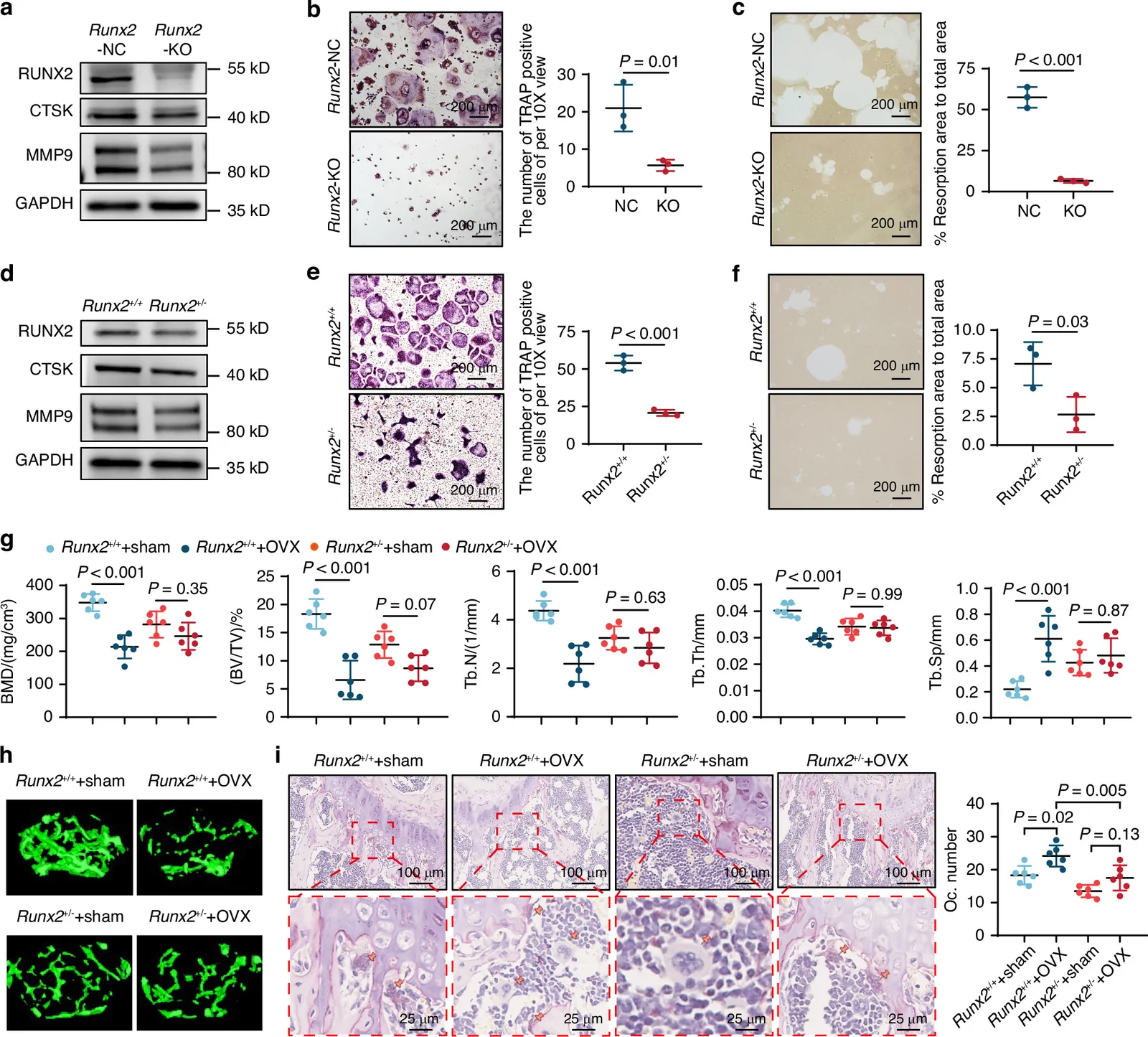
RUNX2 knockout inhibits osteoclast differentiation and bone resorption. **a** Western blot analysis of osteoclast-related gene expression in *Runx2* KO cells. Representative images of TRAP staining (**b**) and bone resorption pits (**c**) in *Runx2* KO cells (*n* = 3). **d** Western blot analysis of osteoclast-related gene expression in *Runx2*^+/-^ BMMs. Representative images of TRAP staining (**e**) and bone resorption pits (**f**) in *Runx2*^+/-^ BMMs (*n* = 3). **g** Micro-CT analysis of trabecular bone parameters in the distal femur (*n* = 6). **h** Representative three-dimensional reconstruction images of the distal femur trabecular structure. **i** Representative images of TRAP staining in femoral sections (*n* = 6). Data are presented as mean ± SD

To directly assess the regulatory role of RUNX2 in osteoclast differentiation and bone resorption, we generated RUNX2 knockout RAW 264.7 cells (*Runx2* KO) using the CRISPR/Cas9 system (Fig. S2a). Sanger sequencing confirmed a frameshift mutation (c.451_611del, p.151 fs) in the *Runx2* coding region (NM_001146038.2) (Fig. S2b), resulting in a truncated protein of 113 amino acids (Fig. S2c), of which only the first 46 amino acids aligned with the RUNX2 sequence. Compared to control (*Runx2* NC) cells, *Runx2* KO cells exhibited significantly lower mRNA and protein levels of osteoclast-related genes during differentiation, as shown by RT-qPCR (Fig. S2d) and Western blot analysis (Fig. 3a, Fig. S2e) on days 3 and 4 of osteoclast induction. TRAP staining and resorption pit assays on days 4 and 6 revealed a notable decrease in TRAP-positive osteoclasts (Fig. 3b) and the resorption pit area (Fig. 3c) in *Runx2* KO cells. These findings collectively indicate that loss of RUNX2 impaired the ability of RAW 264.7 cells to differentiate into osteoclasts, demonstrating that RUNX2 plays a crucial role in promoting osteoclast differentiation and bone resorption.

Next, we isolated bone marrow-derived monocytes/macrophages (BMMs) from *Runx2*^+/+^ and *Runx2*^+/-^ mice and induced osteoclast differentiation. On days 3 and 4, RT-qPCR (Fig. S2f) and Western blot analysis (Fig. 3d, Fig. S2g) revealed significantly reduced expression of osteoclast-related genes in the *Runx2*^+/-^ BMMs compared to *Runx2*^+/+^ BMMs. TRAP staining and resorption assays performed on days 4 and 7 showed a marked reduction in TRAP-positive osteoclasts (Fig. 3e) and a significant decrease in the resorption pit area (Fig. 3f) in *Runx2*^+/-^ BMMs, validating the pivotal role of RUNX2 in promoting osteoclast differentiation and bone resorption.

To further confirm the role of RUNX2 in osteoclastogenesis under pathological conditions, we utilized a widely accepted model of postmenopausal osteoporosis induced by bilateral ovariectomy (OVX).^22^ Eight weeks post-OVX, we performed micro-CT scans on femurs from four groups: *Runx2*^+/+^+sham, *Runx2*^+/+^+OVX, *Runx2*^+/-^+sham, and *Runx2*^+/-^+OVX, to evaluate bone morphology and assess the effects of RUNX2 haploinsufficiency on bone loss induced by estrogen deficiency (Fig. S2h). Compared to the *Runx2*^+/+^+sham group, the *Runx2*^+/+^+OVX group exhibited significantly lower bone mineral density (BMD), bone volume/total volume (BV/TV), and greater trabecular separation (Tb.Sp), confirming the successful establishment of the OVX model (Fig. 3g). In contrast, the OVX group in *Runx2*^+/-^ mice showed slight decreases in some morphological parameters compared to the sham group, with no significant differences between the two groups (Fig. 3g). Three-dimensional reconstruction of the distal femur revealed that the trabecular structure in the *Runx2*^+/-^+OVX group was less developed than in the *Runx2*^+/-^+sham group but more developed than in the *Runx2*^*+/+*^+OVX group (Fig. 3h). These findings suggest that RUNX2 haploinsufficiency partially mitigates bone loss induced by estrogen deficiency, thereby alleviating the osteoporotic phenotype.

Histological analysis via HE and TRAP staining further assessed bone morphology and osteoclast differentiation. HE staining showed that the trabecular structure in the *Runx2*^+/-^+OVX group was less developed than in the *Runx2*^+/-^+sham group but more developed than in the *Runx2*^+/+^+OVX group (Fig. S2i). Additionally, TRAP staining revealed that the number of TRAP-positive osteoclasts in the *Runx2*^+/-^+OVX group was comparable to that in the *Runx2*^+/-^+sham group but significantly lower than in the *Runx2*^+/+^+OVX group (Fig. 3i). These results indicate that the preserved bone volume in ovariectomized *Runx2*^+/-^ mice is attributable to impaired osteoclast differentiation and bone resorption, rather than enhanced osteogenesis, as the number of TRAP-positive osteoclasts was significantly reduced compared with WT OVX controls.

### RUNX2 transcriptionally activates *LncRNA-ROD* to promote osteoclastogenesis

**Fig. 4:**
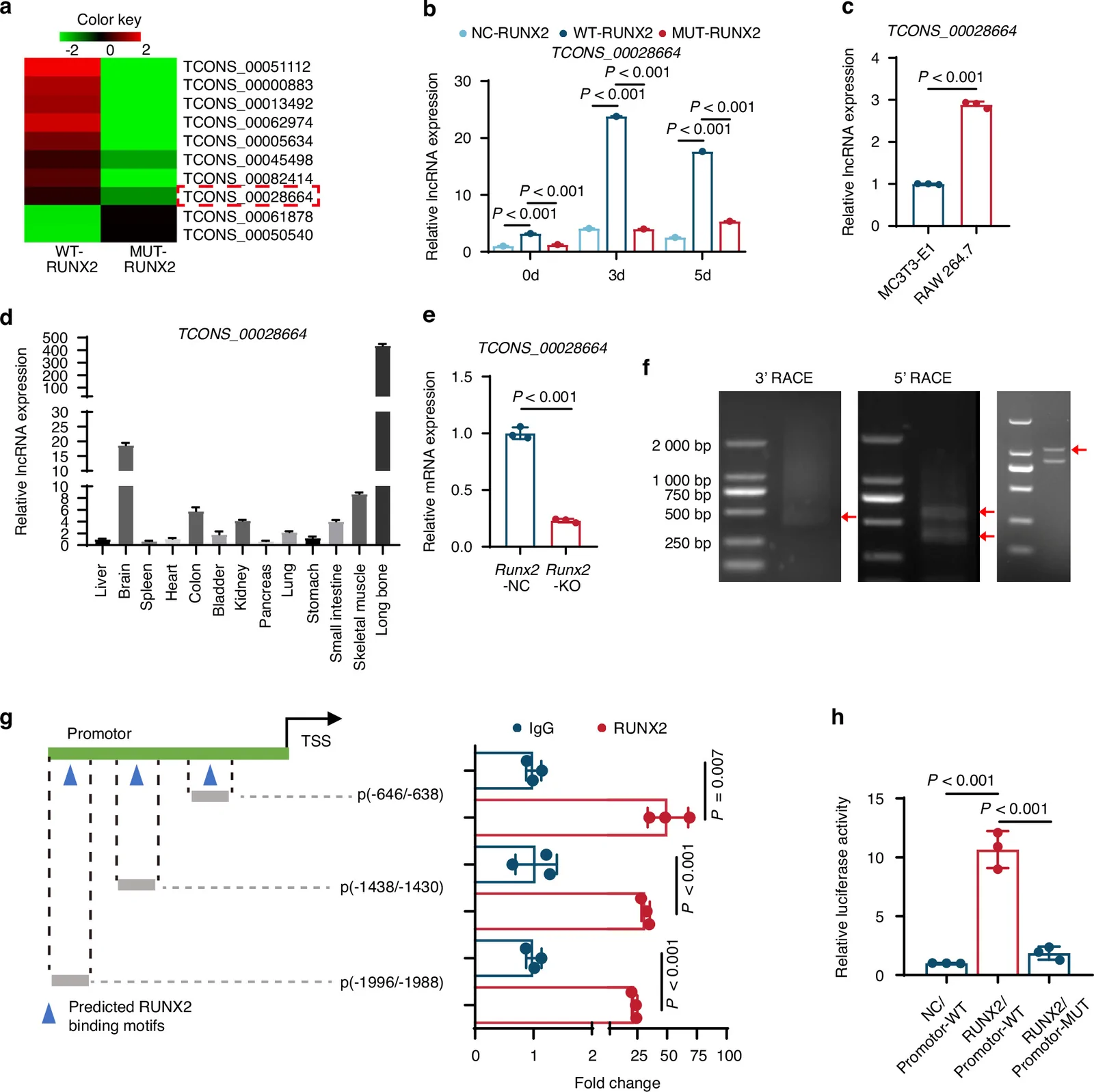
RUNX2 transcriptionally regulates *LncRNA-ROD* expression. **a** Heatmap of the top 10 differentially expressed lncRNAs between WT-RUNX2 and MUT-RUNX2 RAW 264.7 cells. Data source: PRJNA1158440. **b** RT-qPCR analysis of *LncRNA-ROD* expression between WT-RUNX2 and MUT-RUNX2 RAW 264.7 cells. RT-qPCR analysis of *LncRNA-ROD* expression levels in common cell lines (**c**) and mouse tissues (**d**). **e** RT-qPCR analysis of *LncRNA-ROD* expression following *Runx2* KO. **f** 5’ and 3’ rapid amplification of cDNA ends of *LncRNA-ROD*. **g** ChIP-qPCR confirming RUNX2 binding in the *LncRNA-ROD* promoter. **h** Dual-luciferase assays confirming that RUNX2 activates *LncRNA-ROD* transcription by binding to the promoter region. Data are presented as mean ± SD

LncRNAs have emerged as critical regulators of osteoclastogenesis and bone remodeling, highlighting their potential as therapeutic targets for bone metabolic diseases involving dysregulated osteoclast activity. Given RUNX2’s established role as a master transcription factor in osteoclast differentiation, we hypothesized that specific lncRNAs might modulate this process through direct interaction with RUNX2. To identify such lncRNAs, we performed RNA sequencing of RAW 264.7 cells overexpressing wild-type (WT) or Mutant RUNX2 variants (c.199 C > T, Q67X; c.514delT, 172 fs; c.674 G > T, R225L) during osteoclast induction (days 0 and 3). Differential expression analysis identified 10 lncRNAs with more than twofold expression changes between WT-RUNX2 and Mutant-RUNX2 groups (Fig. 4a), a finding confirmed by RT-qPCR (Fig. 4b, Fig. S3a). Among the lncRNAs upregulated during osteoclast differentiation, we further examined their expression profiles across multiple cell lines (Fig. 4c, Fig. S3b, d) and various mouse tissues (Fig. 4d, Fig. S3c, e). Among these candidates, *TCONS_00028664* exhibited high basal expression in osteoclast lineage cells and significant upregulation during osteoclast induction (Fig. 4b), with enriched expression specifically in RAW 264.7 cells (Fig. 4c) and murine long bones (Fig. 4d), indicating tissue-specific expression. We designated this transcript as *LncRNA-ROD* (*Regulator of Osteoclast Differentiation*) for further functional analysis.

*LncRNA-ROD* expression was significantly reduced in Mutant-RUNX2 cells compared to WT-RUNX2 cells (Fig. 4b) and in *Runx2*-KO cells compared to control (*Runx2*-NC) cells (Fig. 4e), indicating a positive regulatory relationship between RUNX2 and *LncRNA-ROD*. Full-length cDNA cloning using 3’and 5’ rapid amplification of cDNA ends (RACE) revealed *LncRNA-ROD* to be 1147 bp in length, consisting of three exons (Fig. 4f, Fig. S4a). Although it partially overlaps with the annotated gene *4833415N18Rik*, *LncRNA-ROD* represents a previously uncharacterized transcript. Coding potential analysis confirmed its noncoding nature (Fig. S4b).

Bioinformatic analysis using the JASPAR database identified three potential RUNX2 binding sites within the *LncRNA-ROD* promoter region (Table S2). Chromatin immunoprecipitation followed by quantitative PCR (ChIP-qPCR) confirmed direct binding of RUNX2 to all three sites (Fig. 4g), demonstrating that *LncRNA-ROD* is a direct transcriptional target of RUNX2. This was further validated by dual-luciferase reporter assays using wild-type and mutant promoter constructs, which confirmed RUNX2-dependent transcriptional activation of *LncRNA-ROD* (Fig. 4h). Notably, all RUNX2 mutants failed to activate *LncRNA-ROD* transcription (Fig. S5), indicating that functional RUNX2 is essential for this regulatory interaction.

To investigate the in vivo expression of *LncRNA-ROD* in osteoclasts and its co-localization with RUNX2, we performed dual-labeling experiments on P5 alveolar bone and 8-week-old femoral bone sections from wild-type mice. First, immunofluorescence staining for RUNX2 and the osteoclast marker CTSK revealed clear co-localization, confirming RUNX2 expression in mature osteoclasts in vivo (Fig. S6a, d). Second, *LncRNA-ROD* RNA FISH combined with CTSK immunofluorescence demonstrated that *LncRNA-ROD* is indeed expressed within osteoclasts (Fig. S6b, e). Notably, combined immunofluorescence and RNA FISH analysis showed that RUNX2 and *LncRNA-ROD* reside in the same cellular population (Fig. S6c, f), providing direct in vivo evidence for their association. These findings were consistently observed in both alveolar bone and long bone, suggesting that the RUNX2/*LncRNA-ROD* axis may regulate osteoclasts across distinct skeletal compartments.

**Fig. 5:**
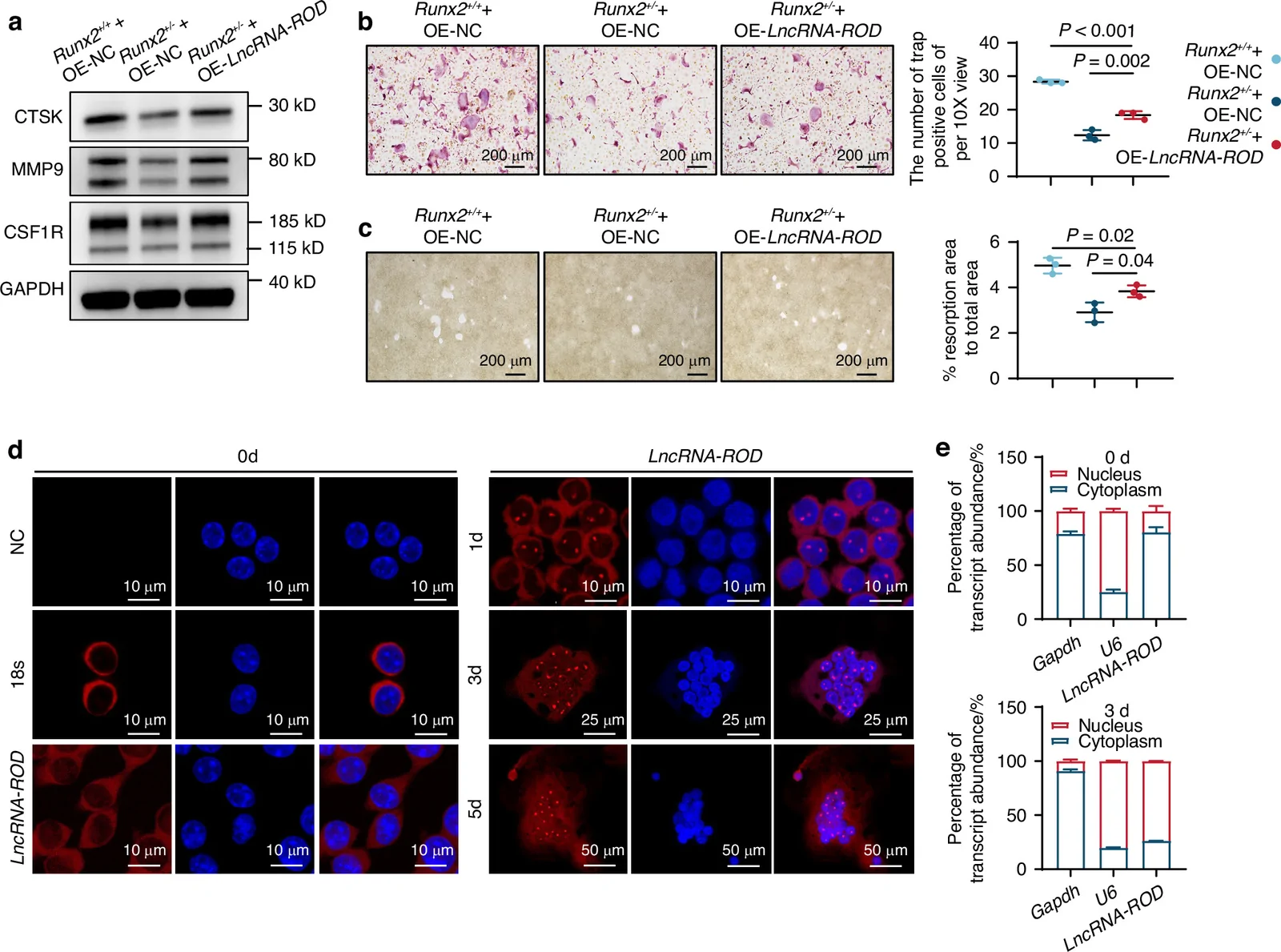
*LncRNA-ROD* mediates RUNX2-dependent osteoclastogenesis and undergoes dynamic nuclear translocation. **a** Western blot analysis of osteoclast-related proteins and CSF1R. Representative images of TRAP staining (**b**) and bone resorption pits (**c**) (*n* = 3). Subcellular localization of *LncRNA-ROD* during osteoclast differentiation detected by RNA FISH (**d**) and cytoplasmic-nuclear fractionation (**e**). Data are presented as mean ± SD

To determine whether *LncRNA-ROD* mediates RUNX2-dependent osteoclastogenesis, we overexpressed *LncRNA-ROD* in BMMs from *Runx2*^+/-^ mice. Following osteoclast induction, *LncRNA-ROD* overexpression significantly rescued impaired osteoclast differentiation caused by RUNX2 haploinsufficiency, as evidenced by restored expression of osteoclast-related markers and CSF1R at both mRNA (Fig. S6g) and protein levels (Fig. 5a, Fig. S6h), an increased number of TRAP-positive osteoclasts (Fig. 5b), and enlarged resorption pit areas (Fig. 5c). These results demonstrate that *LncRNA-ROD* is a critical downstream mediator of RUNX2, and that *LncRNA-ROD* overexpression is sufficient to restore osteoclast differentiation and bone resorption in RUNX2 haploinsufficient cells.

Subcellular localization was assessed using RNA fluorescence in situ hybridization (FISH) and cytoplasmic-nuclear fractionation assays, revealing that *LncRNA-ROD* is predominantly cytoplasmic under non-differentiating conditions but translocates to the nucleus during osteoclast differentiation (Fig. 5d, e).

In summary, we identified *LncRNA-ROD* as a novel, bone-enriched lncRNA transcriptionally activated by RUNX2. Rescue experiments identify it as a functional mediator of RUNX2-dependent osteoclastogenesis, and its dynamic nuclear translocation during osteoclast differentiation indicates that *LncRNA-ROD* may serve as a key effector in this process.

### *LncRNA-ROD* knockout enhances osteoclast fusion while suppressing bone resorption

**Fig. 6:**
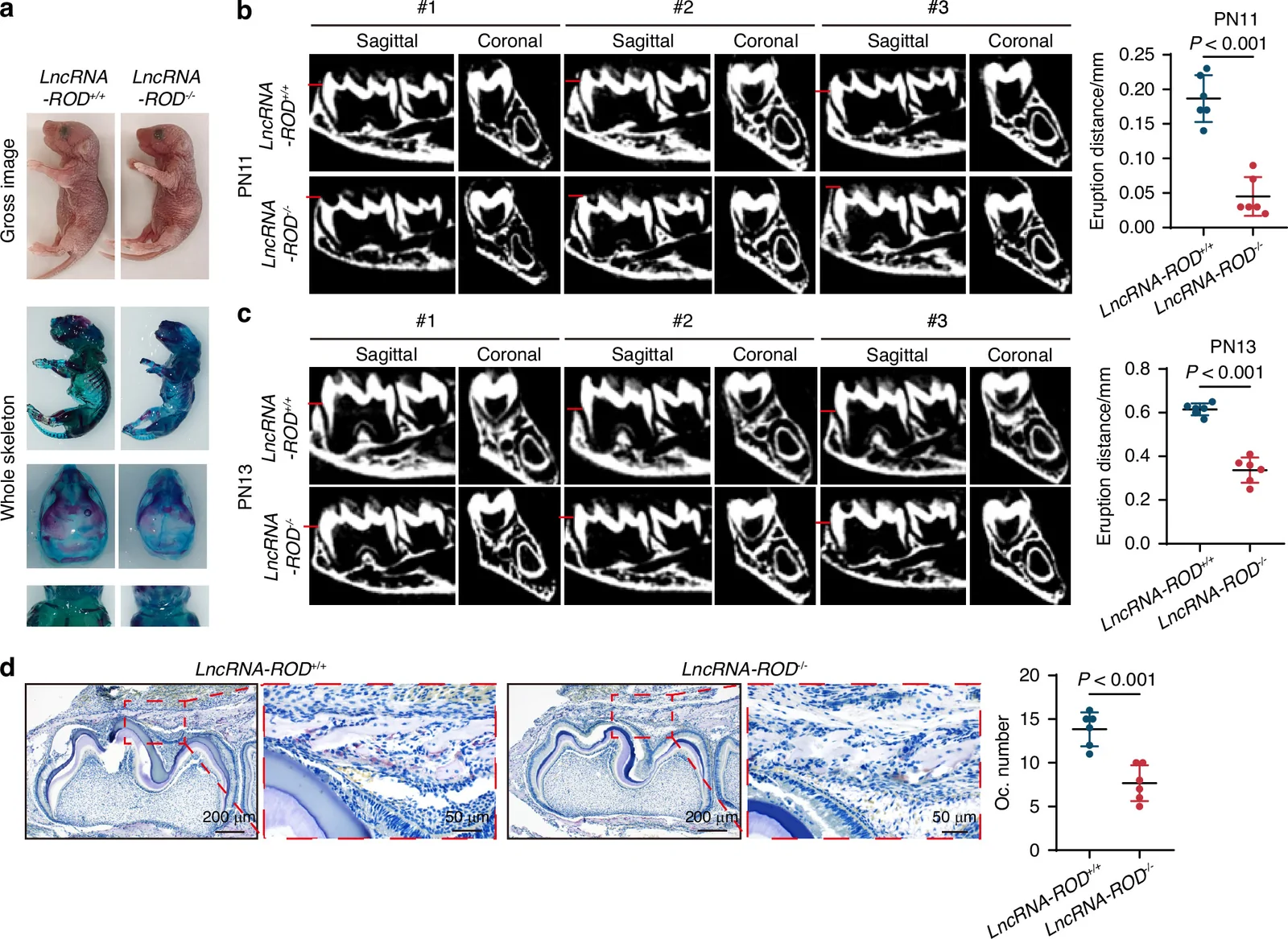
*LncRNA-ROD*^-/-^ mice exhibit a delayed tooth eruption phenotype. **a** Alizarin red-alcian blue staining of skeletal frameworks in *LncRNA-ROD*^-/-^ mice at PN0. Representative sagittal and coronal views. Eruption levels were quantified in the sagittal plane by measuring the vertical distance from the highest cusp tip to the highest point of the alveolar crest on the mesial side of the mandibular first molars in *LncRNA-ROD*^-/-^ mice at PN11 (*n* = 6) (**b**) and PN13 (*n* = 6) (**c**). Red lines indicate the highest point of the alveolar crest. The eruption level was measured as the vertical distance from this point to the highest cusp tip. **d** TRAP staining images and quantification of TRAP-positive osteoclasts in *LncRNA-ROD*^-/-^ mice at PN5 (*n* = 6). Data are presented as mean ± SD

Global *LncRNA-ROD* knockout mice were generated using CRISPR/Cas9. Skeletal analysis via Alizarin red-Alcian blue staining revealed classic CCD-like phenotypes in *LncRNA-ROD*^-/-^ mice, including delayed fontanelle closure and clavicular hypoplasia (Fig. 6a). Consistent with the *Runx2*^*+/-*^ mice phenotype, *LncRNA-ROD*^*-/-*^ mice exhibited delayed first molar eruption at PN11 and PN13 (Fig. 6b, c) with reduced TRAP-positive osteoclasts in alveolar bone adjacent to the tooth crown at PN5 (Fig. 6d). These findings support a role for *LncRNA-ROD*, like RUNX2, in alveolar bone remodeling associated with tooth eruption, as its deficiency impairs osteoclast differentiation and alveolar bone resorption.

Consistent with the observations in *Runx2*^+/-^ mice, we next examined mandibular molars in *LncRNA-ROD*^-/-^ mice at PN21. M1 and M2 showed a small but statistically significant reduction in eruption height compared with wild-type mice; however, 3D reconstruction revealed that the eruption heights were largely comparable between the two genotypes (Fig. S7). Notably, M3 had not yet achieved occlusion in either genotype, and the eruption height of M3 was markedly lower in *LncRNA-ROD*^-/-^ mice than in wild-type controls (Fig. S7). Together with the *Runx2*^+/-^ findings, these results indicate that the eruption delay observed at PN11–13 partially resolves for M1 and M2 by 3 weeks of age in both genetic models, whereas M3 remains substantially affected. Critically, this parallel temporal pattern supports the conclusion that RUNX2 and *LncRNA-ROD* function within a shared regulatory framework governing alveolar bone remodeling.

To dissect *LncRNA-ROD*’s role in osteoclastogenesis, we established *LncRNA-ROD*-overexpressing RAW 264.7 cells (OE-*LncRNA-ROD*). Unexpectedly, while OE-*LncRNA-ROD* cells showed elevated mRNA and protein levels of osteoclast markers (Fig. S8a, b), they exhibited reduced TRAP-positive osteoclasts numbers compared to controls (OE-NC) (Fig. S8c). Paradoxically, these cells demonstrated enhanced bone resorption activity, evidenced by larger resorption pit areas (Fig. S8d). This indicates that *LncRNA-ROD* overexpression uncouples osteoclast differentiation from resorption, suppressing maturation while promoting matrix degradation.

**Fig. 7:**
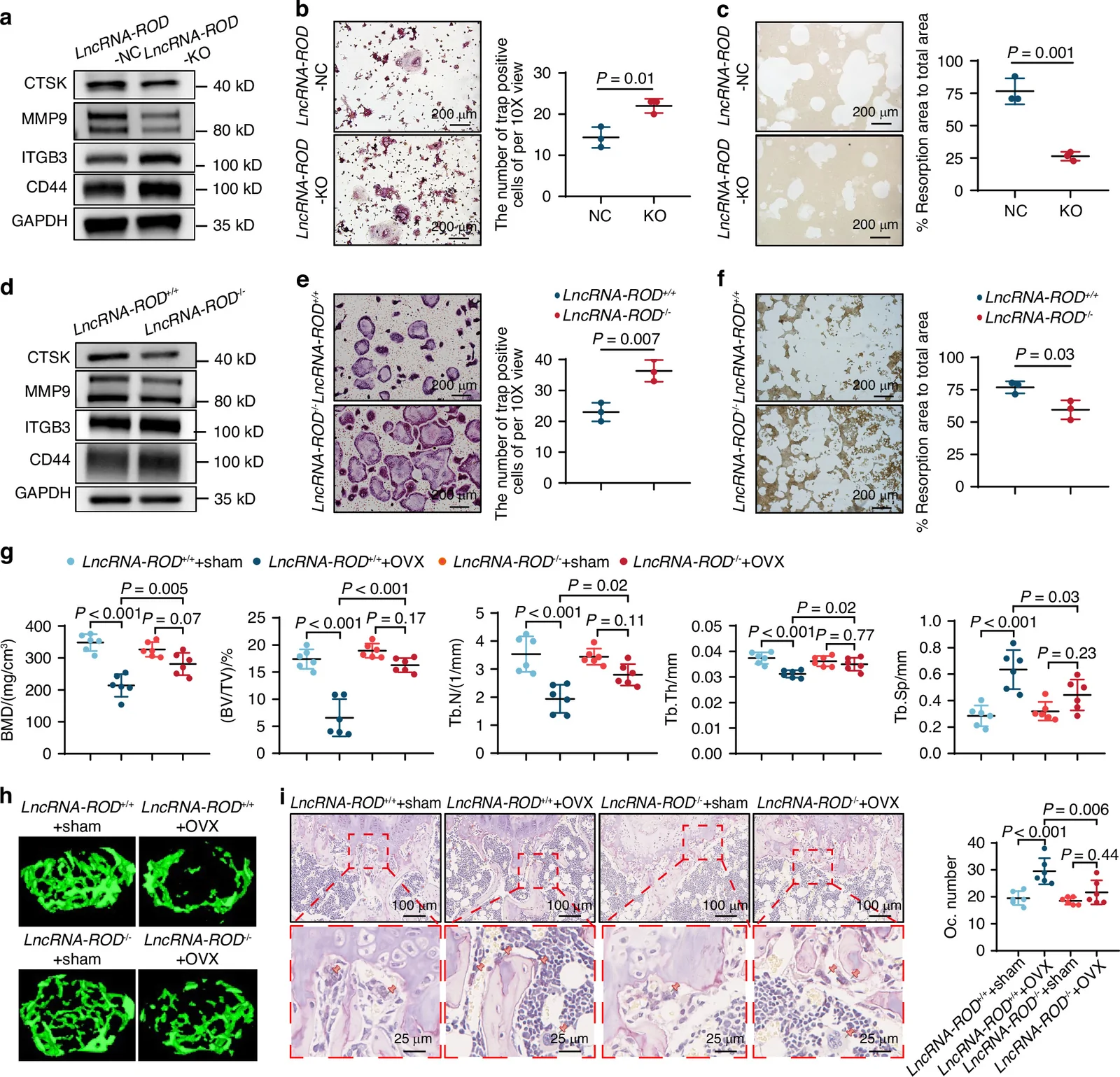
*LncRNA-ROD* knockout enhances osteoclast fusion but reduces bone resorption capacity. **a** Western blot analysis of osteoclast-related gene expression in *LncRNA-ROD* KO cells. Representative images of TRAP staining (**b**) and bone resorption pits (**c**) in *LncRNA-ROD* KO cells (*n* = 3). **d** Western blot analysis of osteoclast-related gene expression in *LncRNA-ROD*^-/-^ BMMs. Representative images of TRAP staining (**e**) and bone resorption pits (**f**) in *LncRNA-ROD*^-/-^ BMMs (*n* = 3). **g** Micro-CT analysis of trabecular bone parameters in the distal femur (*n* = 6). **h** Representative three-dimensional reconstruction images of the distal femur trabecular structure. **i** Representative images of TRAP staining in femoral sections (*n* = 6). Data are presented as mean ± SD

To validate this mechanism, *LncRNA-ROD* knockout RAW 264.7 cells (*LncRNA-ROD* KO) were generated using CRISPR/Cas9 (Fig. S9a), confirmed by Sanger sequencing (Fig. S9b) and RT-qPCR (Fig. S9c). During osteoclast differentiation, *LncRNA-ROD* KO cells exhibited three distinct features: (1) significantly reduced expression of the bone resorption markers CTSK and MMP9 (Fig. 7a, Fig. S9d); (2) increased TRAP-positive osteoclasts numbers (Fig. 7b); and (3) markedly diminished resorption pit areas (Fig. 7c). We proposed that *LncRNA-ROD* deletion enhances precursor fusion through upregulation of fusion-related genes (e.g., *Cd9*, *Cd44*, *Dc-stamp*, *Oc-stamp*, *Itgav*, *Itgb3*, *Atp6v0d2*; Fig. S9e), while concurrently suppressing expression of key bone resorption effectors, including CTSK and MMP9. This model is supported by elevated CD44 and ITGB3 protein levels in KO cells (Fig. 7a, Fig. S9d). Collectively, *LncRNA-ROD* acts as a critical regulatory node inversely coordinating osteoclast fusion and bone resorption, with deletion decoupling formation from functionality in isolated precursor cells.

Consistent with in vitro findings, BMMs from *LncRNA-ROD*^-/-^ mice showed increased CD44 and ITGB3 protein expression (Fig. 7d, Fig. S9f), elevated TRAP-positive osteoclast formation (Fig. 7e, Fig. S10), yet significantly impaired bone resorption capacity—evidenced by reduced CTSK/MMP9 expression (Fig. 7d, Fig. S9f) and smaller resorption pits (Fig. 7f). These results confirm *LncRNA-ROD* as a dual-function regulator: it inhibits fusion while being essential for resorptive function, such that its loss causes excessive fusion but defective matrix degradation.

For physiological relevance, we employed an OVX-induced osteoporosis model. The experimental grouping and treatments were consistent with those used in the *Runx2*^+/-^ mouse model (Fig. S11a). Eight weeks post-OVX, femurs from four groups (*LncRNA-ROD*^*+/+*^+sham, *LncRNA-ROD*^*+/+*^ + OVX, *LncRNA-ROD*^-/-^+sham, and *LncRNA-ROD*^-/-^ + OVX) underwent micro-CT analysis. *LncRNA-ROD*^-/-^ mice exhibited significant protection against estrogen deficiency-induced bone loss, with markedly improved bone parameters (increased BMD, BV/TV, Tb.N; decreased Tb.Sp) (Fig. 7g). Three-dimensional reconstruction and histological confirmed preserved trabecular architecture in in *LncRNA-ROD*^-/-^ + OVX mice versus wild-type OVX controls (Fig. 7h, Fig. S11b). TRAP staining revealed significantly fewer osteoclasts in *LncRNA-ROD*^-/-^ + OVX mice (Fig. 7i). Thus, genetic deletion of *LncRNA-ROD* confers resistance to OVX-induced osteoporosis through dual suppression of osteoclast differentiation and resorptive activity.

To further examine the molecular basis of impaired bone resorption in the OVX model, we performed immunofluorescence staining for CTSK and MMP9 on femoral sections from the four OVX groups. Notably, the *LncRNA-ROD*^−/−^ + OVX group displayed markedly reduced fluorescence intensity for both CTSK and MMP9 compared to the *LncRNA-ROD*^+/+^ + OVX group (Fig. S11c, d). These findings are consistent with the reduced osteoclast numbers and preserved bone mass observed via micro-CT and TRAP staining, indicating suppressed resorptive capacity in vivo.

### *LncRNA-ROD* sequesters QKI to regulate osteoclast differentiation and bone resorption

**Fig. 8:**
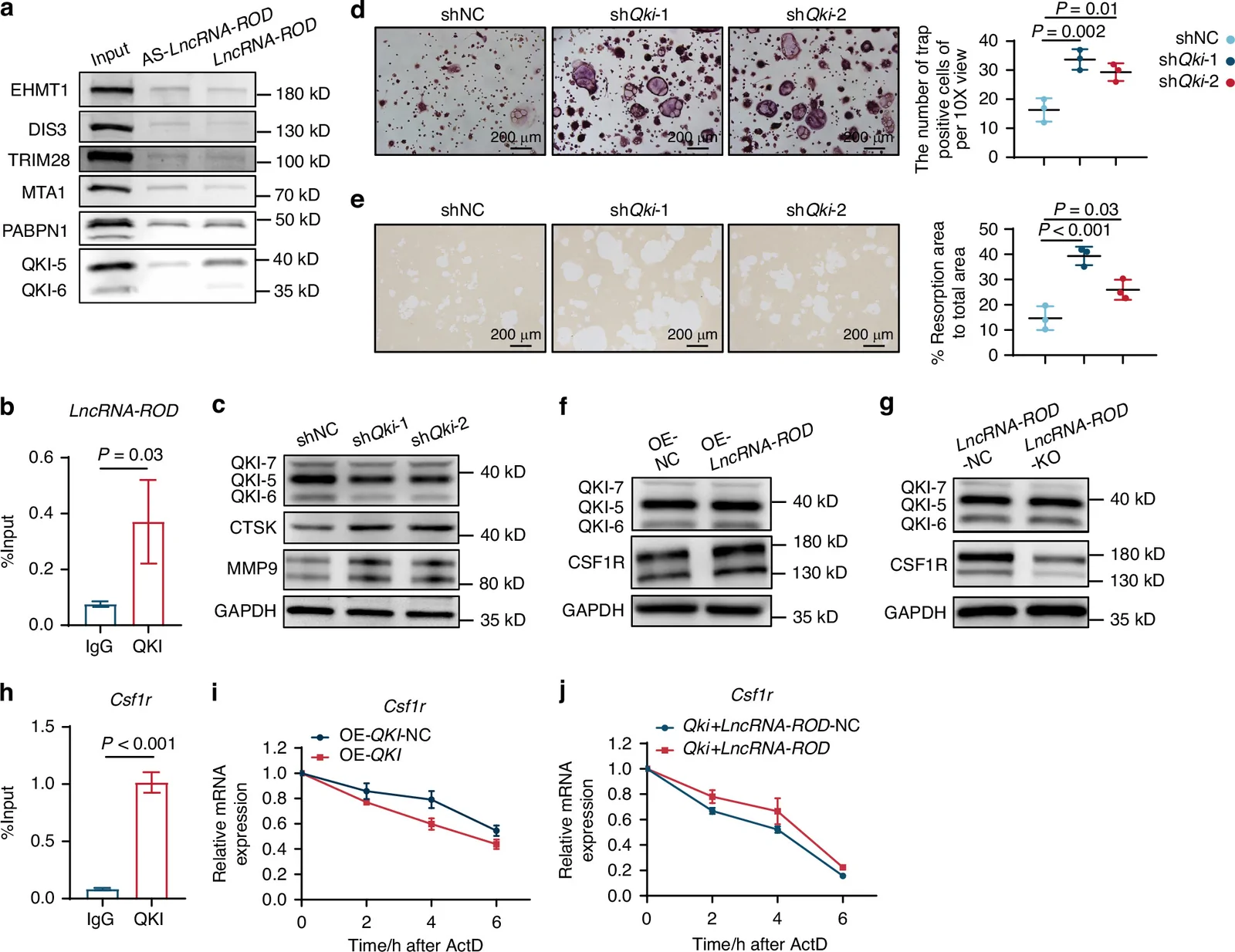
*LncRNA-ROD* interacts with QKI to regulate osteoclast differentiation and bone resorption. **a** Western blot analysis showing the interaction between *LncRNA-ROD* and QKI. **b** RIP-qPCR confirming the binding of QKI to *LncRNA-ROD*. **c** Western blot analysis of osteoclast-related gene expression in sh*Qki* cells. Representative images of TRAP staining (**d**) and bone resorption pits (**e**) in sh*Qki* cells (*n* = 3). Western blot analysis of QKI and CSF1R expression following *LncRNA-ROD* overexpression (**f**) or knockout (**g**). **h** RIP-qPCR confirming the binding of QKI to *Csf1r* mRNA. **i** RNA stability assay showing increased degradation rate of *Csf1r* mRNA upon QKI overexpression. **j** RNA stability assay showing that *LncRNA-ROD* overexpression partially rescues the stability of *Csf1r* mRNA reduced by QKI overexpression. Data are presented as mean ± SD

The nuclear translocation of *LncRNA-ROD* during osteoclast differentiation suggested a potential regulatory role in the nucleus. To elucidate the underlying mechanism, we performed RNA pulldown followed by mass spectrometry to identify *LncRNA-ROD*-interacting proteins. Among 15 proteins enriched in the *LncRNA-ROD* pulldown (relative to antisense controls, *LncRNA-ROD*:antisense ratio ≥ 2; Table S3), RNA-binding protein QKI was confirmed as a direct interactor using Western blot (Fig. 8a, Fig. S12a) and RNA immunoprecipitation followed by quantitative PCR (RIP-qPCR) (Fig. 8b). Immunofluorescence further demonstrated nuclear colocalization of QKI and *LncRNA-ROD* during differentiation (Fig. S12b), supporting their functional interaction.

To investigate the role of QKI in osteoclastogenesis, we generated QKI-knockdown RAW 264.7 cells (sh*Qki*). Compared with control cells (sh*Qki*-NC), sh*Qki* cells exhibited significantly elevated expression of osteoclast-related genes at both the mRNA (Fig. S12c) and protein levels (Fig. 8c, Fig. S12d). These cells also formed more TRAP-positive osteoclasts (Fig. 8d) and larger resorption pits (Fig. 8e), indicating that QKI acts as a negative regulator of osteoclast differentiation and bone resorption, with *LncRNA-ROD* likely antagonizing QKI activity.

Previous studies have established that QKI binds to the 3’ untranslated region of *Csf1r* mRNA, promoting its degradation.^23,24^ To determine whether *LncRNA-ROD* modulates this pathway, we assessed QKI and CSF1R protein expression following *LncRNA-ROD* overexpression or knockout. While QKI protein levels remained unchanged, CSF1R expression increased upon *LncRNA-ROD* overexpression (Fig. 8f, Fig. S12e) and decreased following its knockout (Fig. 8g, Fig. S12f), suggesting that *LncRNA-ROD* interferes with QKI-mediated *Csf1r* mRNA degradation without altering QKI expression. RIP-qPCR confirmed QKI binding to *Csf1r* mRNA (Fig. 8h), and RNA stability assays revealed that QKI overexpression reduced *Csf1r* mRNA stability (Fig. 8i). Critically, co-overexpression of *LncRNA-ROD* rescued the QKI-induced destabilization of *Csf1r* mRNA (Fig. 8j), demonstrating that *LncRNA-ROD* competitively inhibits QKI-mediated mRNA degradation.

Having established that *LncRNA-ROD* stabilizes *Csf1r* mRNA by sequestering QKI, we next investigated whether *LncRNA-ROD* deletion affects CSF1R protein expression in vivo under pathological conditions. Immunofluorescence staining for CSF1R was performed on femoral sections from the four experimental groups (*LncRNA-ROD*^+/+^ + sham, *LncRNA-ROD*^+/+^ + OVX, *LncRNA-ROD*^-/-^ + sham, and *LncRNA-ROD*^-/-^ + OVX). Notably, the *LncRNA-ROD*^-/-^ + OVX group exhibited markedly reduced CSF1R fluorescence intensity compared to the *LncRNA-ROD*^+/+^ + OVX group (Fig. S13). These results align with the reduced osteoclast numbers and preserved bone mass observed in the OVX model (Fig. S11c, d), providing tissue-level evidence that *LncRNA-ROD* deletion impairs the CSF1R-mediated osteoclast differentiation axis in vivo. Collectively, these data suggest that *LncRNA-ROD* knockout compromises osteoclast differentiation and resorptive capacity in vivo, primarily by disrupting the CSF1R-mediated precursor recruitment axis.

All three QKI isoforms (QKI-5, QKI-6, and QKI-7) are detectable, with QKI-5 being the most abundant (Fig. 8c, f, g). This expression profile indicates that QKI-5 is the most abundantly expressed isoform in our system.

Given that QKI-5 is the predominant isoform, we next determined whether it is the principal mediator of the pan-QKI knockdown phenotype. We designed an isoform-specific siRNA targeting *Qki-5* (si-*Qki-5*). Western blot confirmed efficient and specific knockdown of QKI-5 without affecting QKI-6 or QKI-7 expression (Fig. S14a, b). Following osteoclast induction, si-*Qki*-5 cells exhibited marked upregulation of osteoclast-related genes, a significant increase in TRAP-positive osteoclasts, and enlarged resorption pit areas (Fig. S14c, d). These phenotypes closely mirrored those observed with pan-QKI knockdown (sh*Qki*; Fig. 8c–e), suggesting that QKI-5 is the dominant isoform negatively regulating osteoclast differentiation and bone resorption, and that the pan-QKI knockdown phenotype is primarily attributable to QKI-5 abrogation.

To dissect which QKI isoforms interact with *LncRNA-ROD* and *Csf1r* mRNA, we performed RIP-qPCR using isoform-specific antibodies against QKI-5, QKI-6, and QKI-7. Both QKI-5 and QKI-6 significantly enriched *LncRNA-ROD* and *Csf1r* mRNA, whereas no specific binding was detected for QKI-7 (Fig. S14e, f). Given the established subcellular localization of QKI-5 (predominantly nuclear) and QKI-6 (predominantly cytoplasmic), these results suggest that *LncRNA-ROD* engages QKI-5 in the nucleus and QKI-6 in the cytoplasm. This spatial pattern supports a dual-compartment cooperative model: *LncRNA-ROD* sequesters nuclear QKI-5 to limit its shuttling to the cytoplasm, where it would otherwise negatively regulate *Csf1r* mRNA stability, while simultaneously engaging cytoplasmic QKI-6 to potentially inhibit its degradation of *Csf1r* mRNA.

#### QKI knockdown reverses osteoclast differentiation defects in RUNX2 or *LncRNA-ROD* deficiency

To assess the in vivo functional role of the RUNX2/*LncRNA-ROD*/QKI axis, we systemically delivered AAV-sh*Qki* via tail vein injection in *Runx2*^+/-^ mice or *LncRNA-ROD*^-/-^ mice subjected to sham or OVX surgery (Fig. S15a, b). Two weeks post-injection, EGFP imaging confirmed widespread AAV distribution with strong liver expression and detectable bone signal (Fig. S16). QKI knockdown in femoral tissue was validated by immunohistochemistry (Fig. S17a, b).

**Fig. 9:**
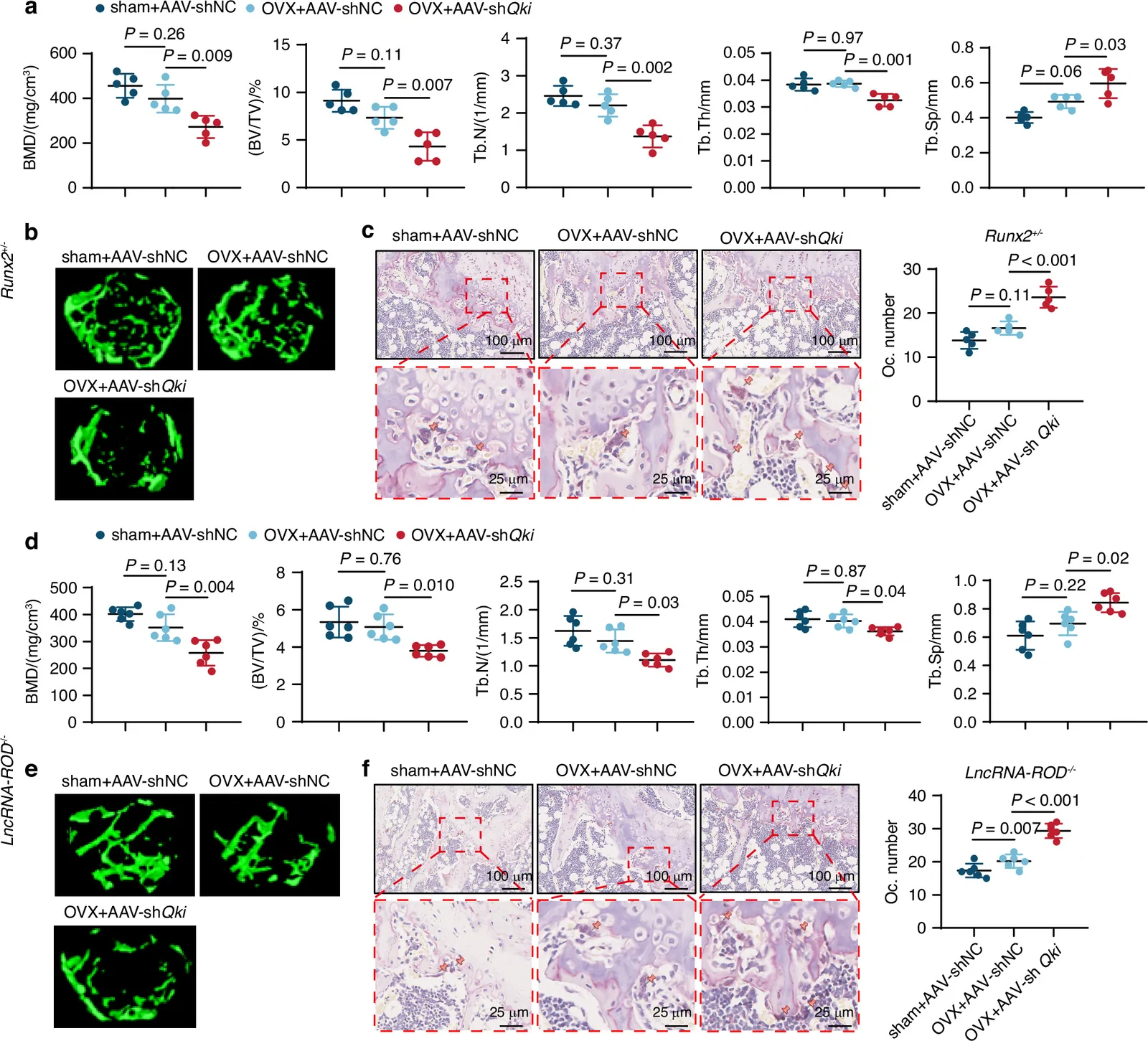
Intravenous injection of AAV-sh*Qki* partially rescues osteoclast differentiation defects caused by RUNX2 or *LncRNA-ROD* knockout. **a**–**c** *Runx2*^+/-^ mice. **d**–**f** *LncRNA-ROD*^-/-^ mice. **a**,** d** Micro-CT analysis of trabecular bone parameters in the distal femur (*n* = 5 and 6, respectively). **b**,** e** Representative three-dimensional reconstruction images of the distal femur trabecular structure. **c**,** f** Representative images of TRAP staining in femoral sections (*n* = 5 and 6, respectively). Data are presented as mean ± SD

Micro-CT analysis revealed that AAV-sh*Qki* treatment enhanced osteoclast-mediated bone resorption in *Runx2*^+/-^+OVX mice, evidenced by reduced trabecular bone mass (BMD, BV/TV, Tb.Th, and Tb.N decreased; Tb.Sp increased; Fig. 9a), deteriorated trabecular microarchitecture (Fig. 9b, Fig. S18a), and increased TRAP-positive osteoclast numbers (Fig. 9c). Critically, these data demonstrate that QKI knockdown restores osteoclast differentiation capacity impaired by RUNX2 deficiency.

Similarly, in *LncRNA-ROD*^-/-^ + OVX mice, AAV-sh*Qki* treatment exacerbated osteoclast differentiation and bone resorption (Fig. 9d–f, Fig. S18b), confirming that QKI suppression promotes osteoclast activity downstream of *LncRNA-ROD*. Together, these data demonstrate that RUNX2 promotes osteoclast differentiation and bone resorption by transcriptionally activating *LncRNA-ROD* and inhibiting QKI-mediated mRNA degradation.

## Discussion

**Fig. 10:**
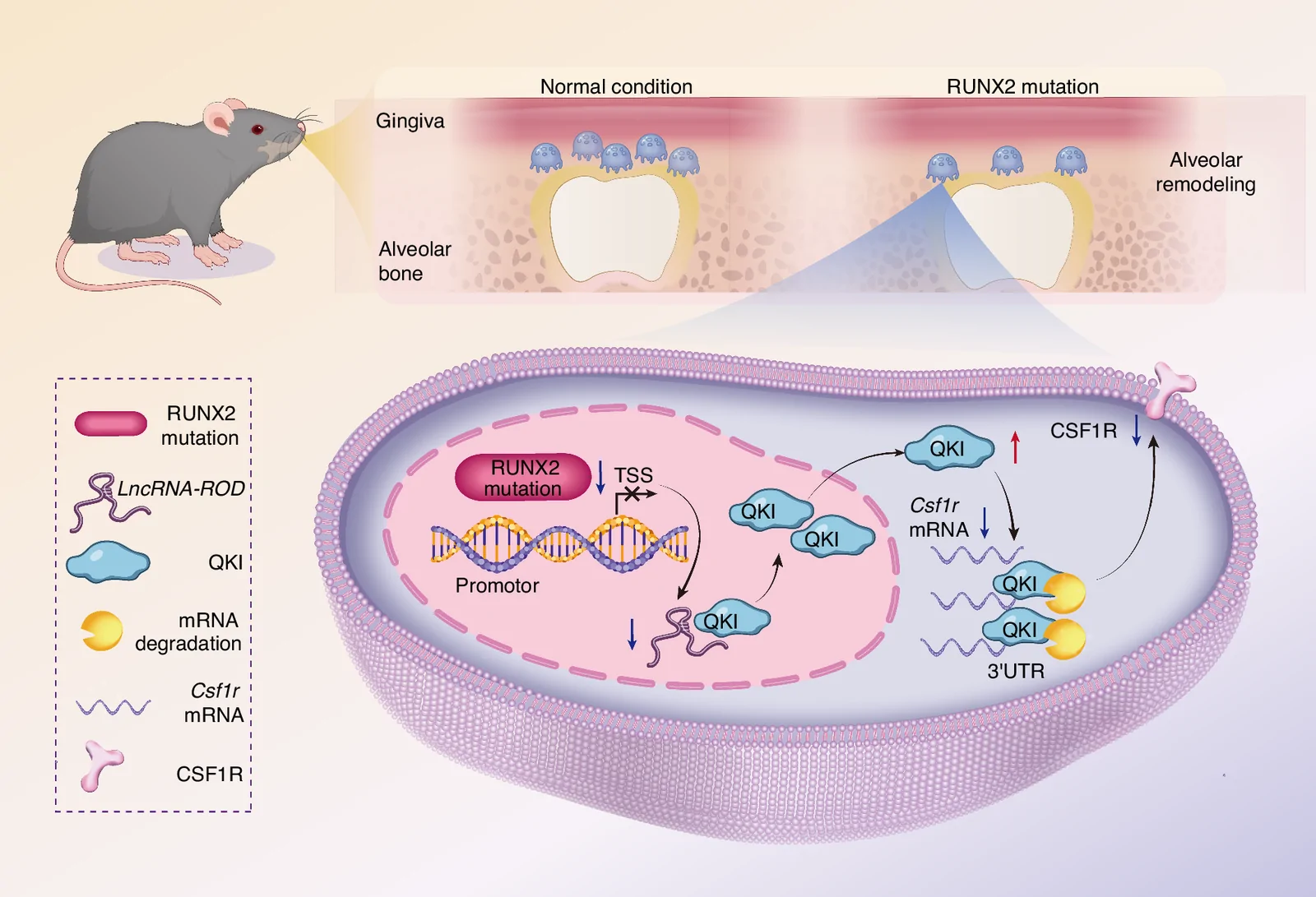
Model for the regulation of osteoclastogenesis by the RUNX2/*LncRNA-ROD*/QKI axis. In the presence of normal RUNX2, *LncRNA-ROD* expression is upregulated, sequestering QKI and preventing its interaction with *Csf1r* mRNA, thereby maintaining CSF1R expression and promoting osteoclast differentiation and bone resorption. When RUNX2 is mutated or knocked out, *LncRNA-ROD* expression is downregulated, releasing QKI, which binds to and degrades *Csf1r* mRNA, inhibiting osteoclast differentiation and bone resorption

The molecular mechanisms underlying delayed tooth eruption in CCD caused by RUNX2 mutation or haploinsufficiency remain poorly defined. This study identifies a critical RUNX2/*LncRNA-ROD*/QKI axis that directly promotes osteoclast differentiation and supports alveolar bone remodeling. Our findings demonstrate that RUNX2 transcriptionally activates *LncRNA-ROD*, which stabilizes *Csf1r* mRNA by sequestering QKI-5 in the nucleus and QKI-6 in the cytoplasm, thereby cooperatively promoting osteoclastogenesis (Fig. 10). Both *Runx2*^+/-^ and *LncRNA-ROD*^-/-^ mice exhibit impaired alveolar bone resorption and delayed mandibular molar eruption—phenotypes consistent with CCD. This observation aligns with previously published literature.^11,25,26^ Mechanistically, QKI knockdown rescues osteoclast differentiation defects in both genetic models, validating the functional regulatory cascade of this axis. These findings support a mechanistic link between RUNX2 dysfunction, impaired alveolar bone remodeling, and delayed tooth eruption in CCD, highlighting *LncRNA-ROD* as a potential therapeutic target for bone metabolic disorders.

Consistent with our prior work demonstrating that RUNX2 promotes osteoclast differentiation and bone resorption through regulation of the AKT/NFATc1/CTSK axis,^14^ this study provides further evidence for the direct regulation of osteoclastogenesis by RUNX2. Critically, dual-labeling experiments confirmed that RUNX2 is expressed in CTSK-positive osteoclasts within both alveolar and femoral bone tissue, with *LncRNA-ROD* co-expressed in these same cells. These findings establish that the proposed transcriptional axis operates in osteoclasts in vivo. Furthermore, CRISPR/Cas9-generated *Runx2* KO RAW 264.7 cells and *Runx2*^+/−^ mice both exhibit significant impairments in osteoclast differentiation and bone resorption, thereby distinguishing the cell-autonomous effects of RUNX2 from its previously reported indirect modulation via the RANKL/OPG signaling pathway.^9–11^ Collectively, our study highlights that RUNX2 not only indirectly influences osteoclast activity but also directly drives these processes, reinforcing its central role in bone metabolism.

*LncRNA-ROD* exerts distinct, context-dependent functions within the osteoclast lineage. In vitro, its deletion in RAW 264.7 cells or BMMs triggers a hyper-fusion phenotype driven by upregulated CD44 and ITGB3. Although this increases the number of TRAP-positive osteoclasts, these osteoclasts exhibit impaired resorptive capacity due to suppressed CTSK and MMP9 expression. In contrast, in vivo *LncRNA-ROD* knockout leads to a marked reduction in osteoclast numbers in both alveolar and femoral bone. Mechanistically, *LncRNA-ROD* stabilizes *Csf1r* mRNA by sequestering QKI, thereby maintaining CSF1R expression essential for osteoclast precursor recruitment and survival. Consistent with this mechanism, immunofluorescence analysis of femoral sections from OVX mice revealed markedly reduced CTSK, MMP9, and CSF1R signals in *LncRNA-ROD*-deficient mice compared with controls. Collectively, these findings lead us to speculate that *LncRNA-ROD* knockout compromises osteoclast differentiation and resorptive capacity in vivo primarily by disrupting the CSF1R-mediated precursor recruitment axis. Thus, the RUNX2/*LncRNA-ROD*/QKI/CSF1R axis provides a molecular basis for this phenotypic divergence, highlighting how the bone microenvironment dictates the functional outcome of *LncRNA-ROD* deficiency.

LncRNAs regulate gene expression and cellular functions at the transcriptional and post-transcriptional levels, including influencing genomic stability and mRNA stability, affecting translation efficiency, acting as molecular sponges, and serving as structural components.^27–31^ In this study, we discovered that *LncRNA-ROD* binds to QKI, a member of the signal transduction and activation of RNA family involved in various physiological and pathological processes by modulating mRNA splicing, stability, and translation efficiency.^32–34^ Dysregulation of QKI has been linked to several diseases, including neurodegenerative disorders, cancer, and metabolic diseases.^35–37^ Notably, isoform-specific RIP revealed that QKI-5 and QKI-6, but not QKI-7, bind both *LncRNA-ROD* and *Csf1r* mRNA. Given the established subcellular localization of QKI-5 (nuclear) and QKI-6 (cytoplasmic), these data support a dual-compartment cooperative model: *LncRNA-ROD* sequesters nuclear QKI-5 to limit its shuttling to the cytoplasm, where it would otherwise negatively regulate *Csf1r* mRNA stability, while simultaneously engaging cytoplasmic QKI-6 to potentially inhibit its degradation of *Csf1r* mRNA. By sequestering both isoforms from accessing the 3′UTR of *Csf1r* mRNA, *LncRNA-ROD* protects the transcript from degradation and upregulates CSF1R to promote osteoclast differentiation. This model reconciles the nuclear translocation of *LncRNA-ROD* during osteoclast differentiation with the predominantly cytoplasmic regulation of mRNA stability. This expands the repertoire of lncRNA-mediated post-transcriptional regulation in bone biology, aligning with emerging paradigms of lncRNA-mRNA-protein ternary interactions in disease pathogenesis.

A seeming discrepancy exists between the reduced long bone BMD in *Runx2*^+/-^ mice and the relatively preserved alveolar bone density in our CCD patient. These observations are not contradictory; rather, they reflect distinct physiological processes across skeletal compartments. In long bones and lumbar vertebrae, which develop through endochondral ossification, bone mass is primarily maintained by osteoblast-mediated formation. RUNX2 haploinsufficiency impairs osteoblast differentiation, resulting in decreased BMD and trabecular bone volume in murine models,^38,39^ consistent with our findings in the OVX model. Similar osteopenic phenotypes have been reported in CCD patients.^40,41^ In the coronal alveolar bone surrounding the tooth crown, however, physiological eruption requires active osteoclast-mediated bone resorption. The absence of TRAP-positive osteoclasts in the CCD patient and the reduced osteoclast numbers in *Runx2*^+/-^ mice indicate that resorption is impaired, leading to local bone accumulation that manifests as increased density on micro-CT. Yoda et al. reported that BV/TV in the eruption pathway of *Runx2*^+/-^ mice was significantly higher than in wild-type controls, directly supporting this interpretation.^26^ The tissue-specific enrichment of *LncRNA-ROD* in osteoclast precursors and its role in promoting osteoclast differentiation through the QKI-5/*Csf1r* axis provide a potential molecular basis for this differential skeletal response. Collectively, these findings underscore that RUNX2 haploinsufficiency exerts distinct effects on bone formation and resorption depending on the skeletal site and physiological process.

Several limitations of this study should be acknowledged. First, the absence of direct evidence demonstrating restoration of alveolar bone remodeling or tooth eruption represents a key limitation; consequently, our data support a role for the RUNX2*/LncRNA-ROD*/QKI-5 axis in alveolar bone remodeling rather than establishing direct causality for tooth eruption itself. Second, the use of global genetic models precludes a definitive attribution of the observed phenotypes to cell-autonomous functions in osteoclasts. Because RUNX2 is also critically expressed in osteoblasts, the *Runx2*^+/-^ model cannot resolve cell-type-specific contributions, and the delayed eruption and impaired bone resorption likely reflect complex interactions among multiple cell types, including interactions between osteoblasts and osteoclasts. While *Runx2*^+/-^ mice closely mirror the clinical presentation of CCD caused by RUNX2 haploinsufficiency, our conclusions regarding RUNX2 function in osteoclasts are primarily supported by in vitro loss-of-function data in RAW 264.7 cells and BMMs, whereas the in vivo findings should be interpreted as reflecting the overall pathological consequence of systemic RUNX2 deficiency. Similarly, the global *LncRNA-ROD* knockout model cannot exclude contributions from non-osteoclast lineages or systemic compensatory effects; although *LncRNA-ROD* exhibits enriched expression in bone tissue and higher levels in osteoclast precursors than in osteoblast precursors, and our in vitro CRISPR/Cas9-mediated knockout in RAW 264.7 cells demonstrated impaired bone resorption capacity, these findings do not definitively establish a cell-autonomous role within the complex bone microenvironment in vivo. Future studies should therefore prioritize targeted rescue experiments in alveolar bone, including osteoclast-specific or alveolar-targeted delivery systems, as well as osteoclast-specific conditional knockout strategies for both RUNX2 and *LncRNA-ROD*, to further validate the role of this axis during tooth eruption and to clarify the direct cell-autonomous contributions of each component to osteoclast-mediated alveolar bone resorption. Another limitation concerns the interpretation of the OVX data. The preserved bone volume in ovariectomized *Runx2*^+/-^ mice could theoretically reflect either impaired osteoclast-mediated bone resorption or enhanced osteogenesis. Although our TRAP staining and in vitro osteoclast differentiation data support the former, we did not directly assess bone formation in this model. Future studies evaluating osteoblast differentiation and bone formation would help definitively exclude the latter possibility.

From a translational perspective, lncRNAs have emerged as attractive therapeutic targets. Antisense oligonucleotides targeting lncRNAs have demonstrated efficacy in preclinical models of multiple myeloma,^42,43^ and small-molecule inhibitors of lncRNAs have shown promise in regulating glucose metabolism in diabetes.^44^ Emerging strategies targeting conserved structural motifs within lncRNAs may improve treatment efficacy while minimizing immunogenicity.^45^ The aforementioned studies indicate the necessity of further research aimed at identifying and validating the functional motifs of *LncRNA-ROD*, which could pave the way for *LncRNA-ROD* to serve as a promising therapeutic target for bone metabolic disorders such as delayed tooth eruption and osteoporosis. Additionally, the role of *LncRNA-ROD* within the broader bone microenvironment needs further investigation. Generating osteoclast-specific RUNX2 or *LncRNA-ROD* knockout models will be critical for examining their direct effects in osteoclasts, providing more precise insights into their roles in osteoclast differentiation.

## Materials and methods

### Ethics approval and consent to participate

The human study was approved by the Ethical Committee of Peking University School of Stomatology (approval number: PKUSSIRB-2012004), and written informed consent was obtained from all participants. The study was conducted in accordance with the Declaration of Helsinki and applicable local ethical guidelines.

All animal research was approved by the Peking University Animal Ethics Committee (approval number: PUIRB-LA2023149), and all procedures involving animals were performed in accordance with the relevant guidelines, specifically the National Institutes of Health Guide for the Care and Use of Laboratory Animals.

### Isolation of bone marrow-derived monocytes/macrophages (BMMs)

Six- to eight-week-old male C57BL/6 wild-type mice were purchased from Vital River Laboratories (Beijing, China) and housed in a specific pathogen-free environment with a temperature of 22–24 °C and humidity of 40%–60%. The mice were euthanized by cervical dislocation, followed by sterilization with 75% ethanol. Femurs and tibiae were isolated, cleaned of surface soft tissues, and placed in PBS. The bones were rinsed with sterile PBS 4–5 times, and approximately 1-mm sections were removed from both ends of the femur and tibia using sterile forceps. Bone marrow was flushed from the cavities with a syringe filled with α-MEM until the marrow cavities appeared white. The collected cells were treated with red blood cell lysis buffer (Solarbio, R1010, Beijing, China), and after lysis, the cells were resuspended in α-MEM supplemented with 10% FBS and 1% penicillin/streptomycin. The cells were cultured overnight in a 10 cm culture dish. Nonadherent cells were collected the following day as osteoclast precursors for osteoclast differentiation.

### Osteoclast differentiation

RAW 264.7 cells were seeded in 24-well or 12-well plates at a density of 3.5 × 10^4^ cells per mL. To induce osteoclast differentiation, 10 ng/mL recombinant mouse RANKL (R&D Systems, 462-TEC-010, Minneapolis, MN, USA) was added to the medium, and the cells were incubated for 3–6 days, with the medium changed every two days.

BMMs were collected and centrifuged at 112 × *g* for 5 min. The supernatant was discarded, and the cells were resuspended in 4 mL of complete medium containing 30 ng/mL recombinant mouse M-CSF (BioLegend, 576404, San Diego, CA, USA) and 50 ng/mL recombinant mouse RANKL. The cell suspension was then seeded in 24-well or 12-well plates at a density of 5 × 10^6^ cellspermL and cultured for 4–7 days, with medium changes every 2 days.

### TRAP staining

After 3–4 days of osteoclast induction, RAW 264.7 cells or BMMs were fixed with 4% paraformaldehyde (Solarbio, P1110) at room temperature for 5 min. The TRAP staining solution was prepared according to the manufacturer’s instructions (Leukocyte Acid Phosphatase (TRAP) Kit, Sigma-Aldrich, 387 A, St. Louis, MO, USA). Cells were incubated with the TRAP staining solution at 37 °C for 40 min, followed by three washes with PBS. Five pre-defined fields of view (top, middle, bottom, left, and right) were captured per well under a light microscope (Olympus, IX53, Tokyo, Japan), and the number of TRAP-positive multinucleated cells ( ≥ 3 nuclei) was counted. The mean value per well was used for statistical analysis.

### Bone resorption assay

RAW 264.7 cells or BMMs were seeded in bone resorption plates (Cosmo Bio, CSR-BRA-24P, Tokyo, Japan). After 6–7 days of osteoclast induction, the culture medium was discarded, and the cells were removed by soaking in 10% NaClO (Aladdin, S101636, Shanghai, China) for 10 min. Wells were washed with PBS and air-dried. Five pre-defined fields of view (top, middle, bottom, left, and right) per well were imaged under a light microscope (Olympus, IX53). The resorbed area was quantified using ImageJ software (National Institutes of Health, USA), and the mean percentage of the resorbed area relative to the total culture area per well was calculated.

### RNA-seq and bioinformatics analysis

RAW 264.7 cells overexpressing WT-RUNX2 or MUT-RUNX2 were harvested at 0 and 3 days post-induction for RNA extraction. RNA samples were sent to Berry Genomics (Beijing, China) for library construction and RNA sequencing with the Illumina HiSeq 2000 platform (Illumina, USA). Differentially expressed genes were identified based on the criteria | log_2_(fold change) | > 1 and *P* < 0.05, and were subsequently analyzed using network interaction tools.

### RNA FISH

CY3-labeled RNA FISH probes targeting *LncRNA-ROD* were designed and synthesized by GenePharma (Shanghai, China), with sequences listed in Table S4. RNA FISH was conducted according to the protocol provided with the RNA FISH Kit (GenePharma, for cell climbing slides). RAW 264.7 cells were seeded onto 24-well plates containing pretreated coverslips and induced for osteoclast differentiation. The cells were fixed with 4% paraformaldehyde at 0, 1, 3, and 5 days post-induction, followed by ethanol gradient dehydration. The probe mixture was prepared and incubated with the cells overnight at 37 °C for 12–16 h. Afterward, the cells were stained with DAPI (Sigma-Aldrich, D9542) for 20 min in the dark. Observations and imaging were performed using a confocal microscope (TCS-SP8, Leica Microsystems, Germany).

### RNA pulldown assay

Biotin-labeled full-length *LncRNA-ROD* probe (1147 bp) and control probes were synthesized by GenePharma. RNA pulldown assays were performed according to the instructions provided with the Pierce Magnetic RNA-Protein Pull-Down Kit (Thermo Fisher Scientific, 20164, Waltham, MA, USA). Lysates from RAW 264.7 cells were prepared ( ≥ 2 mg/mL protein concentration) and incubated with 50 pmol biotin-labeled RNA and 50 µL of streptavidin magnetic beads. After overnight incubation at 4 °C, the captured complexes were analyzed by mass spectrometry or Western blot.

### RIP assay

RIP assays were performed using the RIP Kit (BersinBio, Bes5101, Guangzhou, China) according to the manufacturer’s instructions. RAW 264.7 cells were induced for 3 days and then lysed. The lysates were pretreated to remove DNA. To capture RNA-protein complexes, the lysates were incubated overnight at 4 °C with 5 µg of QKI-specific antibody (Abcam, ab126742, Cambridge, UK) or an equivalent amount of rabbit IgG (CST, 2729S, Danvers, MA, USA). The immunoprecipitated RNA was subsequently purified and analyzed by RT-qPCR to confirm the binding of QKI to *LncRNA-ROD* or *Csf1r* transcripts.

### ChIP assay

ChIP assays were performed using the BeyoChIP Enzymatic ChIP Assay Kit (Beyotime, P2083S, Shanghai, China) according to the manufacturer’s instructions. At least 8 × 10^6^ RAW 264.7 cells were cross-linked with 1% formaldehyde. The cells were collected, and nuclei were isolated and treated with 125 gel units of MNase to shear genomic DNA into fragments between 150 and 1000 bp. Chromatin was immunoprecipitated using 2 µg of RUNX2-specific antibody (CST, 12556S) or an equal amount of rabbit IgG by rotation at 4 °C overnight. Immunocomplexes were collected and eluted, followed by protein removal to purify the DNA. The purified DNA was used for qPCR amplification of the *LncRNA-ROD* promoter region, specifically targeting potential RUNX2 binding sites within this region predicted by JASPAR (Cambridge, MA, USA). The primer sequences for the *LncRNA-ROD* promoter region are listed in Table S5.

### Dual-luciferase reporter assay

Plasmids expressing WT-RUNX2, MUT-RUNX2, and the 2 000 bp upstream promoter region of *LncRNA-ROD* (both wild-type and mutated versions) were co-transfected into cells with a Renilla luciferase plasmid at a ratio of 200 ng:800 ng:50 ng (transcription factor:promoter:Renilla). Firefly and Renilla luciferase activities were measured 24–48 h post-transfection using a multimode microplate reader (BioTek, Winooski, VT, USA) and the Dual-Luciferase Reporter Assay System (Promega, E1910, Madison, WI, USA). Data were normalized to Renilla luciferase activity.

### Micro-CT analysis

Alveolar bone samples were collected from the CCD patient and age- and gender-matched controls during supernumerary tooth surgery. Mandibles from PN11 and PN13 mice of various genotypes (*Runx2*^*+/+*^, *Runx2*^*+/-*^, *LncRNA-ROD*^*+/+*^, and *LncRNA-ROD*^*-/-*^) and femurs from ovariectomized and sham-operated mice were harvested and fixed in 4% paraformaldehyde at room temperature for 24 h. The samples were scanned with an Inveon micro-CT system (Siemens, Munich, Germany). The scanning parameters for alveolar bone were as follows: voltage, 80 kV; current, 500 µA; exposure time, 1 500 ms; and effective pixel size, 33.658 µm. The parameters for scanning the mandibles and femurs were: voltage, 60 kV; current, 220 µA; exposure time, 1 500 ms; and effective pixel size, 8.89 µm. Quantitative analysis and three-dimensional reconstruction were performed with Inveon Research Workplace software (Inveon Research Workplace 4.2, Siemens). Sagittal sections of the mandibles were selected to analyze the eruption level of the first molar. Eruption levels were quantified by measuring the vertical distance from the highest cusp tip to the highest point of the alveolar crest on the mesial side. The region of interest for femur analysis was located approximately 0.5 mm below the distal femoral growth plate. Osteoporosis severity was evaluated by calculating the bone mineral density (BMD, mg/cm^3^), bone volume fraction (BV/TV, %), trabecular thickness (Tb.Th, mm), trabecular number (Tb.N, 1/mm), and trabecular separation (Tb.Sp, mm).

### Statistical analysis

Data are presented as mean ± standard deviation (SD). Differences between two groups were evaluated using a two-tailed unpaired Student’s *t*-test. For comparisons among multiple groups, one-way ANOVA followed by Tukey’s multiple comparisons test was performed. Two-way ANOVA followed by Sidak’s multiple comparisons test was used for analyses involving two independent variables. A *P* < 0.05 was considered statistically significant. All statistical analyses were conducted using GraphPad Prism 8.0.1 (GraphPad Software, San Diego, CA, USA).

## Supplementary information

Supplemental_Material

## Data Availability

The RNA-seq datasets for this study have been previously deposited in NCBI (Data source: PRJNA1158440). All data are available in the main text or Supplemental Material.
