# Supplementary material for: RUNX2 promotes osteoclast differentiation and bone resorption via the newly identified LncRNA-ROD: implications for alveolar remodeling
Source: Int J Oral Sci. 2026 Sep 28;18:64. doi: 10.1038/s41368-026-00466-z (PMC13620161; doi:10.1038/s41368-026-00466-z)

Supplemental Material

Supplemental Materials and Methods

CRISPR/Cas9-mediated RUNX2 or *LncRNA-ROD* knockout

Single guide RNAs (sgRNAs) targeting exon 1 of the mouse *Runx2* gene (GenBank ID: NM_001271627.2) were designed with the CRImSPR Design Tool (Massachusetts Institute of Technology, Cambridge, MA, USA) to ensure high specificity and efficiency. The selected sgRNA sequences are listed below：

Runx2-sgRNA-1: 5'-GCCGCACGACAACCGCACCA-3';

Runx2-sgRNA-2: 5'-AAATCTCAGATCGTTGAACC-3';

Runx2-sgRNA-3: 5'-CGGCCGCAGTGCCCCGATTG-3'.

For *LncRNA-ROD*, sgRNAs targeting the entire exon were designed to achieve complete knockout by inducing large deletions; the sequences of these sgRNAs are listed below:

LncRNA-ROD -sgRNA-1: 5'-TGACTTATAACTCATGGGCG-3';

LncRNA-ROD -sgRNA-2: 5'-ACACCGGGAAACTGAGCAGG-3';

LncRNA-ROD -sgRNA-3: 5'-CGTGCGTGCATATTTAGGGG-3';

LncRNA-ROD -sgRNA-1: 5'-TGAAGCGTTCCTGTAAGACA-3'.

The sgRNAs were synthesized by GeneChem. Lentiviruses expressing these sgRNAs and Cas9 were transduced into RAW 264.7 cells. Single cells were sorted into 96-well plates with a BD FACSAria SORP cell sorter (BD Biosciences, San Jose, CA, USA). After proliferation, genomic DNA was extracted, and PCR followed by Sanger sequencing was used to confirm the successful knockout of *Runx2* or *LncRNA-ROD* in RAW 264.7 cells.

Cell culture

RAW 264.7 cells purchased from American Type Culture Collection (ATCC, Manassas, VA, USA) were cultured with DMEM supplemented with 10% FBS (OriCell, FBSAD-01011-500, Shenzhen, China) and 1% penicillin/streptomycin in a humidified incubator at 37°C with a 5% CO_2 atmosphere. The cells were passaged at a 1:6 ratio every other day.

RNA isolation and RT-qPCR

Total RNA of osteoclasts derived from RAW 264.7 cells, BMMs, or mouse tissues was extracted with TRIzol Reagent (Invitrogen, 15596018CN, Carlsbad, CA, USA) according to the manufacturer's instructions. cDNA was synthesized with PrimeScript RT Master Mix (Takara, RR036A, Kusatsu, Japan). RT-qPCR was performed with a FastStart Universal SYBR Green Master (ROX) (Roche, 4913914001, Basel, Switzerland) on a 7500 Real-Time PCR System (Thermo Fisher Scientific) following standard protocols. The sequences of primers used for gene amplification are listed below:

Rps18 F: 5'-CGACAGTCAGCCGCATCTT-3';

Rps18 R: 5'-CCAATACGACCAAATCCGTTG-3';

Runx2 F: 5'-GATGACACTGCCACCTCTGAC-3';

Runx2 R: 5'-GGGATGAAATGCTTGGGAAC-3';

LncRNA-ROD F: 5'-GGGCATCATACGATCGACTA-3';

LncRNA-ROD R: 5'-AGAAGAGAAGCCGAGGTGAG-3';

Dc-stamp F: 5'-GGGGACTTATGTGTTTCCACG-3';

Dc-stamp R: 5'-ACAAAGCAACAGACTCCCAAAT-3';

Ctsk F: 5'-GAAGAAGACTCACCAGAAGCAG-3';

Ctsk R: 5'-TCCAGGTTATGGGCAGAGATT-3';

Mmp9 F: 5'-CTGGACAGCCAGACACTAAAG-3’;

Mmp9 R: 5'-CTCGCGGCAAGTCTTCAGAG-3’;

Atp6v0d2 F: 5'-TCAGATCTCTTCAAGGCTGTGCTG-3’;

Atp6v0d2 R: 5'-GTGCCAAATGAGTTCAGAGTGATG-3’;

Itgb3 F: 5'-GATGACATCGAGCAGGTGAAAGAG-3’;

Itgb3 R: 5'-CCGGTCATGGATAGTGATGAGTAG-3’;

Cd44 F: 5'-GGACTCCAGGGGGAGTTCCCGCAC-3’;

Cd44 R: 5'-CGTCCCATTGCCACCGTTGATCAC-3’;

Oc-stamp F: 5'-GGGCTACTGGCATTGCTCTTAGT-3’;

Oc-stamp R: 5'-CCAGAACCTTATATGAGGCGTCA-3’;

Itgav F: 5'-CCTCAGAGAGGGAGATGTTCACAC-3’;

Itgav R: 5'-AACTGCCAAGATGATCACCCACAC-3’;

Cd9 F: 5'-CGGTCAAAGGAGGTAG-3’;

Cd9 R: 5'-GGAGCCATAGTCCAATA-3’;

Cd47 F: 5'-TGGTGGGAAACTACACTTGCGA-3’;

Cd47 R: 5'-AGGCTGATCCTTGGTCAGTGTTG-3’;

Adam12 F: 5'-AAATCCCACGACAATGCTCAGC-3’;

Adam12 R: 5'-CCAGCTCATGTGCCAAGGTCA-3’;

Tyrobp F: 5'-TGGTGTTGACTCTGCTGATTGC-3’;

Tyrobp R: 5'-CCTTCCGCTGTCCCTTGAC-3’;

Sirpa F: 5'-AAATCAGTGTCTGTTGCTGCTGG-3’;

Sirpa R: 5'-CTGGGGTGACATTACTGATAC-3’;

Ptk2 F: 5'-GGCAGCTGCTTATCTTGACC-3’;

Ptk2 R: 5'-TGATGCCCCTGACATCAGTA-3’;

Trem2 F: 5'-GACCTCTCCACCAGTTTCTCC-3’;

Trem2 R: 5'-TACATGACACCCTCAAGGACTG-3’;

Cd200 F: 5'-CTTGATTGTGACATGGCAGAAA-3’;

Cd200 R: 5'-AAACGTGTTGAAGAGACACATG-3’;

Cdh1 F: 5'-CTCAGAAGACAGAAACGAGACT-3’;

Cdh1 R: 5'-AACCAGGTTCTTTGGAAATTCG-3’;

Ccl2 F: 5'-TTTTTGTCACCAAGCTCAAGAG-3’;

Ccl2 R: 5'-TTCTGATCTCATTTGGTTCCGA-3’.

Protein extraction and Western blot analysis

RAW 264.7 cells or BMMs were lysed in RIPA lysis buffer (Huaxingbio, HX1863, Zhengzhou, China) containing protease inhibitors (Huaxingbio, HX1862). Protein concentrations were measured with a BCA assay (Thermo Fisher Scientific, A55864). Protein samples were separated by 10% SDS–PAGE (Biotides, WB1102, Beijing, China) and transferred to 0.45 μm PVDF membranes (Millipore, IPVH00010, Burlington, MA, USA). The membranes were blocked with 5% nonfat milk (Huaxingbio, HX1866) and incubated with primary antibodies at 4°C on a shaker overnight. HRP-conjugated secondary antibodies (anti-rabbit, 1:20 000, Proteintech, SA00001-2, Rosemont, IL, USA) were then incubated on a shaker at room temperature for 1 h. The membranes were incubated with enhanced chemiluminescence substrate (NCM Biotech, P10300, Suzhou, China), and bands were visualized with a chemiluminescent imaging system (Vilber Lourmat, Collégien, France) and analyzed with Evolution Capt Edge software (Vilber Lourmat).

The primary antibodies used were as follows: anti-RUNX2 (1:1 000, CST, 12556S), anti-CTSK (1:1 000, Abcam, ab187647), anti-MMP9 (1:1 000, CST, 24317T), anti-QKI (1:1 000, Abcam, ab126742), anti-CSF1R (1:1 000, Abcam, ab254357), anti-CD44 (1:1 000, Abcam, ab189524), anti-ITGB3 (1:1 000, Abcam, ab179473), and anti-GAPDH (1:2 000, CST, 5174S).

3' and 5' RACE

Total RNA was extracted from RAW 264.7 cells after 3 days of osteoclast induction to clone the full-length cDNA sequence of *LncRNA-ROD*. The experiment was performed according to the protocol of the SMARTer RACE 5'/3' Kit (Clontech, 634858, Mountain View, CA, USA). The gene-specific primers used for amplification of the 3' and 5' end sequences are listed below:

3'RACE F2: 5'-TCTAGTTATGGACCCGTCAGCTTT-3';

3'RACE F3: 5'-TCTTATAGAAGGTGCAGGTCAGAG-3';

5'RACE R2: 5'-ACTGTTGGTTTACTTCCTGTGTCC-3';

5'RACE R3: 5'-GGCACATTGCTCATAGTCGATCGT-3'.

Generation of global knockout *LncRNA-ROD* mice

Global knockout *LncRNA-ROD* mice (C57BL/6) were generated with the CRISPR/Cas9 system and bred by Cyagen Biosciences (Guangzhou, China). The exon 1 to exon 3 region of *LncRNA-ROD* was selected as the target for four sgRNAs. The sgRNA sequences are listed below:

LncRNA-ROD-sgRNA-A1: 5'-AAGGGTGAAGGACGTTCACGGGG-3';

LncRNA-ROD-sgRNA-A2: 5'-GGATTCATTACTGCGGGACACGG-3';

LncRNA-ROD-sgRNA-B1: 5'-AGCCACGGCTGCTCACGGGTCGG-3';

LncRNA-ROD-sgRNA-B2: 5'-AAAGGGCTTTACAACGAAACAGG-3'.

Genotypes were determined by PCR amplification of tail-derived genomic DNA.

Whole-mount skeletal staining

Within 24 h after birth (PN0), neonatal mice were euthanized via carbon dioxide asphyxiation. The specimens were immersed in absolute ethanol for 24 h, followed by removal of the skin and as much soft tissue as possible. To soften the remaining soft tissue and prepare the bodies for staining, the specimens were then immersed in absolute ethanol for another 24 h. Alcian blue (A5533; Sigma-Aldrich) and Alizarin red (A3157; Sigma-Aldrich) stain solutions were prepared. The mice were soaked in Alcian blue stain and incubated overnight on a shaker. After rinsing with running water, the specimens were immersed in 1% NaOH until the thoracic cavity became transparent and the ribs were visible. The mice were then soaked in Alizarin red stain to color the ossified bones, followed by immersion in 3% H_2O_2 overnight. The mice were cleared through a gradient glycerol series, ultimately resulting in immersion in pure glycerol. Imaging was performed using a DSLR camera (Nikon D850, Nikon, Japan).

Mouse osteoporosis model

Eight-week-old female C57BL/6 mice were divided into the OVX and sham operation groups. The mice were anesthetized via an intraperitoneal injection of tribromoethanol (Avertin, Sigma-Aldrich, T48402). A midline abdominal incision was made to ligate the bilateral fallopian tubes and remove the bilateral ovaries. The OVX groups underwent fallopian tubes ligation and ovariectomy surgery to induce estrogen deficiency and mimic osteoporosis, while the sham groups underwent a control surgery without removal of the ovaries or ligation of the fallopian tubes. The incision was closed with interrupted sutures using 4-0 absorbable sutures (Ethicon, J495H,

Somerville, NJ, USA). Eight weeks post-surgery, the mice were euthanized by cervical dislocation, and the femurs were collected for subsequent experimental analysis.

Detection of RNA stability

RAW 264.7 cells were seeded at a density of 5×10^5 cells/mL in 24-well plates and were cultured overnight. The following day, the medium was replaced with fresh medium containing actinomycin D (CST, 15021S) at a final concentration of 5 μg/mL. The cells were then harvested at 0, 2, 4, and 6 h. RNA extraction and RT-qPCR were performed as previously described to calculate RNA degradation rates.

AAV infection

Recombinant AAV9 (rAAV9) vectors carrying sh*Qki* or shNC were designed and synthesized by GeneChem (Shanghai, China) with the U6-MCS-CAG-EGFP vector; the sequences are listed below:

AAV-shNC: 5'-AAGGGTGAAGGACGTTCACGGGG-3';

AAV-sh*Qki*: 5'-GGATTCATTACTGCGGGACACGG-3'.

Two weeks after OVX or sham surgery, the mice received a tail vein injection of rAAV9 carrying shNC or sh*Qki* at a dose of 4×10^{11} GCs in a total volume of 200 μL. Two weeks after the tail vein injection, EGFP expression was measured to evaluate the distribution of AAV in the tissue with an IVIS Spectrum imaging system (PerkinElmer, Waltham, MA, USA). Eight weeks after the tail vein injection, the mice were euthanized by cervical dislocation, and the femurs were collected for subsequent experimental analysis.

Histopathological evaluation

After micro-CT scanning, the bone samples were decalcified in EDTA (pH 7.4) and embedded in paraffin. Sections (5 μm thick) were prepared and stained with HE (Solarbio, G1120) and TRAP (Sigma-Aldrich, 387A) according to the manufacturers' instructions. Images were captured with a light microscope (Olympus, IX53).

The immunohistochemical staining was performed following standard procedures. The primary antibodies included QKI (1:400; Proteintech, 13169-1-AP). A non-biotin detection system (Zsbio, PV-6001) was utilized. Hematoxylin (Zsbio, ZLI-9610, Beijing, China) was applied for nuclear staining. Images were acquired using the NanoZoomer S210 (Hamamatsu, Hamamatsu, Japan).

Quantitative analyses were conducted with Visiopharm software (VISIOPHARM, Hørsholm, Denmark). Cells were considered as positive labels, and staining intensities were differentiated into 3+, 2+, and 1+ based on DAB values. The H-SCORE was calculated, which is defined as: (positive rate of strongly positive cells × 300) + (positive rate of moderately positive cells × 200) + (positive rate of weakly positive cells × 100).

Supplemental Figure Legends

Supplemental Fig. 1 *Runx2*$^{+/-}$ mice exhibit partially resolved eruption of M1 and M2 but sustained delay of M3 at PN21. Representative sagittal and three-dimensional reconstructed views of mandibular molars in *Runx2*$^{+/-}$ at PN21 (n = 6). Eruption levels were quantified in the sagittal plane by measuring the vertical distance from the highest cusp tip to the highest point of the alveolar crest on the mesial side of the mandibular first molars. Red lines indicate the highest point of the alveolar crest. Data are presented as mean ± SD

Supplemental Fig. 2 RUNX2 knockout inhibits osteoclast differentiation and bone resorption. **a** Schematic representation showing the use of the CRISPR/Cas9 system to generate *Runx2* KO RAW 264.7 cells. **b** Sanger sequencing confirming the successful construction of the *Runx2* KO cells. **c** Predicted structure of the RUNX2 protein. **d** RT-qPCR analysis of osteoclast-related gene expression in *Runx2* KO. **e** Quantification of Western blot data corresponding to Fig. 3a (n = 3). **f** RT-qPCR analysis of osteoclast-related gene expression in *Runx2*$^{+/-}$ BMMs. **g** Quantification of Western blot data corresponding to Fig. 3d (n = 3). **h** Experimental design illustrating the impact of RUNX2 knockout on osteoclast differentiation in the OVX mouse model. **i** Images of HE staining of femoral. Data are presented as mean ± SD

Supplemental Fig. 3 RT-qPCR analysis of the expression of differentially expressed lncRNAs during osteoclast differentiation (**a**) and in different cell lines (**b**, **d**) and mouse tissues (**c**, **e**). Data are presented as mean ± SD

Supplemental Fig. 4 *LncRNA-ROD* has no protein-coding potential. **a** Full-length cDNA sequence of *LncRNA-ROD*. Identical sequences between *LncRNA-ROD* and *4833415N18Rik* are underlined. **b** CPAT analysis showing that *LncRNA-ROD* lacks protein-coding potential.

Supplemental Fig. 5 Transcriptional activation effect of WT and Mutant RUNX2 on the *LncRNA-ROD* promoter. Data are presented as mean ± SD

Supplemental Fig. 6 *LncRNA-ROD* is co-expressed with RUNX2 and CTSK in bone tissue and mediates RUNX2-dependent osteoclastogenesis. **a**–**c** Representative co-localization images in mandibular alveolar bone from wild-type mice at PN5. **a** Immunofluorescence staining of RUNX2 and CTSK. **b** *LncRNA-ROD* RNA FISH combined with CTSK immunofluorescence. **c** RUNX2 immunofluorescence combined with *LncRNA-ROD* RNA FISH. **d**–**f** Representative co-localization images in femoral bone from 8-week-old wild-type mice. **d** Immunofluorescence staining of RUNX2 and CTSK. **e** *LncRNA-ROD* RNA FISH combined with CTSK immunofluorescence. **f** RUNX2 immunofluorescence combined with *LncRNA-ROD* RNA FISH. The white dotted lines in panels d–f indicate the boundary between the growth plate and metaphysis. **g** RT-qPCR analysis of *LncRNA-ROD* expression following overexpression**,** and of osteoclast-related genes and *Csf1r* (n = 3). **h** Quantification of Western blot data corresponding to Fig. 5a (n = 3). Data are presented as mean ± SD

Supplemental Fig. 7 *LncRNA-ROD*$^{-/-}$ mice exhibit partially resolved eruption of M1 and M2 but sustained delay of M3 at PN21. Representative sagittal and three-dimensional reconstructed views of mandibular molars in *LncRNA-ROD*$^{-/-}$ at PN21 (n = 6). Eruption levels were quantified in the sagittal plane by measuring the vertical distance from the highest cusp tip to the highest point of the alveolar crest on the mesial side of the mandibular first molars. Red lines indicate the highest point of the alveolar crest. Data are presented as mean ± SD

Supplemental Fig. 8 Overexpression of *LncRNA-ROD* reduces osteoclast fusion but enhances bone resorption capacity. **a** RT-qPCR analysis of osteoclast-related gene expression in OE-*LncRNA-ROD* cells (n = 3). **b** Western blot analysis of osteoclast-related gene expression in OE-

LncRNA-ROD cells. **c**, **d** Representative images of TRAP staining (**c**) and bone resorption pits (**d**) in OE-*LncRNA-ROD* cells (*n* = 3). Data are presented as mean ± SD

Supplemental Fig. 9 *LncRNA-ROD* knockout enhances osteoclast fusion but reduces bone resorption capacity. **a** Schematic representation showing the use of the CRISPR/Cas9 system to generate *LncRNA-ROD* KO RAW 264.7 cells. **b** Sanger sequencing confirming the successful construction of the *LncRNA-ROD* KO cells. **c** RT-qPCR analysis of *LncRNA-ROD* expression in *LncRNA-ROD* KO cells (*n*=3). **d** Quantification of Western blot data corresponding to Fig. 7a (*n* = 3). **e** RT-qPCR analysis of fusion-related gene expression levels during osteoclast differentiation following *LncRNA-ROD* knockout. **f** Quantification of Western blot data corresponding to Fig. 7d (*n* = 3). Data are presented as mean ± SD

Supplemental Fig. 10 *LncRNA-ROD* knockout promotes osteoclast fusion. TRAP staining images in *LncRNA-ROD*$^{-/-}$-BMMs during osteoclastogenic induction for the indicated times.

Supplemental Fig. 11 *LncRNA-ROD* knockout enhances osteoclast fusion but reduces bone resorption capacity. **a** Experimental design illustrating the impact of *LncRNA-ROD* KO on osteoclast differentiation in the OVX mouse model. **b** Images of HE staining of femoral sections. **c**, **d** Representative immunofluorescence staining images of CTSK (**c**) and MMP9 (**d**) in femoral sections. The white dotted lines indicate the boundary between the growth plate and metaphysis.

Supplemental Fig. 12 *LncRNA-ROD* interacts with QKI to regulate osteoclast differentiation and bone resorption. **a** Quantification of Western blot data corresponding to Fig. 8a (*n* = 3). **b** Subcellular localization of QKI during osteoclast differentiation detected by immunofluorescence. **c** RT-qPCR analysis of osteoclast-related gene expression in sh*Qki* cells (*n* = 3). **d** Quantification of Western blot data corresponding to Fig. 8c (*n* = 3). **e** Quantification of Western blot data

corresponding to Fig. 8f (*n* = 3). **f** Quantification of Western blot data corresponding to Fig. 8g (*n* = 3). Data are presented as mean ± SD

Supplemental Fig. 13 Representative immunofluorescence staining of CSF1R in femoral sections from *LncRNA-ROD*$^{-/-}$ mice. The white dotted lines indicate the boundary between the growth plate and metaphysis.

Supplemental Fig. 14 QKI-5 is the dominant isoform negatively regulating osteoclastogenesis and engages *LncRNA-ROD* and *Csf1r* mRNA via dual-compartment binding. **a** RT-qPCR analysis of *Qki-5* knockdown efficiency and osteoclast-related gene expression in si-*Qki-5* cells. **b** Western blot analysis of QKI-5, QKI-6, QKI-7, and osteoclast-related protein expression in si-*Qki-5* cells. **c**, **d** Representative images of TRAP staining (**c**) and bone resorption pits (**d**) in si-*Qki-5* cells (*n* = 3). **e** RIP-qPCR showing that QKI-5 and QKI-6, but not QKI-7, bind to *LncRNA-ROD*. **f** RIP-qPCR showing that QKI-5 and QKI-6, but not QKI-7, bind to *Csf1r* mRNA. Data are presented as mean ± SD

Supplemental Fig. 15 Intravenous injection of AAV-sh*Qki* partially rescues osteoclast differentiation defects caused by RUNX2 or *LncRNA-ROD* knockout. **a** Schematic illustration of the experimental design for investigating the molecular mechanism by which RUNX2 regulates osteoclast differentiation and bone resorption through the *LncRNA-ROD*/QKI axis. **b** Schematic illustration of the experimental design for investigating the molecular mechanism by which *LncRNA-ROD* regulates osteoclast differentiation and bone resorption through QKI.

Supplemental Fig. 16 In vivo fluorescence imaging of EGFP expression in organs and whole body after tail vein injection of AAV-sh*Qki*. A single dose of 4×10^{11} genome copies of rAAV9-shNC-EGFP or rAAV9-shQki-EGFP was intravenously (i.v.) injected into 2-month-old mice.

EGFP expression was monitored by IVIS-100 optical imaging 2 weeks post-injection. Representative images of EGFP expression in various organs (**a**) and whole-body images (**b**).

Supplemental Fig. 17 IHC of QKI staining images of femoral sections from *Runx2* knockout or *LncRNA-ROD* knockout groups after tail vein injection of AAV-sh*Qki*. **a** *Runx2*$^{+/-}$ mice: IHC of QKI staining images and H-Score of QKI of femoral sections from different experimental groups (n = 6). **b** *LncRNA-ROD*$^{-/-}$ mice: IHC of QKI staining images and H-Score of QKI of femoral sections from different experimental groups (n = 6). Data are presented as mean ± SD

Supplemental Fig. 18 Intravenous injection of AAV-sh*Qki* partially rescues osteoclast differentiation defects caused by RUNX2 or *LncRNA-ROD* knockout. **a** *Runx2*$^{+/-}$ mice: Images of HE staining of femoral sections. **b** *LncRNA-ROD*$^{-/-}$ mice: Images of HE staining of femoral sections.

Supplemental Tables

Supplemental Table 1. Raw micro-CT parameters of alveolar bone from the CCD patient and age- and sex-matched controls.

Group	Subject ID	BMD (mg/cm3)	BV/TV (%)
Control	Control 1	1313.79	20.591
Control	Control 2	1225.352	29.397
Control	Control 3	1299.387	25.492
CCD	Sample 1	1488.87	42.656
CCD	Sample 2	1528.352	37.926
CCD	Sample 3	1394.323	40.163

Supplemental Table 2 Potential RUNX2 binding sites on *LncRNA-ROD* promotor predicted by JASPAR.

Matrix ID	Name	Score	Relative score	Sequence ID	Start	End	Strand	Predicted sequence
MA0511.2	RUNX2	11.83120	0.94295	*LncRNA-ROD*	638	646	+	CAACCACAA
MA0511.2	RUNX2	8.88410	0.88863	*LncRNA-ROD*	1430	1438	-	TACCCACAA
MA0511.2	RUNX2	8.55807	0.88262	*LncRNA-ROD*	1988	1996	+	AAACCCCAA

Supplemental Table 3 Mass spectrometry identification from RNA pulldown assay.

Accession	Description	*LncRNA-ROD*: Area	*AS-LncRNA-ROD*: Area	*LncRNA-ROD: Area/AS-LncRNA-ROD*: Area	MW [kDa]
E9QP59	Inner nuclear membrane protein Man1 OS=Mus musculus OX=10090 GN=Lemd3 PE=1 SV=1 - [E9QP59_MOUSE]	1.856E6	0.000E0	#DIV/0!	100.1
Q9CSH3	Exosome complex exonuclease RRP44 OS=Mus musculus OX=10090 GN=Dis3 PE=1 SV=4 - [RRP44_MOUSE]	4.959E6	0.000E0	#DIV/0!	108.8
Q8K114	Integrator complex subunit 9 OS=Mus musculus OX=10090 GN=Ints9 PE=1 SV=1 - [INT9_MOUSE]	1.352E6	0.000E0	#DIV/0!	74.0
Q99LL5	Periodic tryptophan protein 1 homolog OS=Mus musculus OX=10090 GN=Pwp1 PE=1 SV=1 - [PWP1_MOUSE]	1.728E6	0.000E0	#DIV/0!	55.6
P70700	DNA-directed RNA polymerase I subunit RPA2 OS=Mus musculus OX=10090 GN=Polr1b PE=1 SV=2 - [RPA2_MOUSE]	4.576E7	9.855E6	4.64	128.1
E9Q5A3	Histone-lysine N-methyltransferase EHMT1 OS=Mus musculus OX=10090	8.780E6	2.552E6	3.44	136.6

	GN=Ehmt1 PE=1 SV=2 - [E9Q5A3_MOUSE]				
P20152	Vimentin OS=Mus musculus OX=10090 GN=Vim PE=1 SV=3 - [VIME_MOUSE]	4.638E7	1.534E7	3.02	53.7
Q6P5B0	RRP12-like protein OS=Mus musculus OX=10090 GN=Rrp12 PE=1 SV=1 - [RRP12_MOUSE]	3.274E7	1.139E7	2.88	143.0
Q8BK35	Ribosome biogenesis protein NOP53 OS=Mus musculus OX=10090 GN=Nop53 PE=1 SV=1 - [NOP53_MOUSE]	8.782E6	3.143E6	2.79	55.8
Q8CCS6	Polyadenylate-binding protein 2 OS=Mus musculus OX=10090 GN=Pabpn1 PE=1 SV=3 - [PABP2_MOUSE]	1.257E7	4.998E6	2.51	32.3
Q62318	Transcription intermediary factor 1-beta OS=Mus musculus OX=10090 GN=Trim28 PE=1 SV=3 - [TIF1B_MOUSE]	2.469E7	1.091E7	2.26	88.8
Q80UK8	Integrator complex subunit 2 OS=Mus musculus OX=10090 GN=Ints2 PE=1 SV=2 - [INT2_MOUSE]	3.450E6	1.537E6	2.24	133.4
Q3TMP1	General transcription factor IIIC, polypeptide 3 OS=Mus musculus OX=10090 GN=Gtf3c3 PE=1 SV=1 - [Q3TMP1_MOUSE]	7.180E6	3.321E6	2.16	100.6

F8WHY8	Metastasis-associated protein MTA1 OS=Mus musculus OX=10090 GN=Mta1 PE=1 SV=1 - [F8WHY8_MOUSE]	2.107E7	9.840E6	2.14	79.1
Q9QYS9	Protein quaking OS=Mus musculus OX=10090 GN=Qki PE=1 SV=1 - [QKI_MOUSE]	1.065E7	5.075E6	2.10	37.6

Supplemental Table 4 RNA FISH probe sequences for *LncRNA-ROD*.

Name	Sequence (5'-3')
LncRNA-ROD #1	AGCCTCTTCCATAGCCCTGT
LncRNA-ROD #2	CACTTCACTACTATCTCTTG
LncRNA-ROD #3	AAGCTGACGGGTCCATAACT
LncRNA-ROD #4	ATTTCCTCCGCCCTAAAGGT

Supplemental Table 5 Primer sequences used for ChIP-qPCR.

Name	Forward 5'-3'	Reverse 5'-3'
Primer 1	CACAAGACATGCAGAGAGGC	TGACTCTCTTTCAGGCCCTG
Primer 2	CATAGGGCTGCAACCACAAG	TCTAAGGAATGTCGCGGAGG
Primer 3	CAGCTCCAGGAGAATGTTTGA	TCTGCATGTCTTGTGGTTGC

Supplemental Figures

Fig. S1

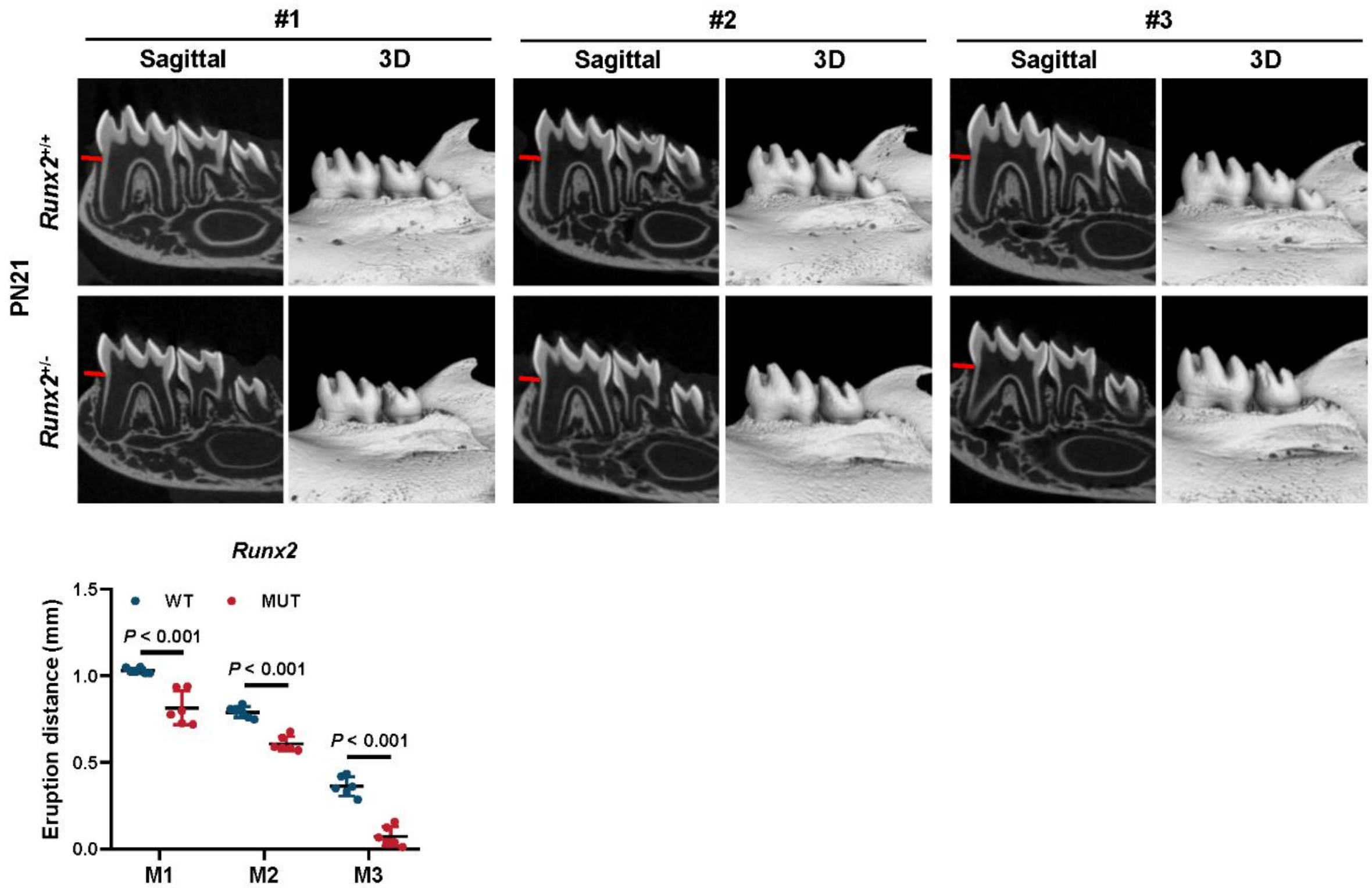

Fig. S2

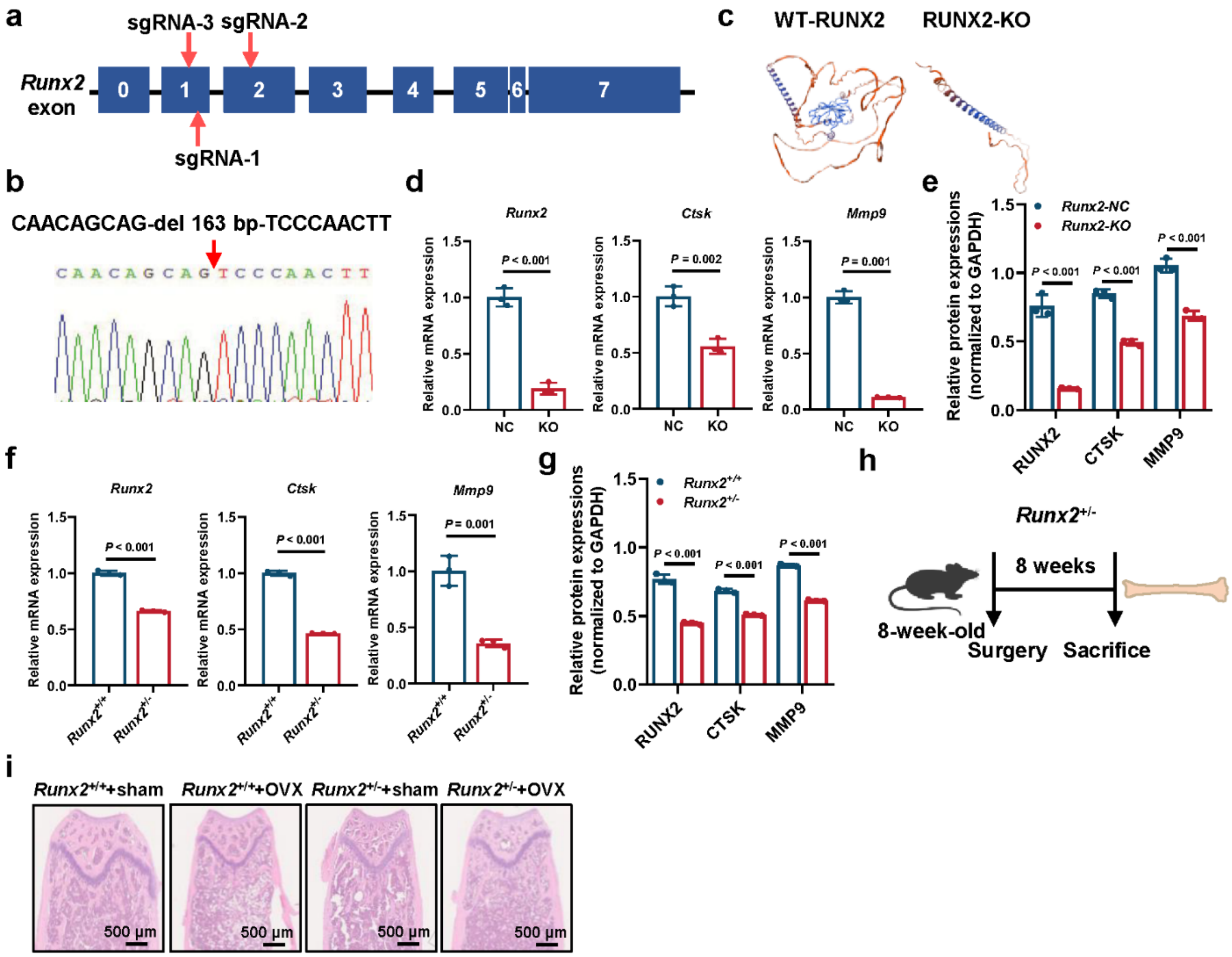
a
sgRNA-3 sgRNA-2
Runx2 exon
0 1 2 3 4 5 6 7
sgRNA-1
b
CAACAGCAG-del 163 bp-TCCCAACTT
CAACAGCAGTCCCAACTT
c
WT-RUNX2
RUNX2-KO
d
Runx2
Ctsk
Mmp9
Relative mRNA expression
P < 0.001
P = 0.002
P = 0.001
NC KO
e
Runx2-NC
Runx2-KO
Relative protein expressions (normalized to GAPDH)
P < 0.001
RUNX2 CTSK MMP9
f
Runx2
Ctsk
Mmp9
Relative mRNA expression
P < 0.001
P = 0.001
Runx2+/+ Runx2+/-
g
Runx2+/+
Runx2+/-
Relative protein expressions (normalized to GAPDH)
P < 0.001
RUNX2 CTSK MMP9
h
Runx2+/-
8 weeks
8-week-old
Surgery
Sacrifice
i
Runx2+/++sham
Runx2+/++OVX
Runx2+/-+sham
Runx2+/-+OVX
500 μm

Fig. S3

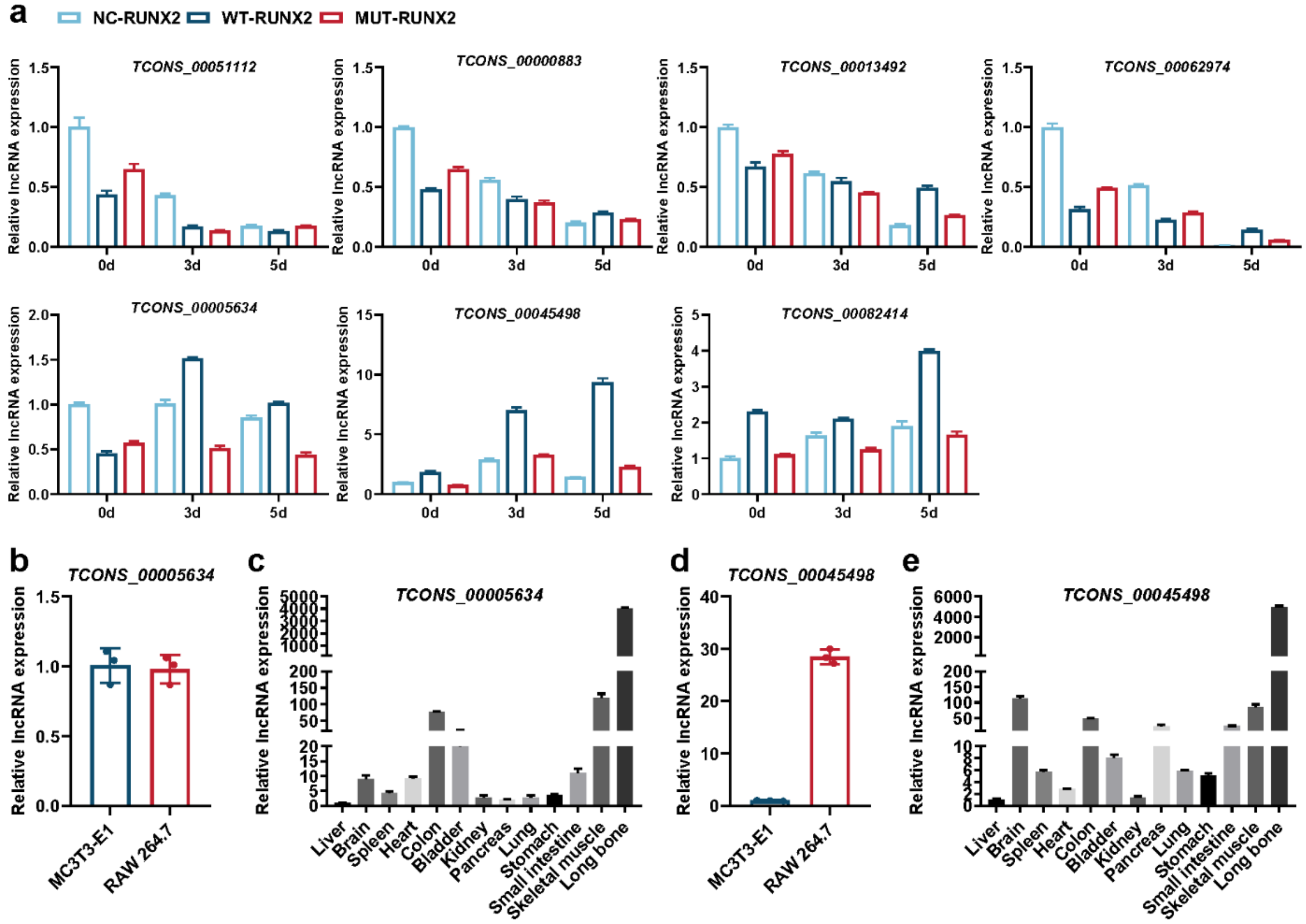

a
NC-RUNX2
WT-RUNX2
MUT-RUNX2
TCONS_00051112
TCONS_00000883
TCONS_00013492
TCONS_00062974
TCONS_00005634
TCONS_00045498
TCONS_00082414
Relative lncRNA expression
0d
3d
5d
b
TCONS_00005634
MC3T3-E1
RAW 264.7
c
TCONS_00005634
d
TCONS_00045498
e
TCONS_00045498
Liver
Brain
Spleen
Heart
Colon
Bladder
Kidney
Pancreas
Lung
Stomach
Small intestine
Skeletal muscle
Long bone

Fig. S4

a

5’-
AGACTCTCAAAGACAGCTGGATACCCCACACCGGGAAACTGAGCAGGAAGAAACTTGCCCTGGCAATCTACACAATTTCCA
GTGGGAGTGGCACTATTAGGAAGTATGGCCTGGTTGGAGGAAGCGTGTGTCTGTGGGATGGGCTTTGAGAGCCTCCTCCTA
GCTGCCCATGAGCCAGTCTTCTTCTAGCAGACTTCAGATGAAGATGTAGAACTCTCAGCTCCTCCTACACCATGCCTGCCTG
GATACTGCCATGCTCCTGCCTTGATGATAATGGACTGAACCTCTGAACCTATGAGGAGATGAGACTCCCAGGAGGATGAGGG
CCACCCACAGGGCTATGGAAGAGGCTCAAGACTGATGAGGAAGGAAGAAAATCAGCTGGATCCTCAAGACACAAACAGAC
TAACAGACTCTTTCATTGCAACCTTTAGGGCGGAGGAAATTTGACATAGGATGCAGGGGCATCATACGATCGACTATGAGCA
ATGTGCCCACCGCGTGAGCCTCTCAGGACACAGGAAGTAAACCAACAGTCTCACTTTCCAGTGTCTCACCTCGGCTTCTCTT
CTGTTCTCTTTAATTTGTCTTCAGAGCACTGAGCAGTGGTCGTCCTTGGTCCCAGACAATACTGTCTCTAAGAGAAGACCAAC
ACAGTTAGAGACAGGACGGCCCAAAGGAGAATCAGATCCGTCCAGTTTAGGCCATGTATCTAGTTATGGACCCGTCAGCTTT
GACAAAGGAGCTAGGGTCTTGTAGCGTGCTTGTTCTTATAGAAGGTGCAGGTCAGAGGGCCTGGCTGCCCACGTCCTCCTT
GGAGACTCAGAAAGAAATAAGAATTTCAAGAGATAGTAGTGAAGTGGTTTACAGAAAAGAATTTCCGAATTTTCCTTTTCTTT
TCCTCAGTCCTTATTGCATTAACCTTGTGATTGTCAACATACTTGTGTGTTGCTGAATATTTTAAGAAAAATCAACAAATGTTAC
CTTGAAACAATAACAAAGCCCAGAAAAATGCAAAACAGCCTTGGTCTGTCCCAGCGTCTTTCATCTGAGAGCACAGAGAGC
CAGCGCTAGCCTGGGGAGGGGGTGTGTGGCTAGAATGTGTGTGTGCTCAGGGCTTTCATTAAAAACACATTTTATTTACTCTA
AAAAAAAAAAAAAAA-3’

b *LncRNA-ROD*

RNA size	ORF size	Ficket score	Hexamer score	Coding probability	Coding Label
1147	249	0.5887	-0.122979325806	0.01169618899533	no

Fig. S5

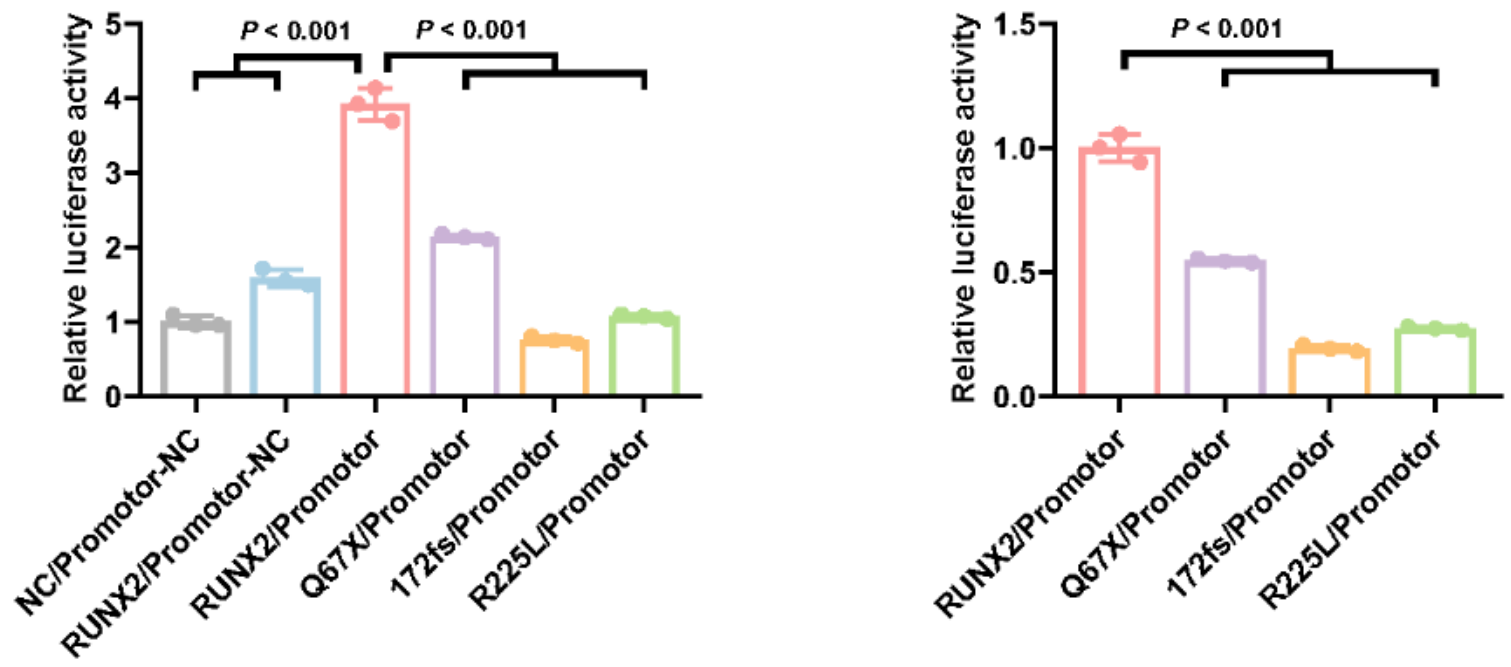

Relative luciferase activity
P < 0.001
P < 0.001
NC/Promotor-NC
RUNX2/Promotor-NC
RUNX2/Promotor
Q67X/Promotor
172fs/Promotor
R225L/Promotor
Relative luciferase activity
P < 0.001
RUNX2/Promotor
Q67X/Promotor
172fs/Promotor
R225L/Promotor

Fig. S6

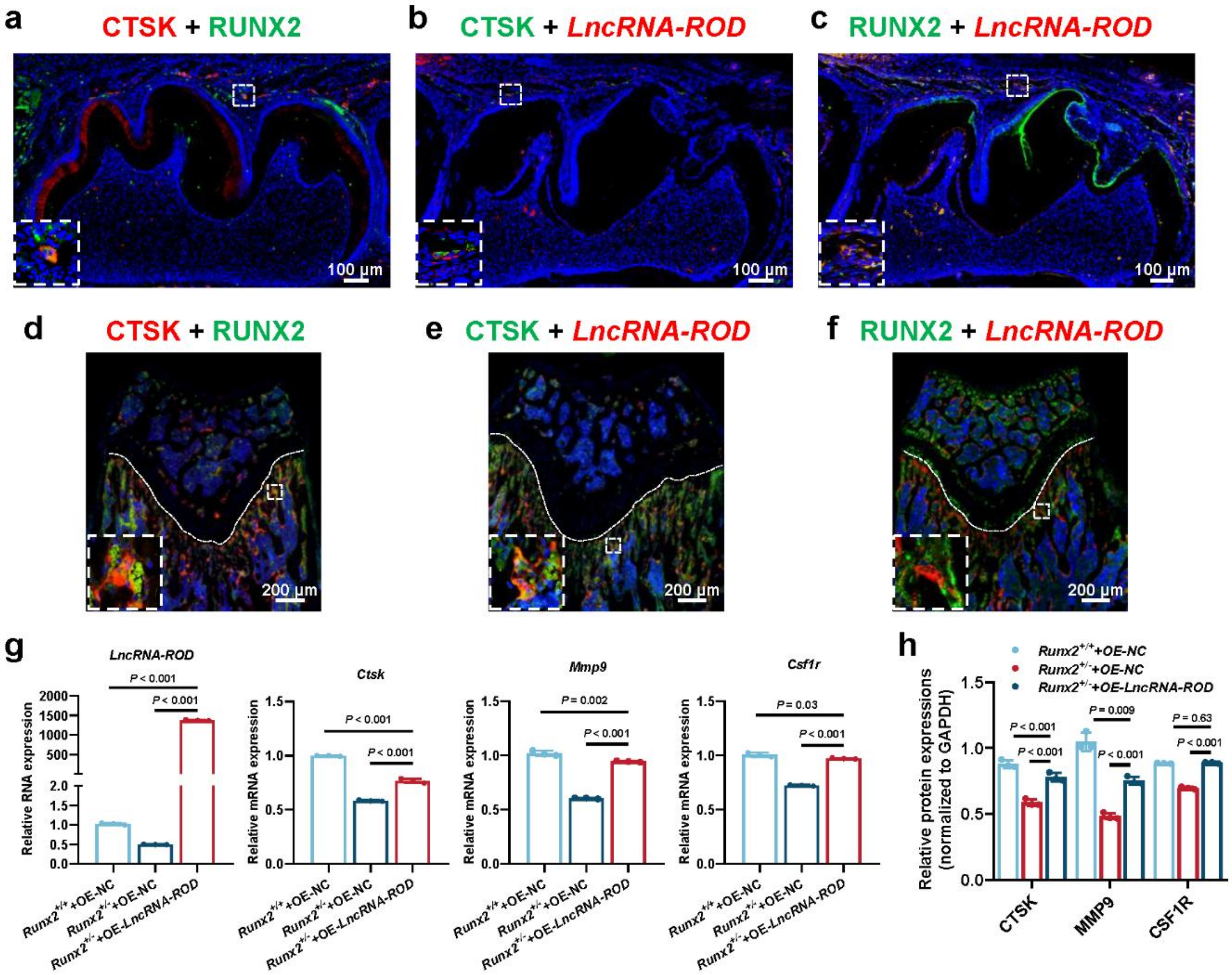

a
CTSK + RUNX2
b
CTSK + LncRNA-ROD
c
RUNX2 + LncRNA-ROD
100 µm
d
CTSK + RUNX2
e
CTSK + LncRNA-ROD
f
RUNX2 + LncRNA-ROD
200 µm
g
LncRNA-ROD
Ctsk
Mmp9
Csf1r
Relative RNA expression
Relative mRNA expression
P < 0.001
P = 0.002
P = 0.03
Runx2+/+ +OE-NC
Runx2+/- +OE-NC
Runx2+/- +OE-LncRNA-ROD
h
Runx2+/+ +OE-NC
Runx2+/- +OE-NC
Runx2+/- +OE-LncRNA-ROD
Relative protein expressions (normalized to GAPDH)
P = 0.009
P = 0.63
CTSK
MMP9
CSF1R

Fig. S7

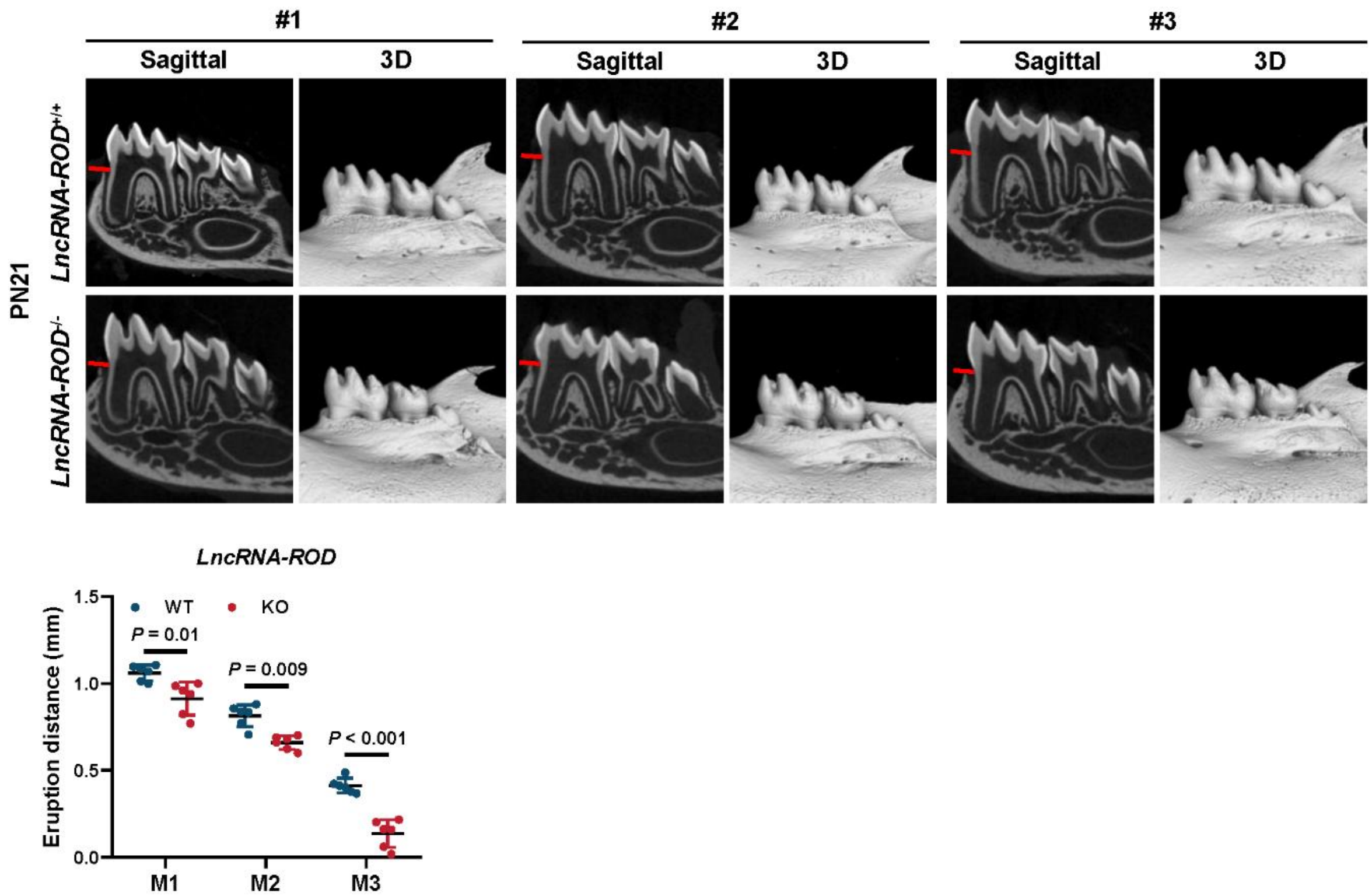
#1
#2
#3
Sagittal
3D
Sagittal
3D
Sagittal
3D
PN21
LncRNA-ROD+/+
LncRNA-ROD-/-
LncRNA-ROD
WT
KO
P = 0.01
P = 0.009
P < 0.001
Eruption distance (mm)
1.5
1.0
0.5
0.0
M1
M2
M3

Fig. S8

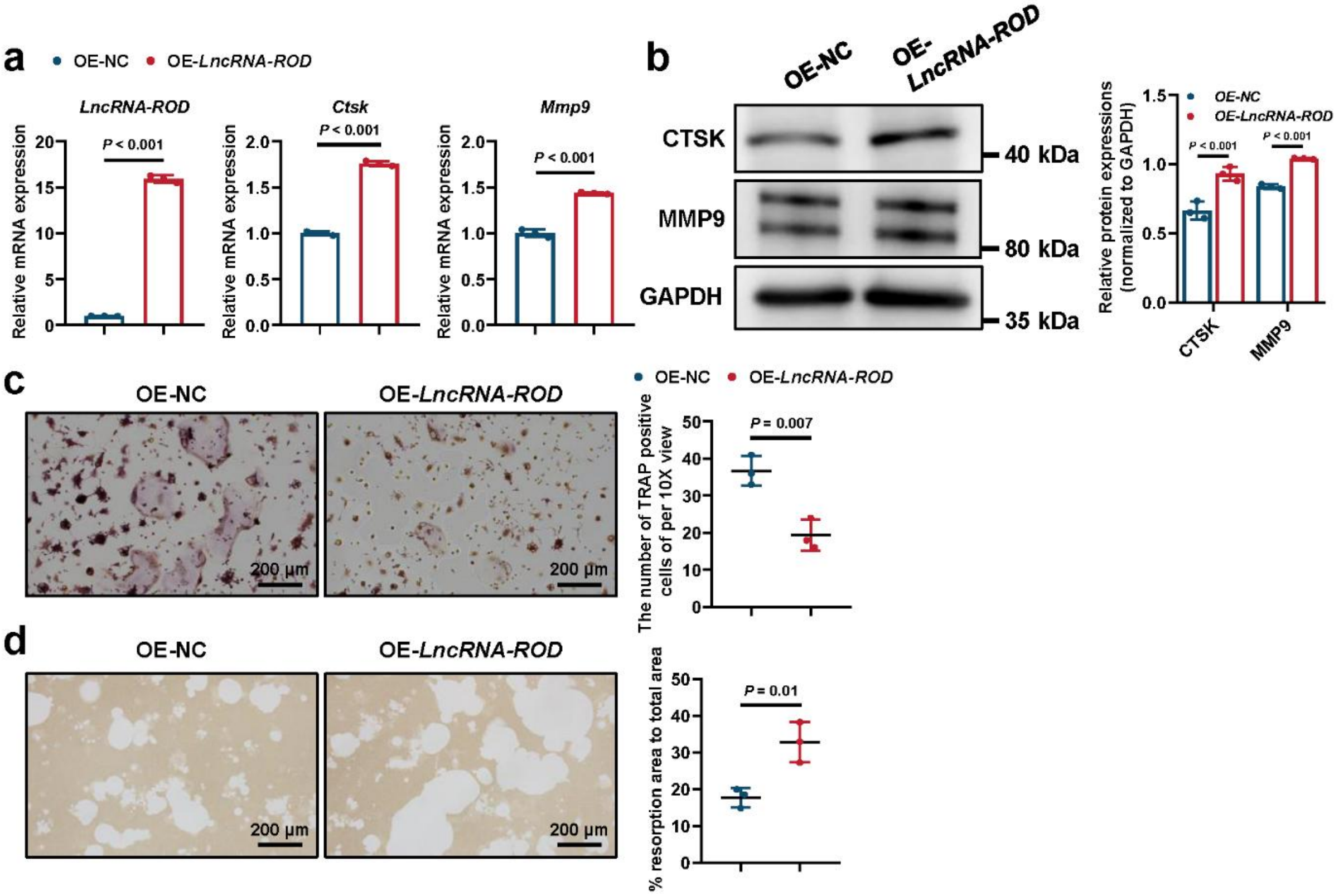

a
OE-NC
OE-*LncRNA-ROD*
LncRNA-ROD
Ctsk
Mmp9
Relative mRNA expression
P < 0.001
b
OE-NC
OE-*LncRNA-ROD*
CTSK
MMP9
GAPDH
40 kDa
80 kDa
35 kDa
Relative protein expressions (normalized to GAPDH)
c
OE-NC
OE-*LncRNA-ROD*
200 μm
The number of TRAP positive cells of per 10X view
P = 0.007
d
% resorption area to total area
P = 0.01

Fig. S9

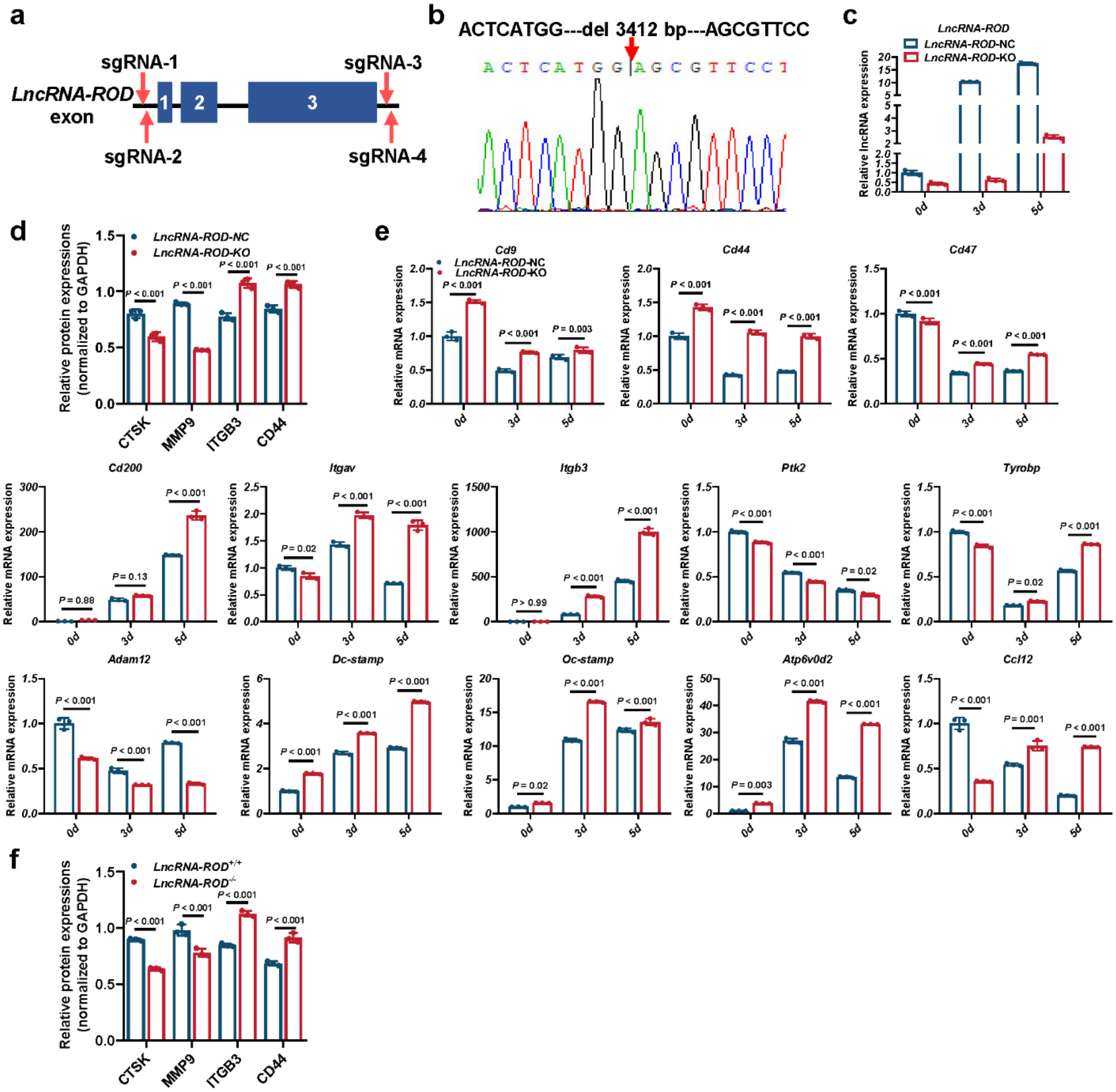
a
sgRNA-1
sgRNA-3
LncRNA-ROD exon
sgRNA-2
sgRNA-4
b
ACTCATGG---del 3412 bp---AGCGTTCC
c
LncRNA-ROD
LncRNA-ROD-NC
LncRNA-ROD-KO
Relative lncRNA expression
d
Relative protein expressions (normalized to GAPDH)
CTSK
MMP9
ITGB3
CD44
e
Cd9
Cd44
Cd47
Cd200
Itgav
Itgb3
Ptk2
Tyrobp
Adam12
Dc-stamp
Oc-stamp
Atp6v0d2
Ccl12
Relative mRNA expression
f
LncRNA-ROD+/+
LncRNA-ROD-/-

Fig. S10

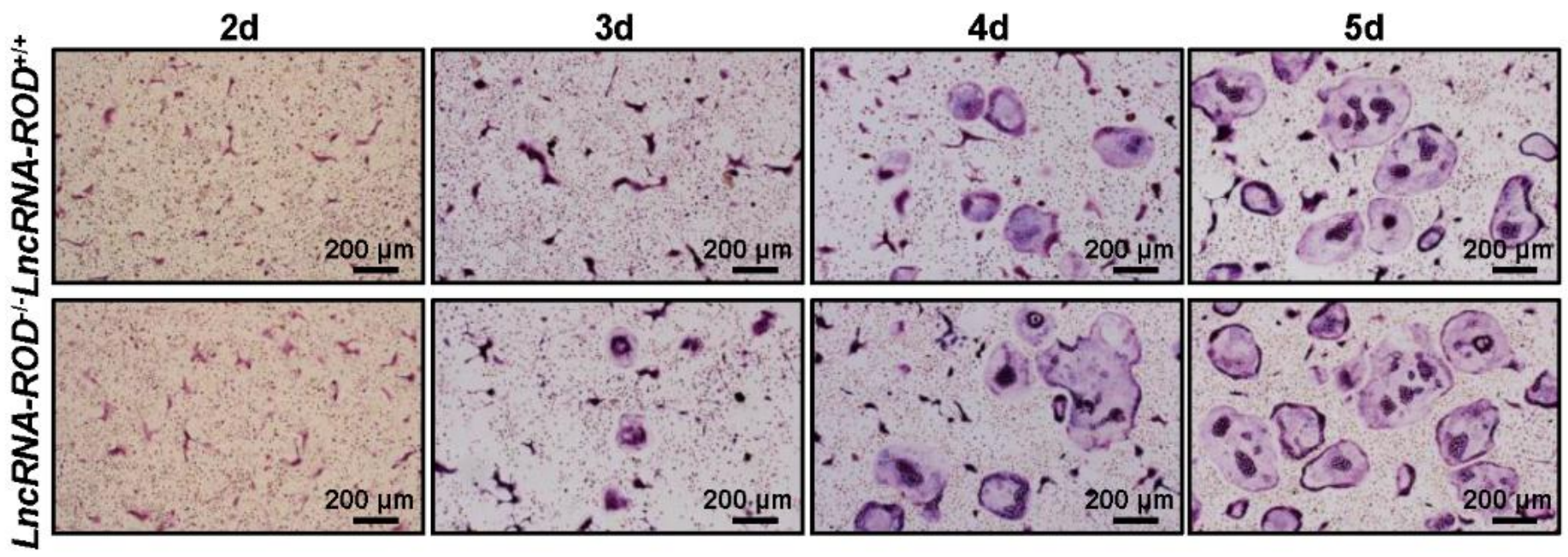

Fig. S11

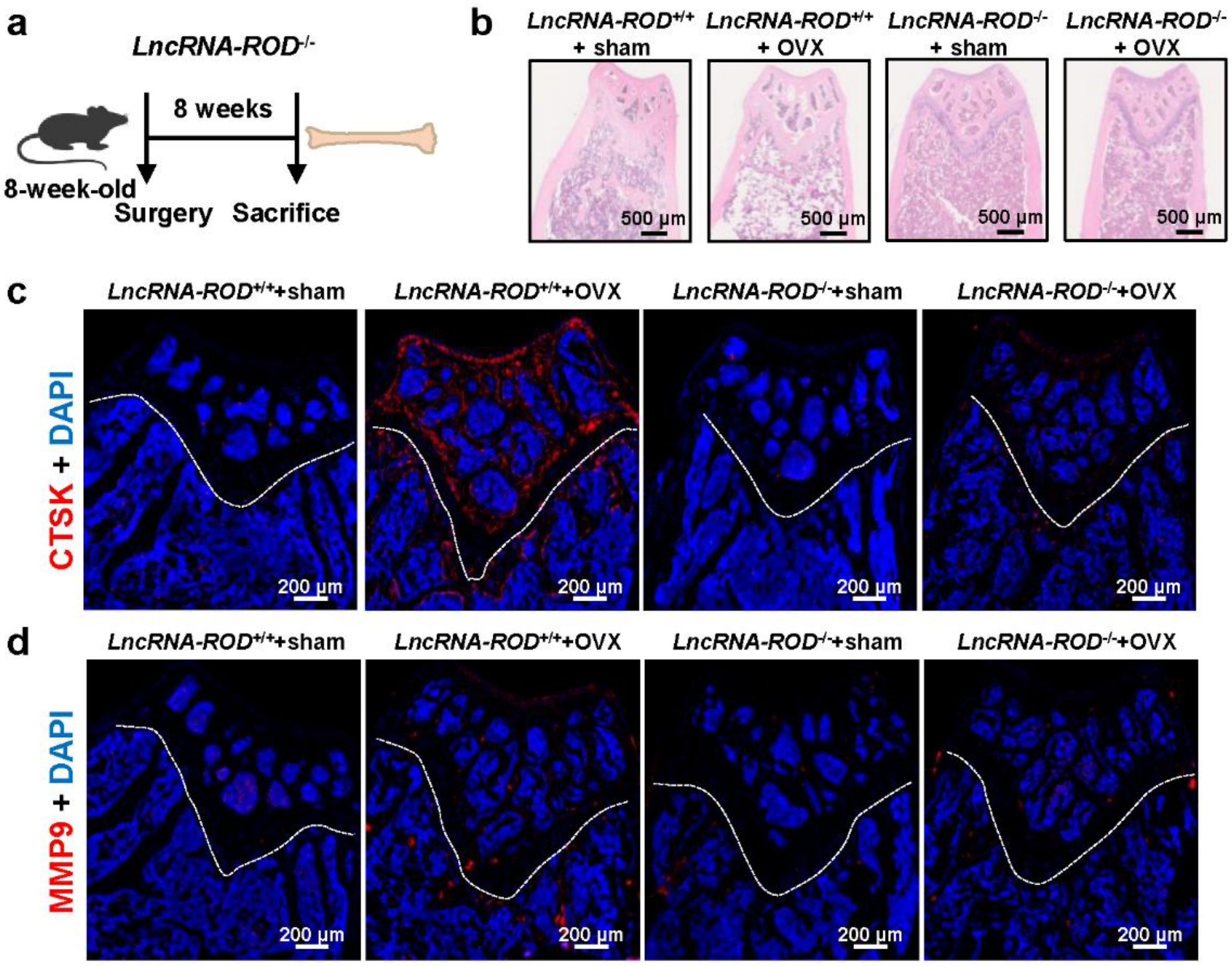
a
LncRNA-ROD-/-
8 weeks
8-week-old
Surgery
Sacrifice
b
LncRNA-ROD+/+ + sham
LncRNA-ROD+/+ + OVX
LncRNA-ROD-/- + sham
LncRNA-ROD-/- + OVX
500 μm
c
LncRNA-ROD+/++sham
LncRNA-ROD+/++OVX
LncRNA-ROD-/-+sham
LncRNA-ROD-/-+OVX
CTSK + DAPI
200 μm
d
LncRNA-ROD+/++sham
LncRNA-ROD+/++OVX
LncRNA-ROD-/-+sham
LncRNA-ROD-/-+OVX
MMP9 + DAPI
200 μm

Fig. S12

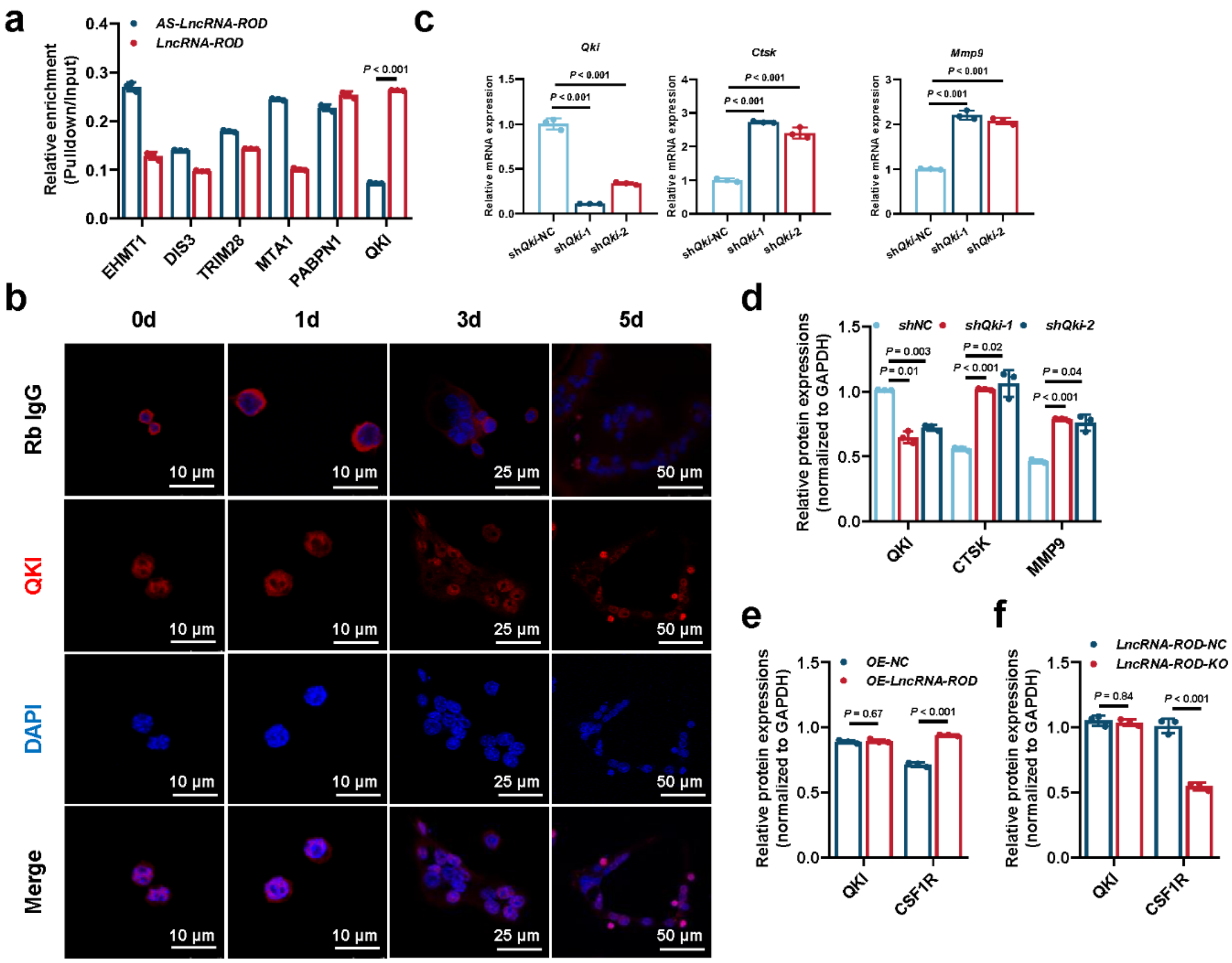

a
AS-LncRNA-ROD
LncRNA-ROD
Relative enrichment (Pulldown/Input)
P < 0.001
EHMT1
DIS3
TRIM28
MTA1
PABPN1
QKI
b
0d
1d
3d
5d
Rb IgG
QKI
DAPI
Merge
10 μm
25 μm
50 μm
c
Qki
Ctsk
Mmp9
Relative mRNA expression
P < 0.001
shQki-NC
shQki-1
shQki-2
d
shNC
shQki-1
shQki-2
Relative protein expressions (normalized to GAPDH)
P = 0.003
P = 0.01
P = 0.02
P < 0.001
P = 0.04
QKI
CTSK
MMP9
e
OE-NC
OE-LncRNA-ROD
P = 0.67
P < 0.001
QKI
CSF1R
f
LncRNA-ROD-NC
LncRNA-ROD-KO
P = 0.84
P < 0.001

Fig. S13

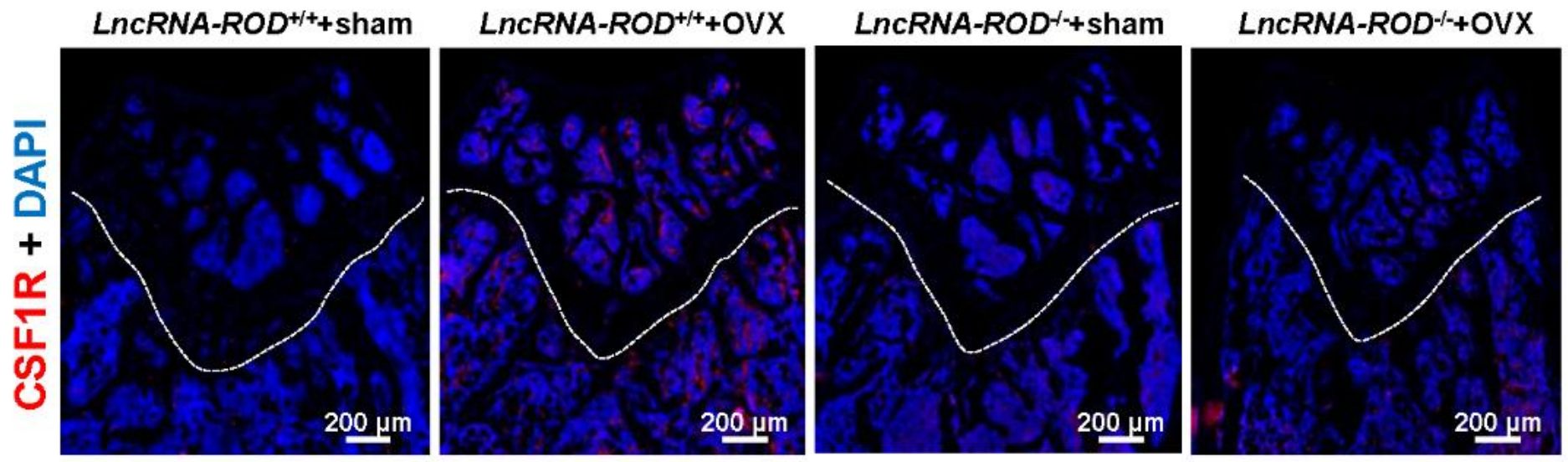

LncRNA-ROD+/+ +sham
LncRNA-ROD+/+ +OVX
LncRNA-ROD-/- +sham
LncRNA-ROD-/- +OVX
CSF1R + DAPI
200 µm
200 µm
200 µm
200 µm

Fig. S14

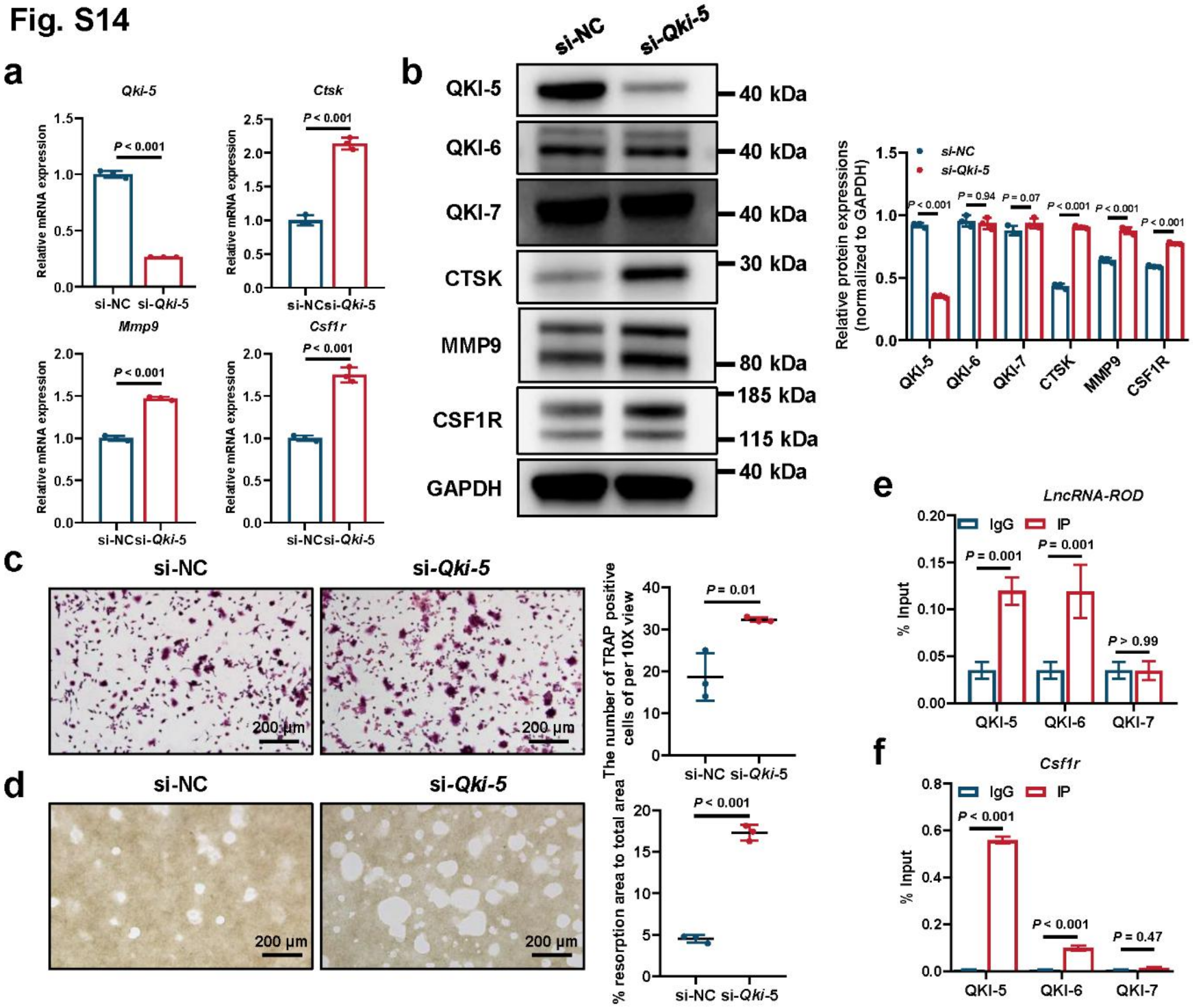
a
Qki-5
Ctsk
Mmp9
Csf1r
Relative mRNA expression
P < 0.001
si-NC
si-Qki-5
b
si-NC
si-Qki-5
QKI-5
QKI-6
QKI-7
CTSK
MMP9
CSF1R
GAPDH
40 kDa
30 kDa
80 kDa
185 kDa
115 kDa
Relative protein expressions (normalized to GAPDH)
P = 0.94
P = 0.07
c
200 μm
The number of TRAP positive cells of per 10X view
P = 0.01
d
% resorption area to total area
e
LncRNA-ROD
IgG
IP
% Input
P = 0.001
P > 0.99
f
Csf1r
P = 0.47

Fig. S15

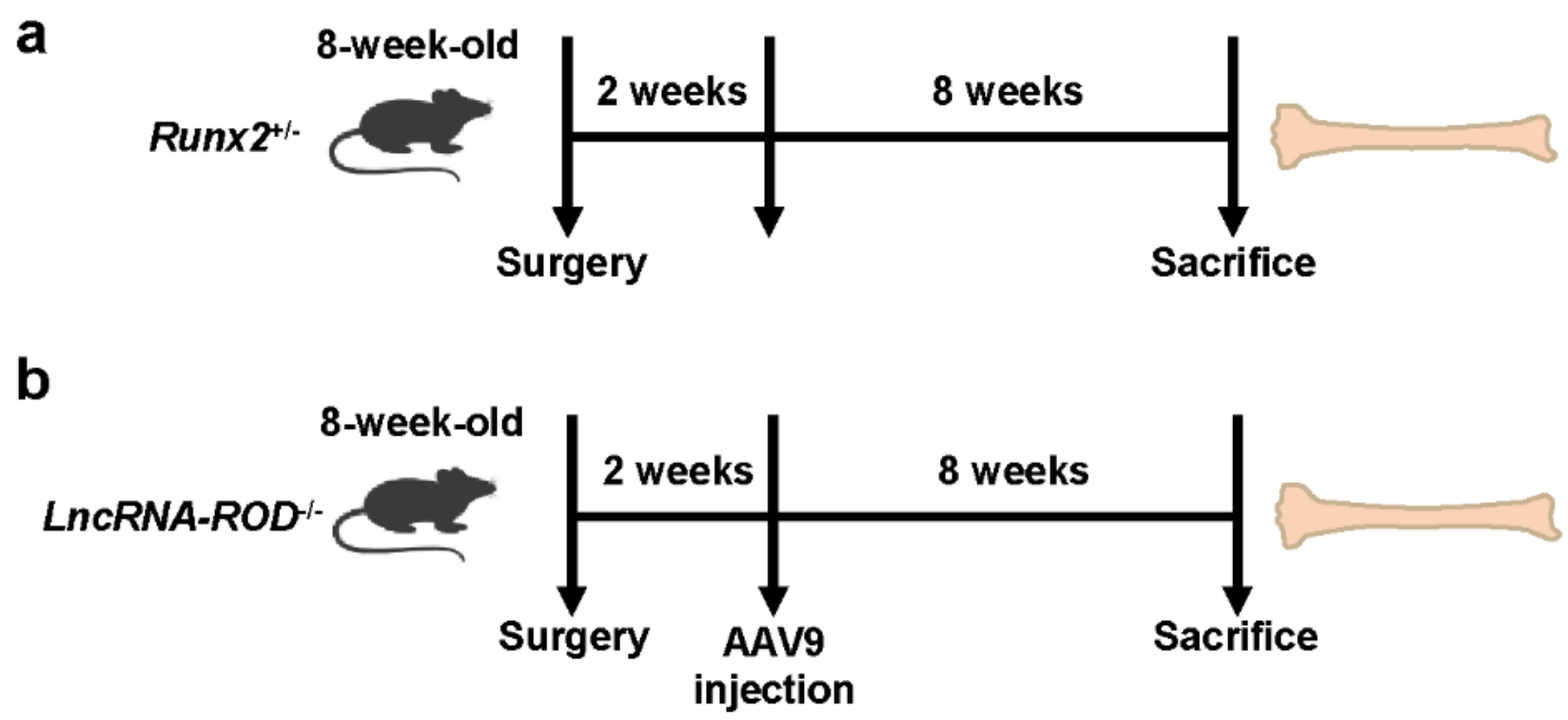
a
8-week-old
Runx2+/-
2 weeks
8 weeks
Surgery
Sacrifice
b
8-week-old
LncRNA-ROD-/-
2 weeks
8 weeks
Surgery
AAV9
injection
Sacrifice

Fig. S16

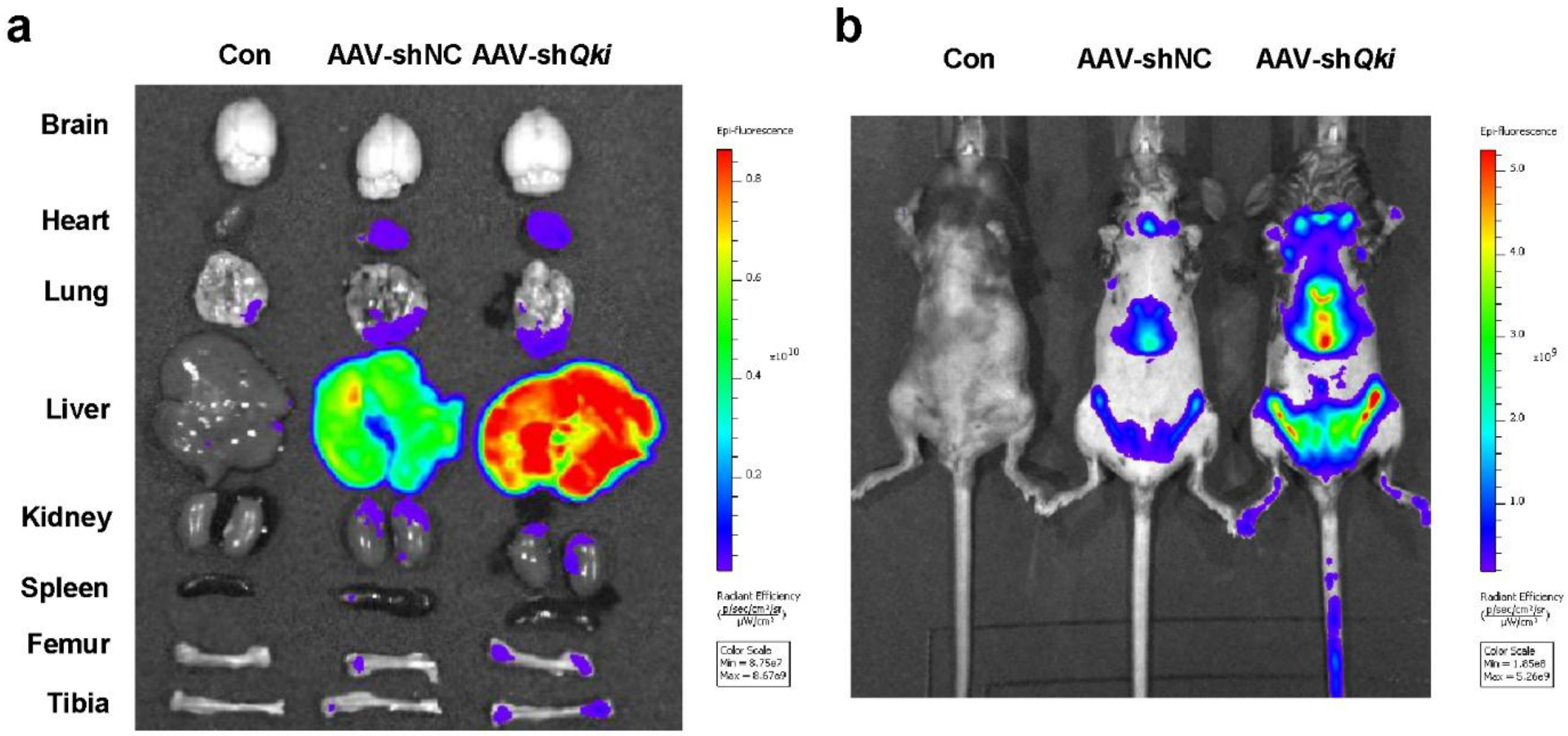
a
Con
AAV-shNC
AAV-sh*Qki*
Brain
Heart
Lung
Liver
Kidney
Spleen
Femur
Tibia
Epi-fluorescence
0.8
0.6
0.4
0.2
x10^10
Radiant Efficiency
(p/sec/cm²/sr / μW/cm²)
Color Scale
Min = 8.75e7
Max = 8.67e9
b
Con
AAV-shNC
AAV-sh*Qki*
Epi-fluorescence
5.0
4.0
3.0
2.0
1.0
x10^9
Radiant Efficiency
(p/sec/cm²/sr / μW/cm²)
Color Scale
Min = 1.65e8
Max = 5.26e9

Fig. S17

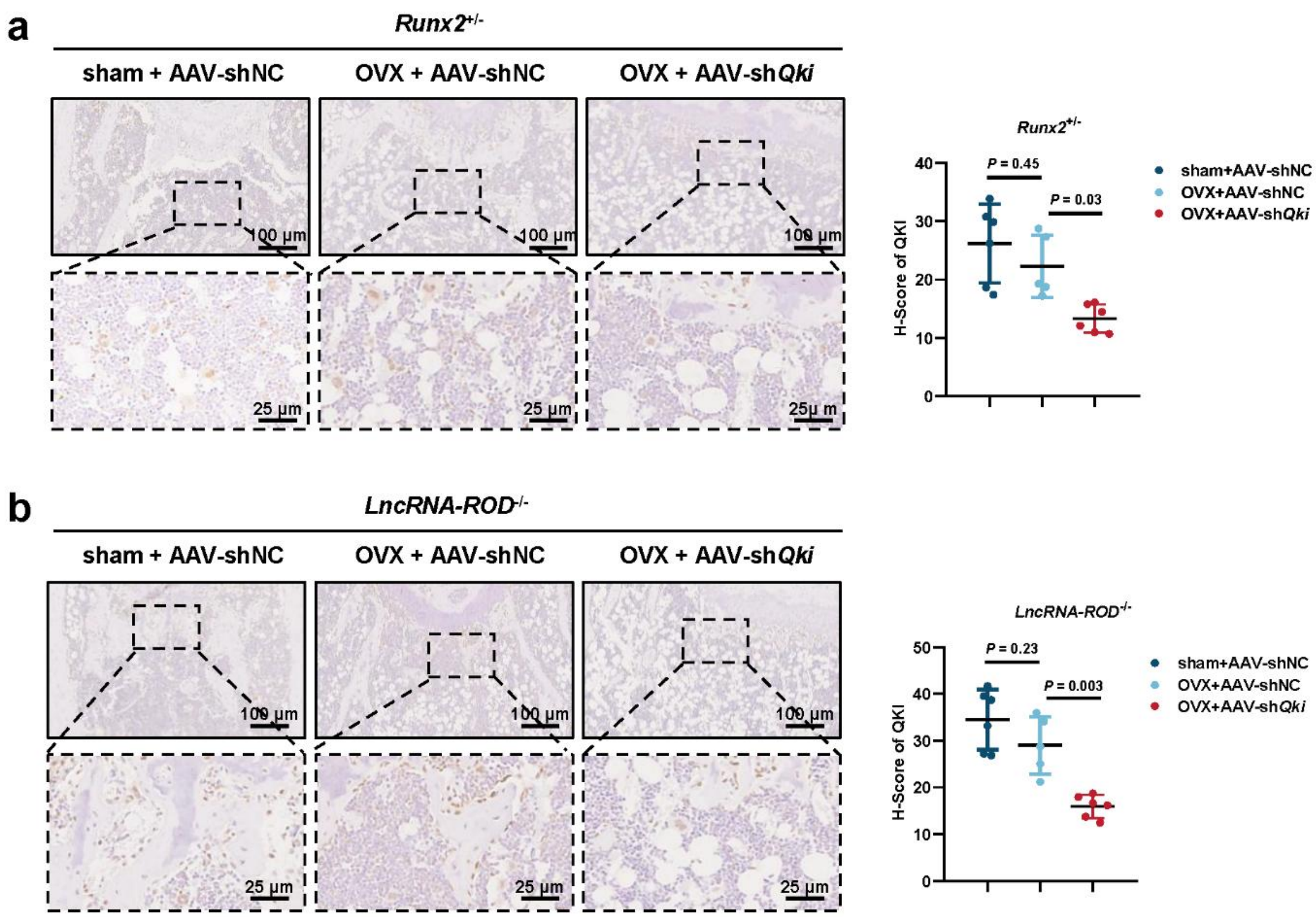
a
Runx2+/-
sham + AAV-shNC
OVX + AAV-shNC
OVX + AAV-shQki
100 μm
25 μm
Runx2+/-
P = 0.45
P = 0.03
H-Score of QKI
sham+AAV-shNC
OVX+AAV-shNC
OVX+AAV-shQki
b
LncRNA-ROD-/-
P = 0.23
P = 0.003

Fig. S18

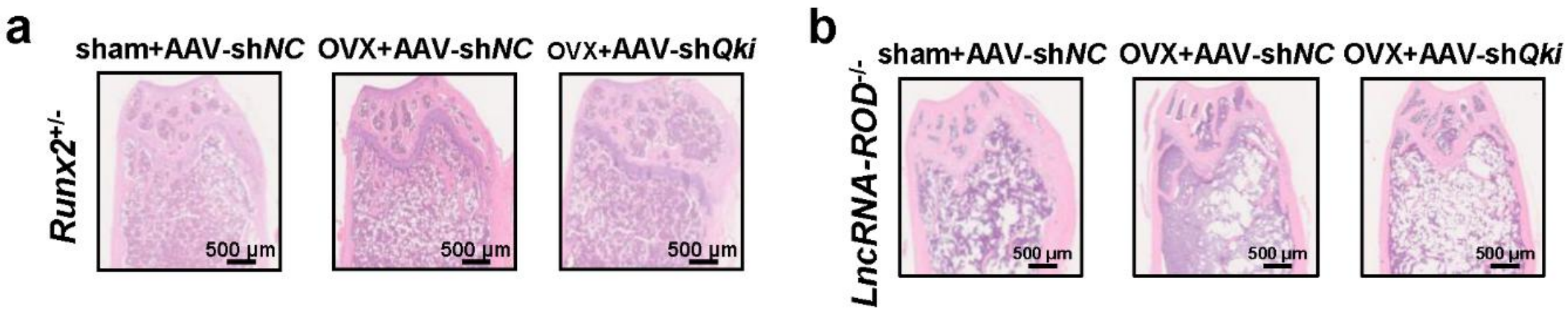
a
sham+AAV-shNC
OVX+AAV-shNC
OVX+AAV-shQki
Runx2+/-
500 μm
500 μm
500 μm
b
sham+AAV-shNC
OVX+AAV-shNC
OVX+AAV-shQki
LncRNA-ROD-/-
500 μm
500 μm
500 μm

Uncropped Blot Image for Figures

Uncropped blot image for Figure 3a

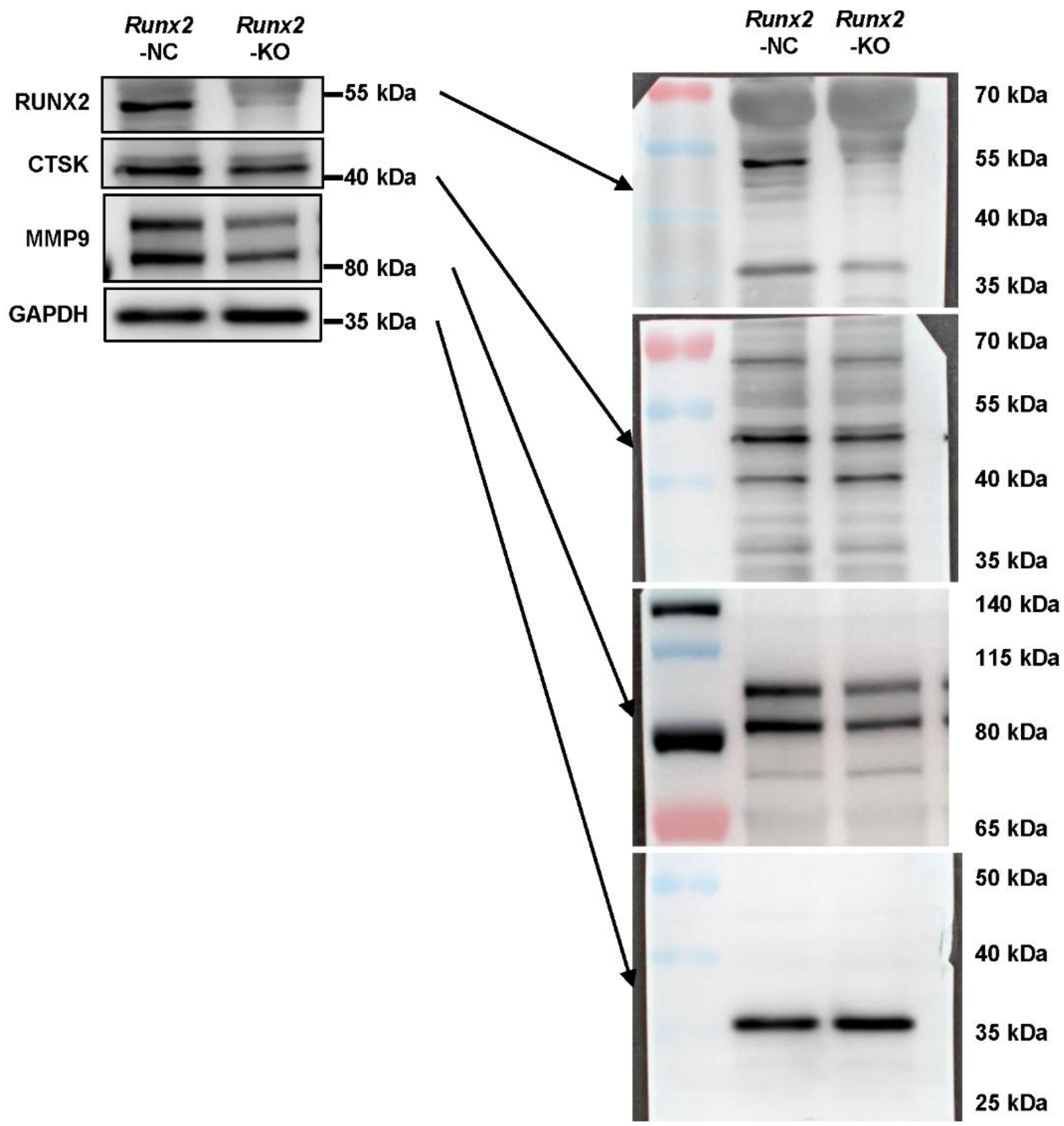

Uncropped blot image for Figure 3d

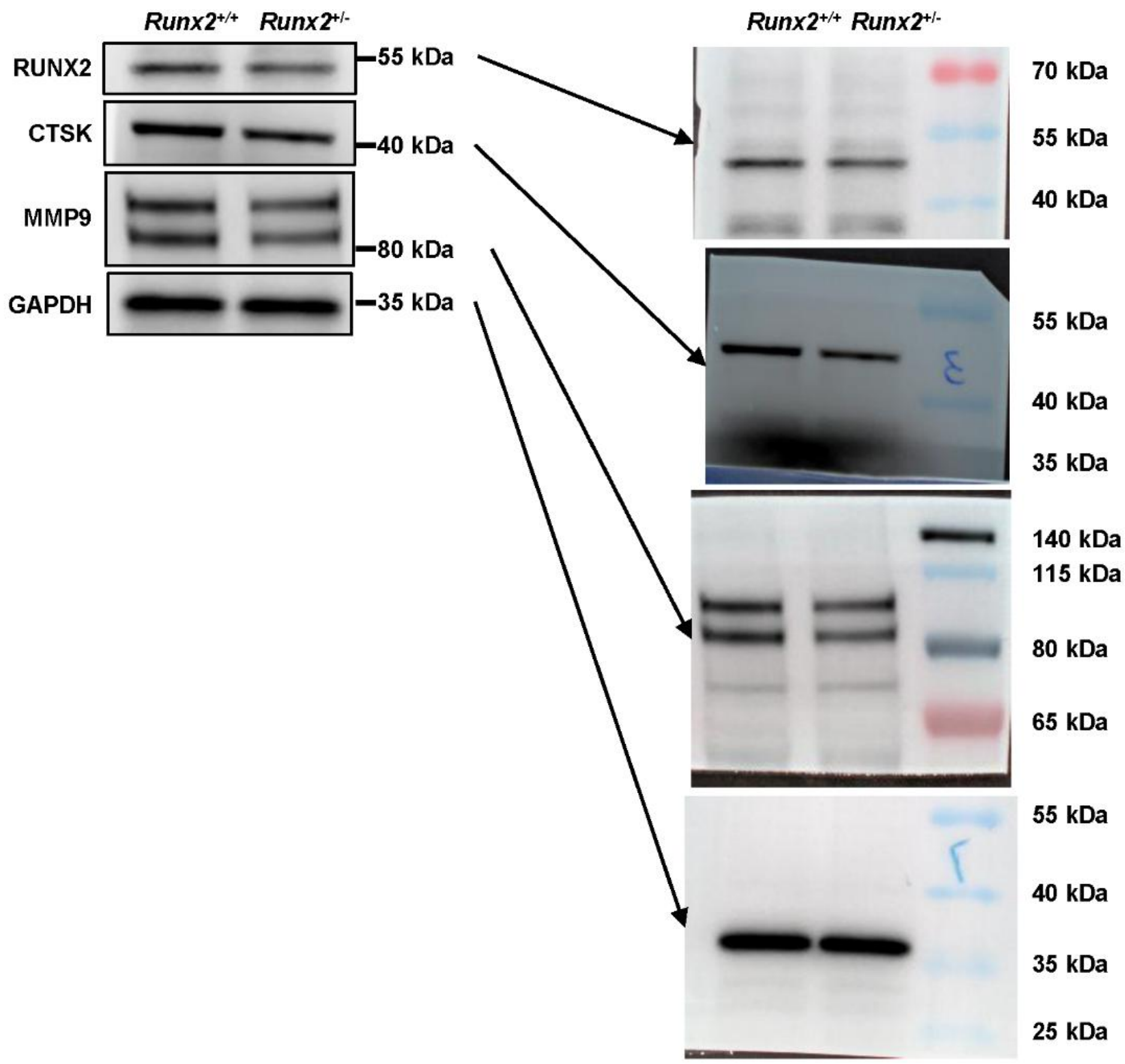

Uncropped blot image for Figure 5a

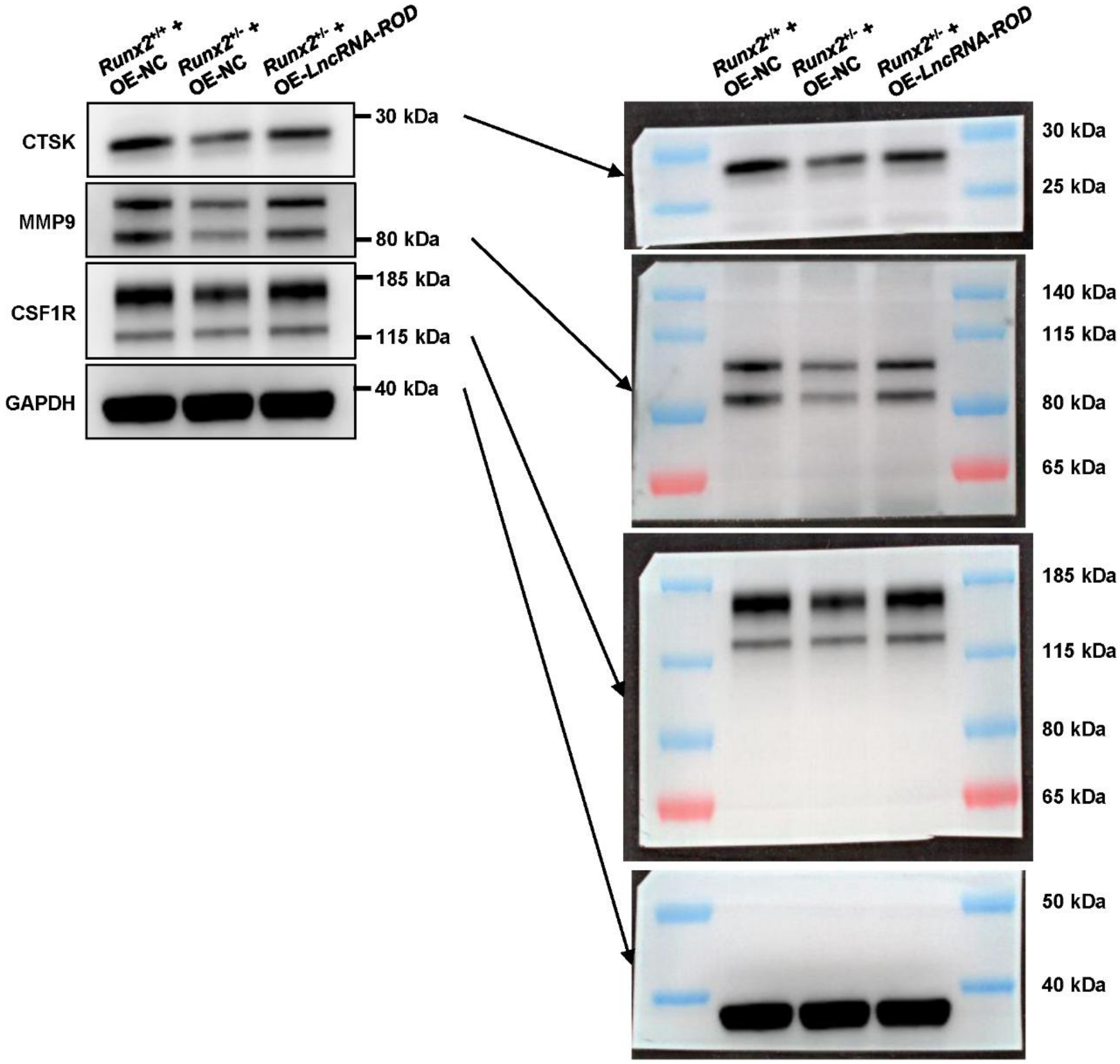

Uncropped blot image for Figure 7a

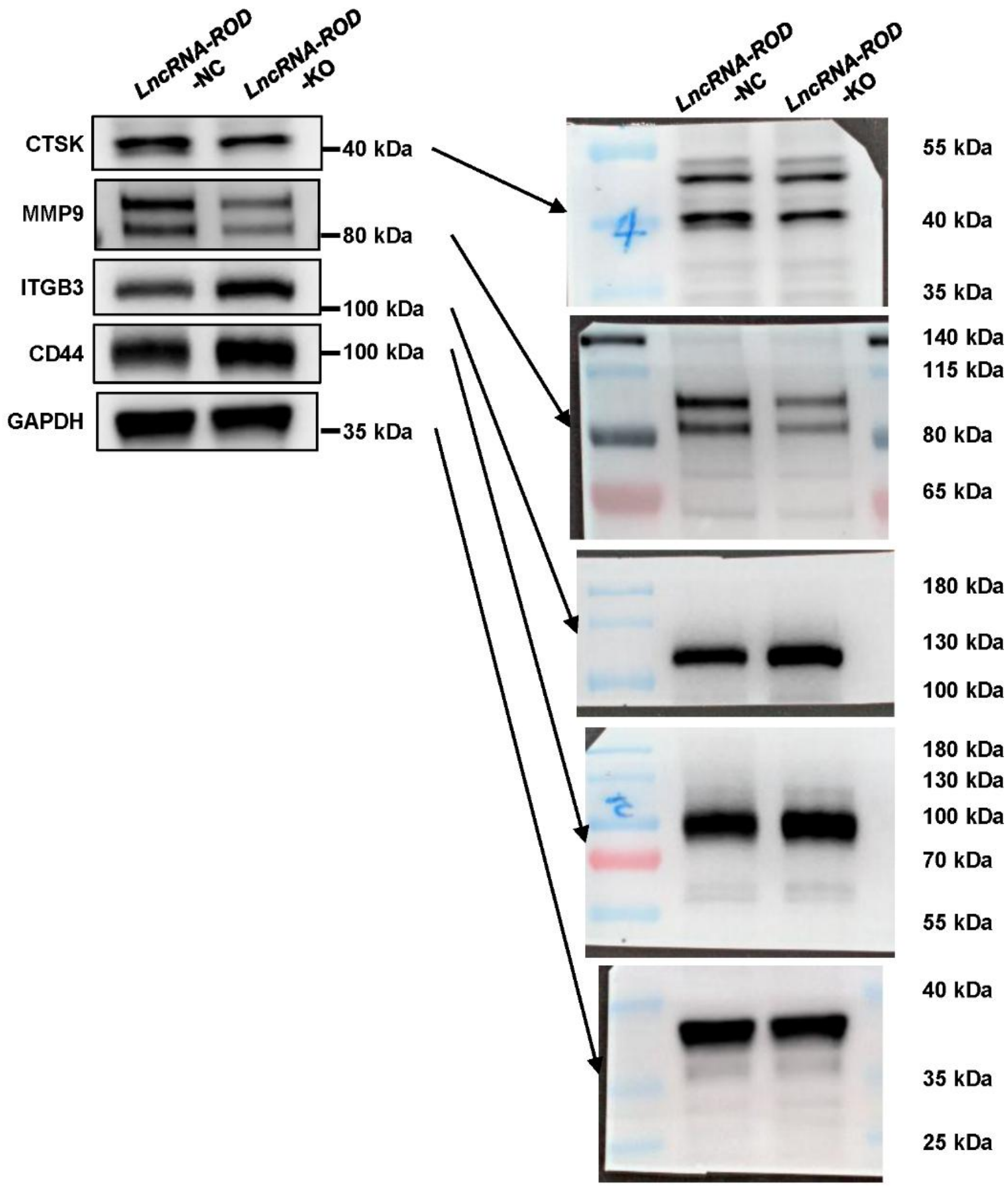

Uncropped blot image for Figure 7d

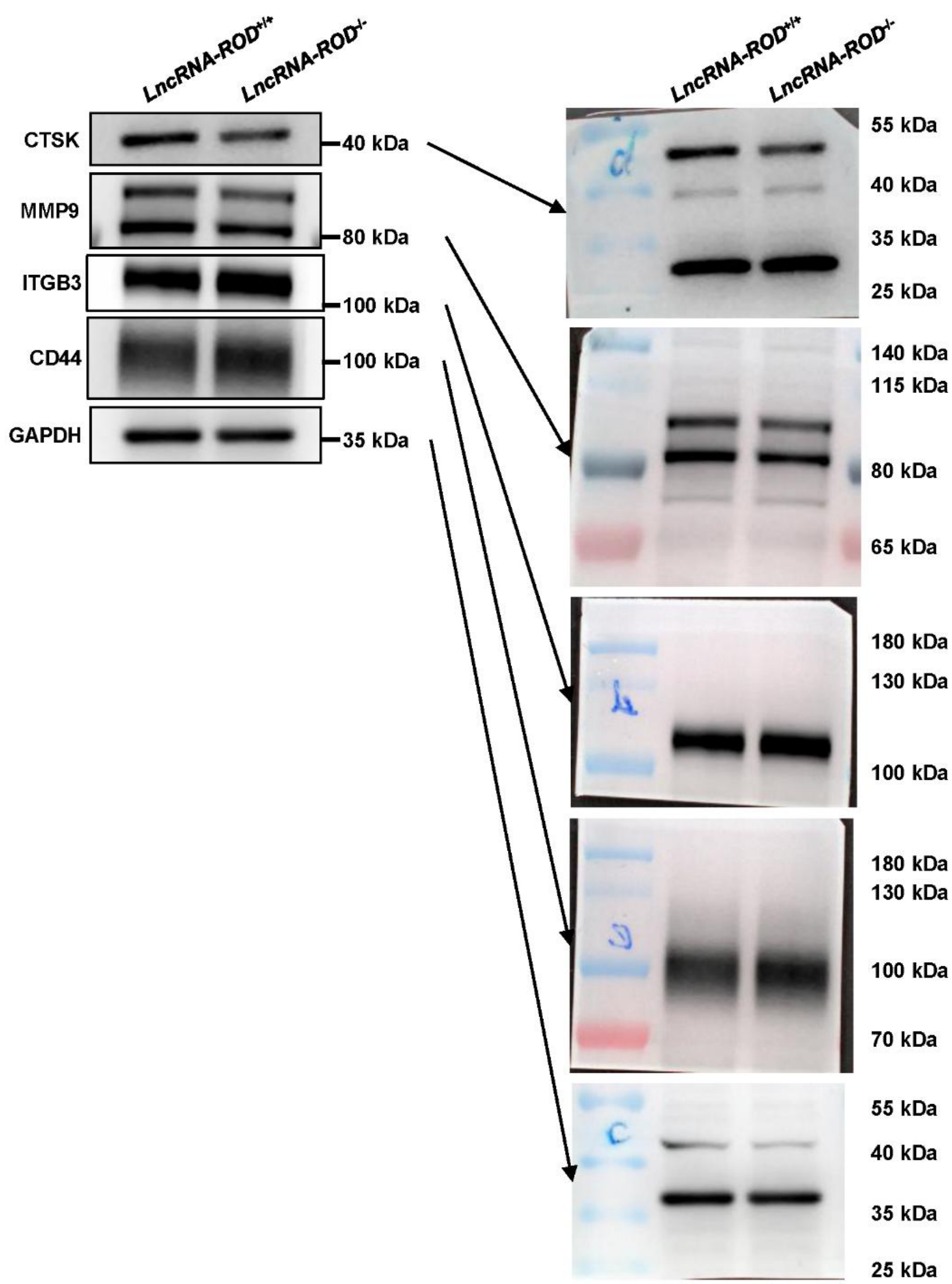

Uncropped blot image for Figure 8a

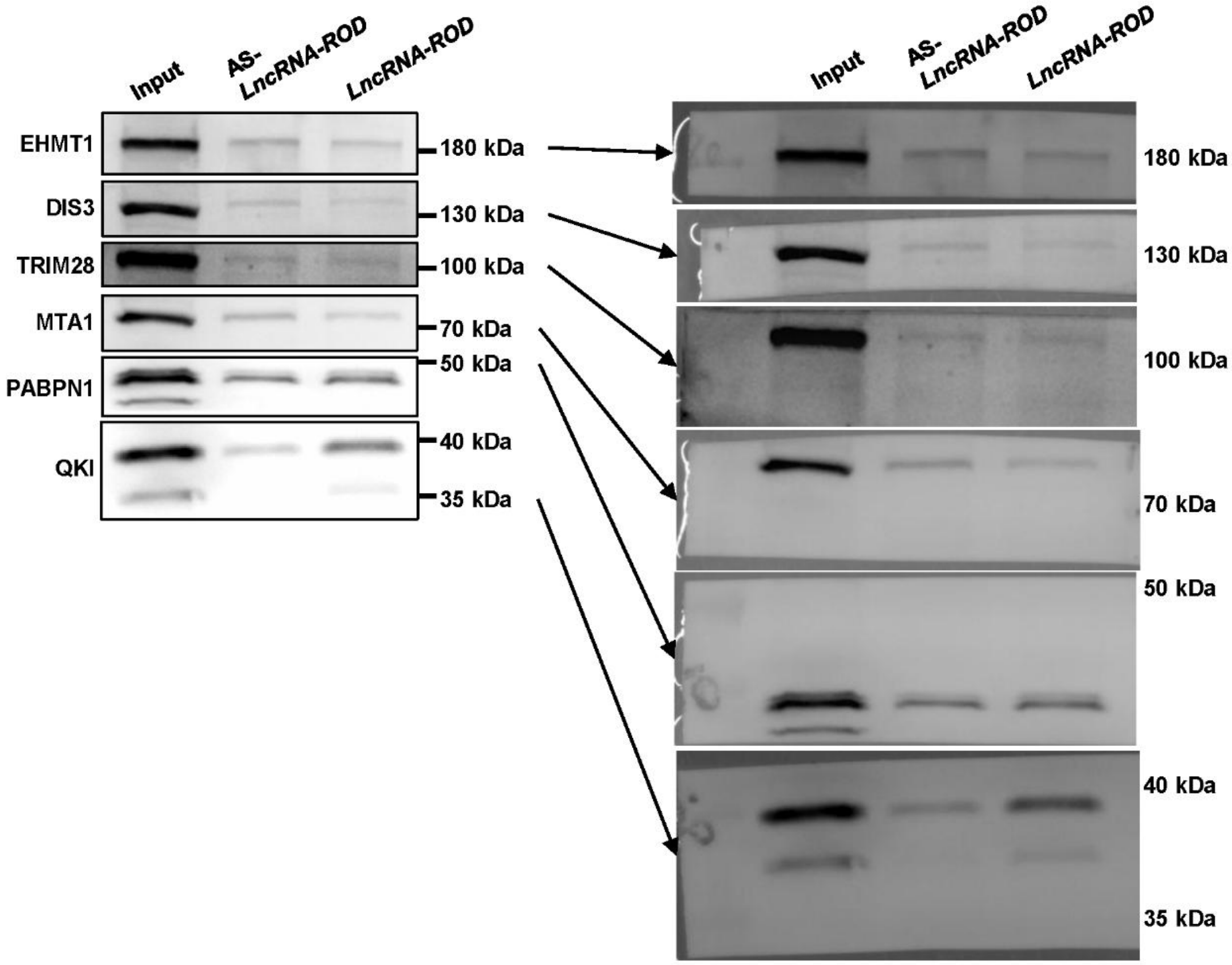

Uncropped blot image for Figure 8c

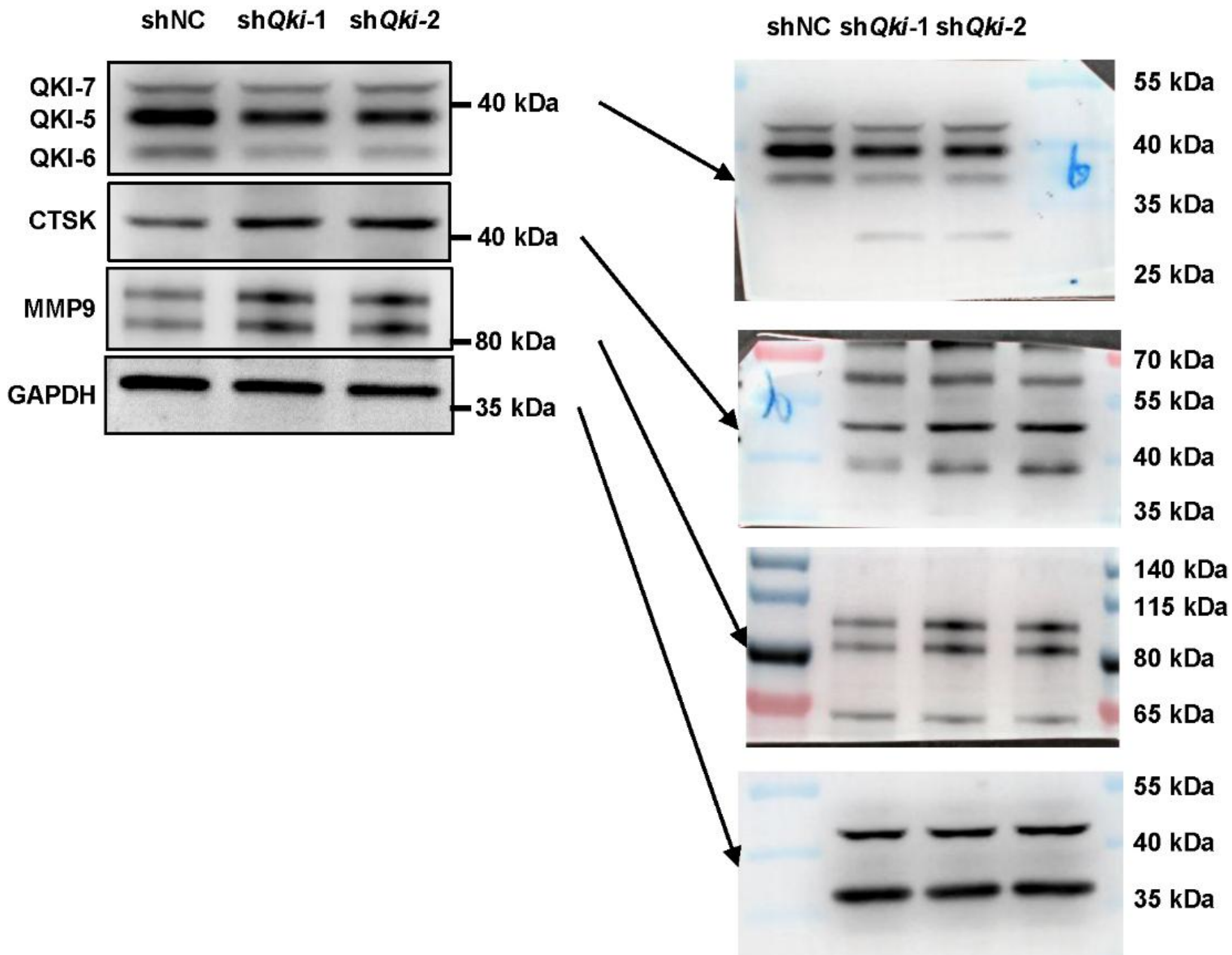

Uncropped blot image for Figure 8f

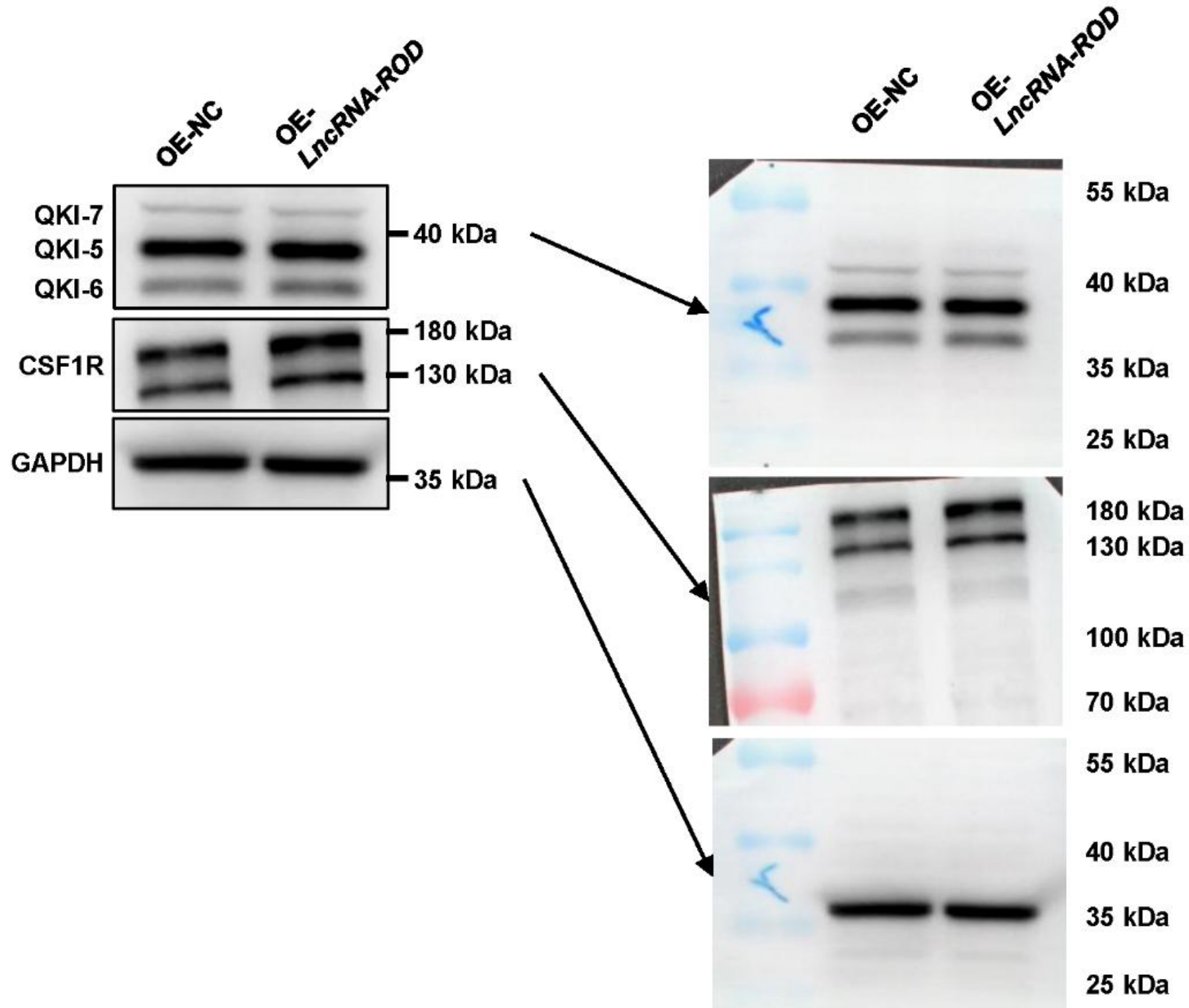

Uncropped blot image for Figure 8g

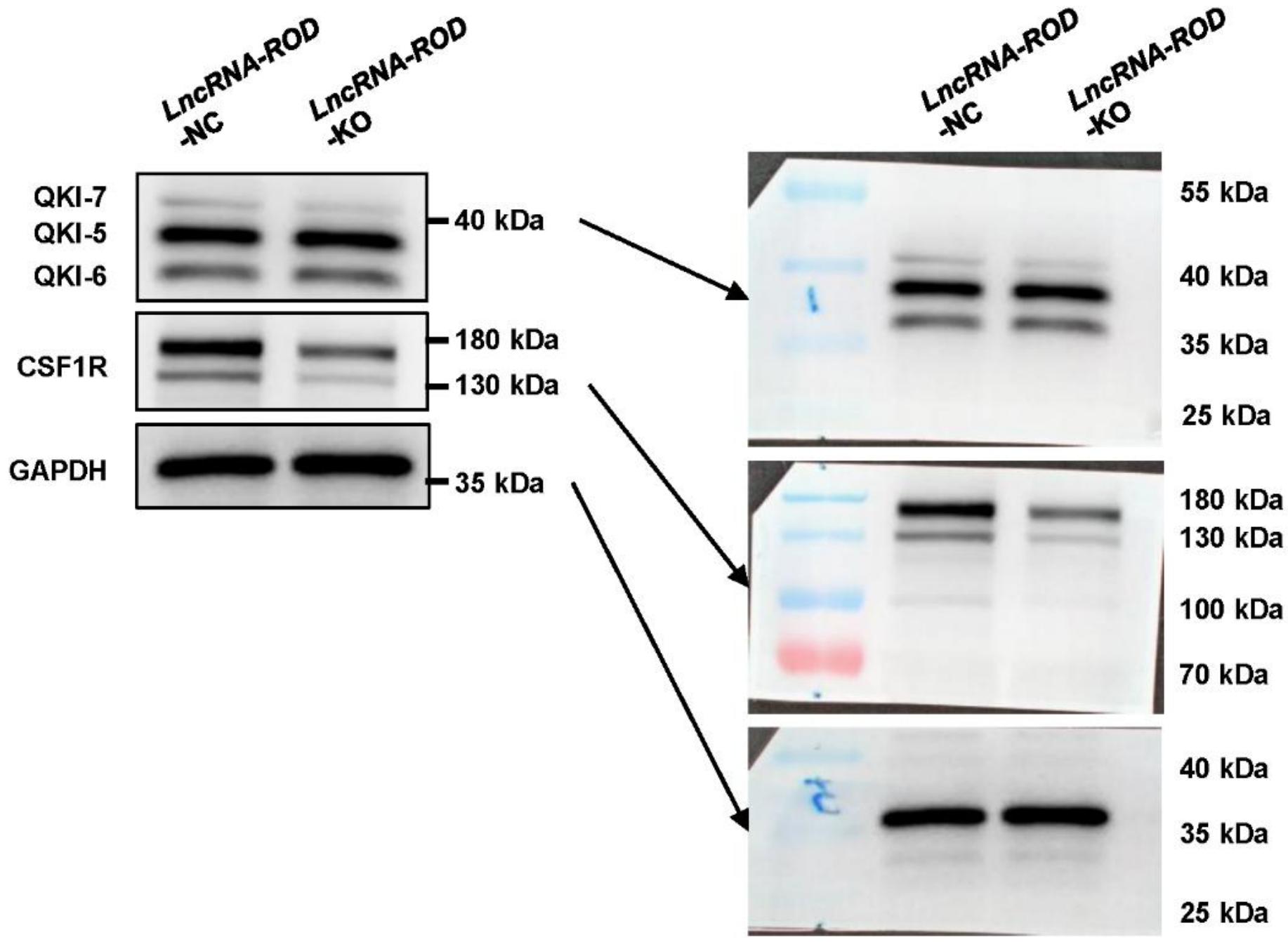

Uncropped blot image for Figure S8b

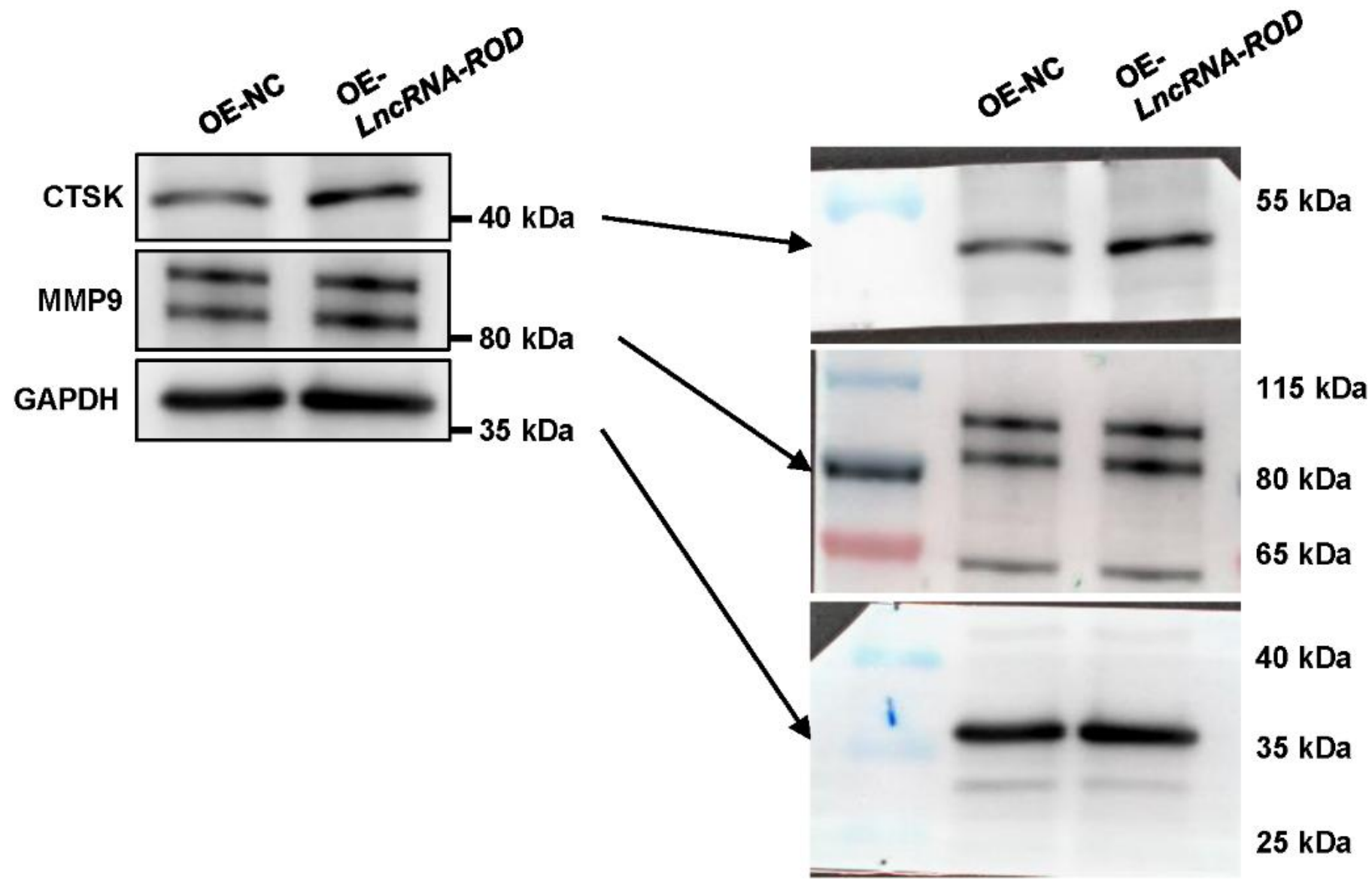

Uncropped blot image for Figure S14b

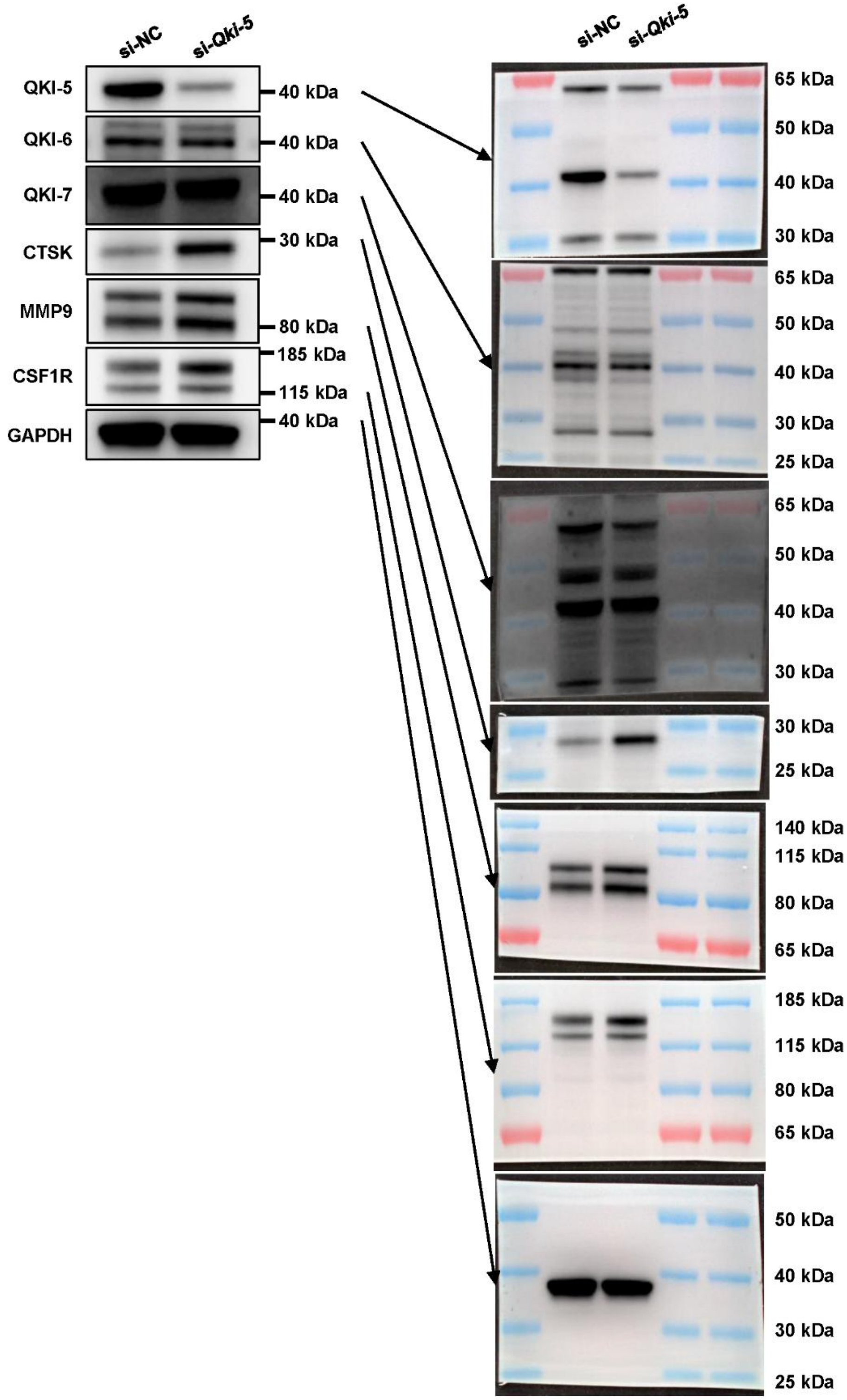

Supplement: Supplementary file 1 [file 41368_2026_466_MOESM1_ESM.pdf]
